# Synthesis of Computationally Designed 2,5(6)-Benzimidazole Derivatives via Pd-Catalyzed Reactions for Potential *E. coli* DNA Gyrase B Inhibition

**DOI:** 10.3390/molecules26051326

**Published:** 2021-03-02

**Authors:** Rafael T. Aroso, Rita C. Guedes, Mariette M. Pereira

**Affiliations:** 1 Coimbra Chemistry Centre, Department of Chemistry, University of Coimbra, Rua Larga, 3004-535 Coimbra, Portugal; rafael.aroso@student.uc.pt; 2 Faculty of Pharmacy, Research Institute for Medicines (iMed.ULisboa), University of Lisbon, Av. Prof. Gama Pinto, 1649-003 Lisbon, Portugal

**Keywords:** computational chemistry, *E. coli* DNA Gyrase B, benzimidazole, cross-coupling, organic catalysis

## Abstract

A pharmacophore model for inhibitors of *Escherichia coli*’s DNA Gyrase B was developed, using computer-aided drug design. Subsequently, docking studies showed that 2,5(6)-substituted benzimidazole derivatives are promising molecules, as they possess key hydrogen bond donor/acceptor groups for an efficient interaction with this bacterial target. Furthermore, 5(6)-bromo-2-(2-nitrophenyl)-1*H*-benzimidazole, selected as a core molecule, was prepared on a multi-gram scale through condensation of 4-bromo-1,2-diaminobenzene with 2-nitrobenzaldehyde using a sustainable approach. The challenging functionalization of the 5(6)-position was carried out via palladium-catalyzed Suzuki–Miyaura and Buchwald-Hartwig amination cross-coupling reactions between *N*-protected-5-bromo-2-nitrophenyl-benzimidazole and aryl boronic acids or sulfonylanilines, with yields up to 81%. The final designed molecules (2-(aminophen-2-yl)-5(6)-substituted-1*H*-benzimidazoles), which encompass the appropriate functional groups in the 5(6)-position according to the pharmacophore model, were obtained in yields up to 91% after acid-mediated N-boc deprotection followed by Pd-catalyzed hydrogenation. These groups are predicted to favor interactions with DNA gyrase B residues Asn46, Asp73, and Asp173, aiming to promote an inhibitory effect.

## 1. Introduction

Imidazole-based heterocycles are a common motif found in both natural and synthetic compounds, present in several clinically approved drugs [1,2,3]. In particular, the benzimidazole moiety is a relevant scaffold for multiple applications in clinic, showing antiulcer, antihypertensive, antiparasitic, anticancer, antifungal, and antibacterial activities [4,5]. For instance, ridinilazole, a recently developed antibacterial containing the benzimidazole scaffold, is currently in phase III clinical trials and has shown great promise for treating Clostridioides difficile infections [6].

**Figure 1:**
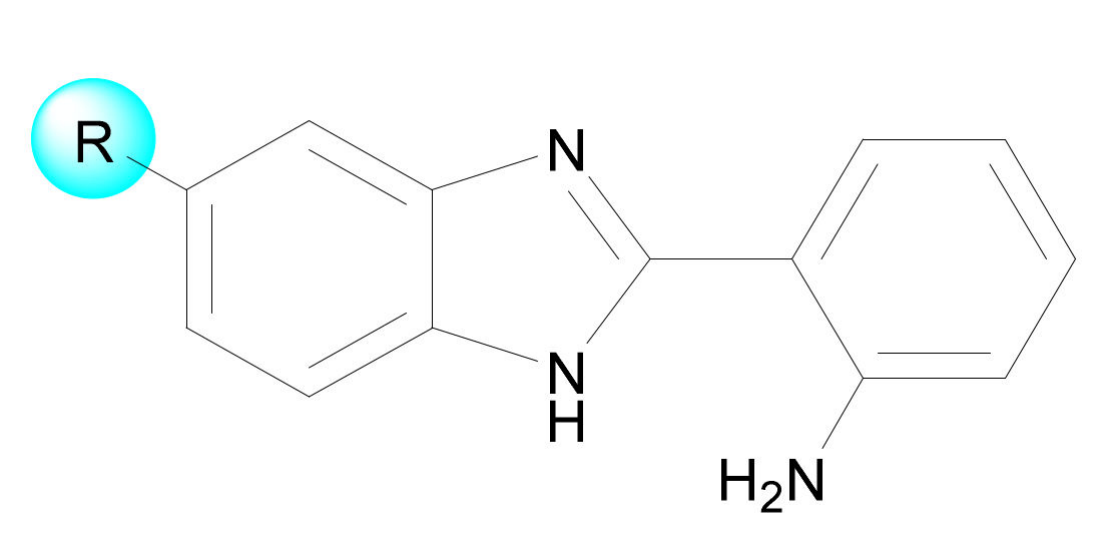
General structure of 2-(2-aminophenyl)-5(6)-substituted-1*H*-benzimidazole molecules holding the key structural functional groups, proposed by the developed pharmacophore model.

Indeed, due to extensive and widespread bacterial resistance to current therapeutics [7] there is an urgent need to develop more efficient synthetic processes to obtain potential new antibiotics derived from a computer-aided rational design. Aiming for the development of inhibitors for the bacterial target Escherichia coli’s DNA Gyrase B [3,8,9,10], we have used a pharmacophore model created in the Molecular Operating Environment (MOE) molecular design software (Chemical Computing Group) [11] to provide insights into the ideal structure of potential antibacterial molecules. Following the analysis of the computational pharmacophore model herein described, we have planned the synthesis of families of potential antibacterial molecules derived from the 1*H*-benzimidazole scaffold (Figure 1). This core was selected as a starting point for our studies, owing to its recently reported antibacterial activity [6] and its synthetic amenability [12].

We hypothesized that the synthesis of 5(6)-halogenated-2-substituted benzimidazole by classic methods [13,14,15] involving a condensation reaction of a halogenated *ortho*-phenylenediamine with 2-nitrocarbonyl/carboxylphenyl would allow the preparation of a core synthon for further derivatization to match the features predicted by the pharmacophoric model.

The limitations imposed by classic multi-step methods [16,17] on the functionalization of 5(6) position of benzimidazoles have driven our efforts towards the use of palladium-catalyzed coupling reactions, namely Suzuki-Miyaura and Buchwald-Hartwig. Current literature on this topic shows multiple reports of palladium-catalyzed reactions involving the oxidative addition to activated 2-halogen substituted benzimidazoles [18,19,20,21,22,23,24,25,26], or 2-aminobenzimidazoles [27], however there are only a few examples describing their application for functionalization of the less reactive 5(6) position. These examples include Suzuki–Miyaura reactions [28,29] using vinyltrifluoroboronates or benzyltrifluoroborates as coupling agents, which, in general, do not include an appropriate hydrogen-bond acceptor according to our model, and/or require multi-step processes for their preparation. There are also a few examples regarding the catalytic amination at the 5(6) position [30,31,32,33,34], but some challenges remain to be overcome, particularly the enhancement of the low reaction yields, and/or the need for huge amounts of expensive catalysts. In this paper, we first describe the computer-aided design of 5(6)-substituted-2-(2-aminophenyl)benzimidazole derivatives aiming at the development of potential *E. coli* GyrB inhibitors. In addition, we report optimized synthetic processes for preparing these newly designed benzimidazole families, which encompass the appropriate substituents, via catalytic modulation of the less explored 5(6)-positions, using benchmark palladium-catalyzed reactions, namely Suzuki–Miyaura and Buchwald–Hartwig couplings with good yields.

## 2. Results and Discussion

### 2.1. Computer-Aided Design of Benzimidazole Derivatives with Potential E. coli DNA GyrB Inhibitory Activity

**Figure 2:**
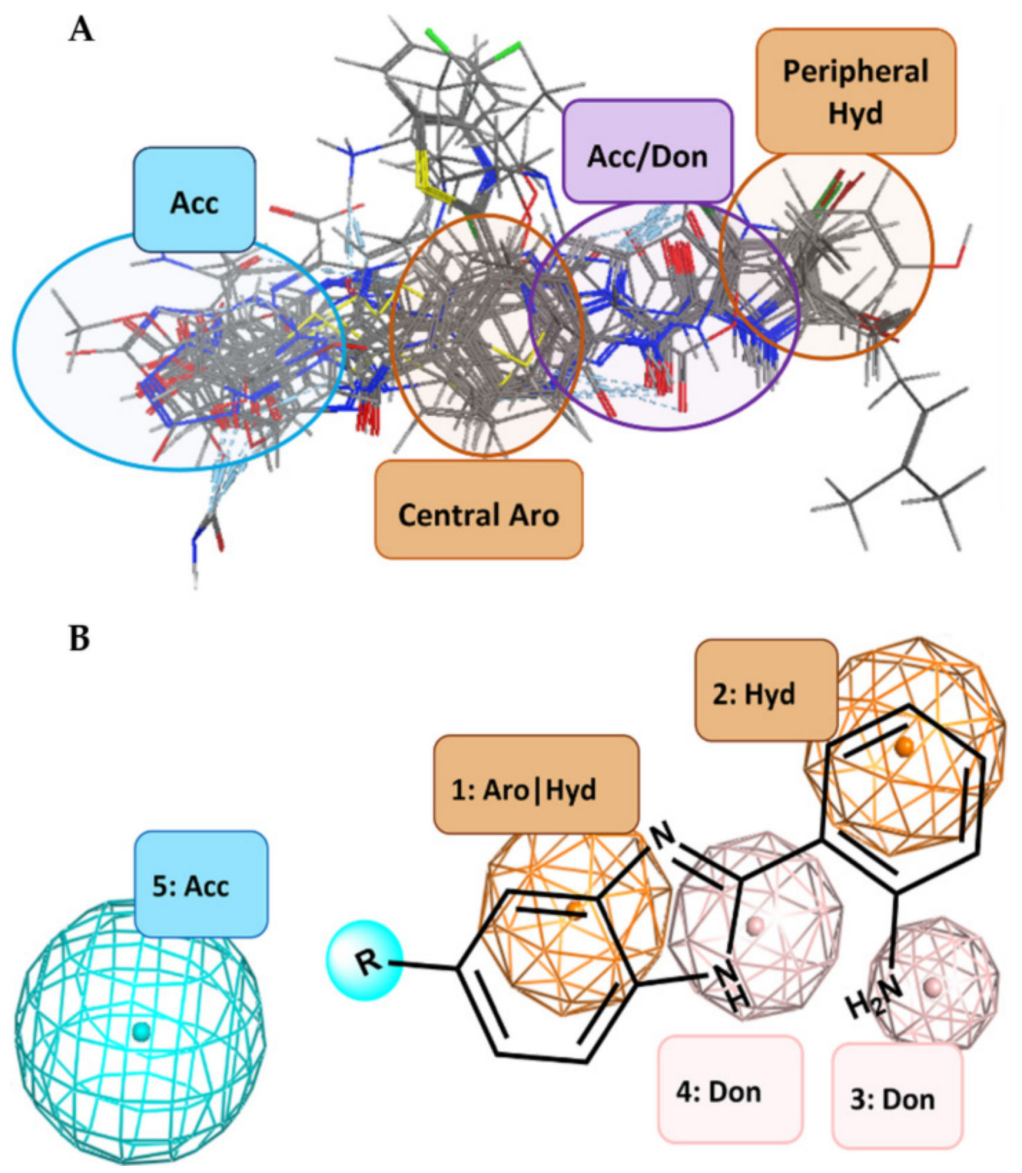
(**A**) Structural alignment of the 18 ligands from the training set and visual identification of common structural features. (**B**) Superimposition of the 2-(2-aminophenyl)-5(6)-substituted-benzimidazole scaffold with the selected pharmacophore model. Acc-Hydrogen bond acceptor; Aro-Aromatic; Don-Hydrogen bond donor; Hyd-Hydrophobic. R = (methylsulfonyl)phenyl, (methoxycarbonyl)phenyl and methoxyphenyl.

To generate the pharmacophore model, an alignment of the 18 training set molecules (see Supplementary Materials: Figure S2) through a stochastic conformer search was performed in MOE (Chemical Computing Group) [11] (Figure 2A).

The common structural features were identified, from which several pharmacophore queries were generated and further refined (by varying feature types, number of features and their radius). The selection and validation of the final pharmacophore model were grounded on its performance against a dataset (test set) composed of 90 compounds [9,10,35,36,37,38,39,40,41] whose activity is well-known (61 active and 30 inactive compounds) (see Supplementary Materials: Figure S3). The best pharmacophore query was generated using MOE’s Unified scheme, and contains five features: (i) a hydrogen bond acceptor region; (ii) an aromatic or hydrophobic region; (iii) one hydrophobic region; and (iv) two hydrogen-bond donor regions. This model (Figure 2B) accurately predicted 90% of the active compounds (from the test set), with only 5% false positives. Figure 2B shows the optimized pharmacophore model superimposed with the selected benzimidazole scaffold bearing an –NH_2_ (hydrogen bond donor) at 2-position and either (methylsulfonyl)phenyl, (methoxycarbonyl)phenyl and methoxyphenyl (hydrogen bond acceptors) at 5(6)-positions.

Our next goal was to determine which type of functional groups are best suited to introduce in the 5(6)- position of the benzimidazole ring. To achieve this goal, we generated a virtual library of 2-(2-aminophenyl)-5(6)-substituted-benzimidazole derivatives (in total, 6681 compounds), using MOE tools. Initially, we screened the virtual library using the pharmacophore model, which we had previously selected and validated, in order to remove those derivatives whose features did not have hydrogen-bond acceptors. Next, docking studies were performed, using *E. coli*’s DNA GyrB ATPase binding site (PDB entry 4KFG) [42], in order to identify the derivatives with the most potential for effective inhibition of this enzyme. Briefly, using GOLD (Cambridge Crystallographic Data Centre) [43] and the ChemScore scoring function, we ranked the compounds with the highest potential. ChemScore is a fitness (or scoring) function implemented in GOLD software to estimate the receptor-ligand binding affinity. This scoring function includes several terms, namely, a protein-ligand atom clash term and an internal energy term, taking account of hydrophobic-hydrophobic contact area, hydrogen bonding, ligand flexibility, and metal interaction. Then, we compared the results to a correlation established between ChemScore values and biological activies of known inhibitors (see Supplementary Materials: Figure S3). Based on this, we selected some of the highest ranking compounds based on their synthetic feasibility (Table 1).

**Figure 3:**
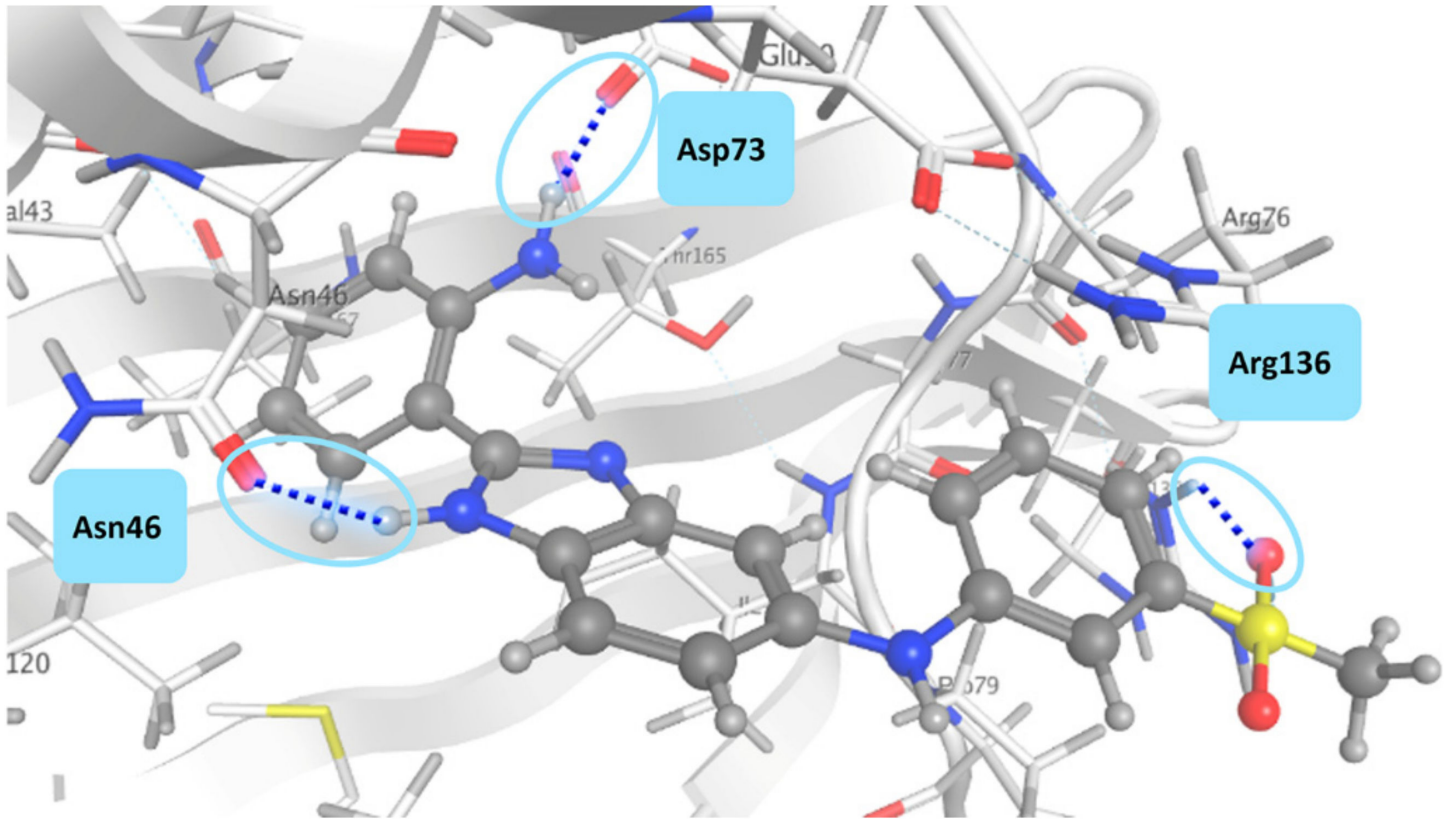
Docking binding mode of compound **15**, as predicted by GOLD using the ChemScore function, in the ATP binding site of *E. coli* DNA gyrase B (PDB entry 4KFG). The protein is represented in white, with the exception of the relevant neighboring side-chains, which, along with the ligand, are color-coded according to atom type: Blue = N; Red = O, Yellow = S; Dark Grey = C; Light Grey = H. Hydrogen bonds are indicated by blue dotted lines, and relevant protein residues are highlighted.

As an example, Figure 3 shows the docking pose obtained for the highest scoring compound **15** (Table 1, entry 1).

From the analysis of the best scoring docking poses, we can observe three relevant hydrogen bond interactions: two between the –NH groups and Asn46 and Asp73; and another between the S=O group and Arg136. In addition, there are hydrophobic interactions between the aminophenyl ring and the surrounding non-polar protein side-chains. This corroborates the information obtained by the pharmacophore model as it states the importance of having hydrogen bond donors and acceptors in specific portions of the molecule, as well as aromatic/hydrophobic portions.

In sum, our aim to synthesize new families of 2-(2-aminophenyl)-5(6)-substituted-benzimidazoles is explained by the need to insert hydrogen bond donor groups at 2-position, while modulation of the 5(6)-position will allow the insertion of hydrogen bond acceptor groups. These groups will favor interactions with Asp73 and Arg136, respectively, and therefore increase their inhibition potential for *E. coli*’s DNA gyrase B.

### 2.2. Synthetic Methods for the Preparation of 2-Aminophenyl-5(6)-Substituted Benzimidazole Derivatives

**Scheme 1:**
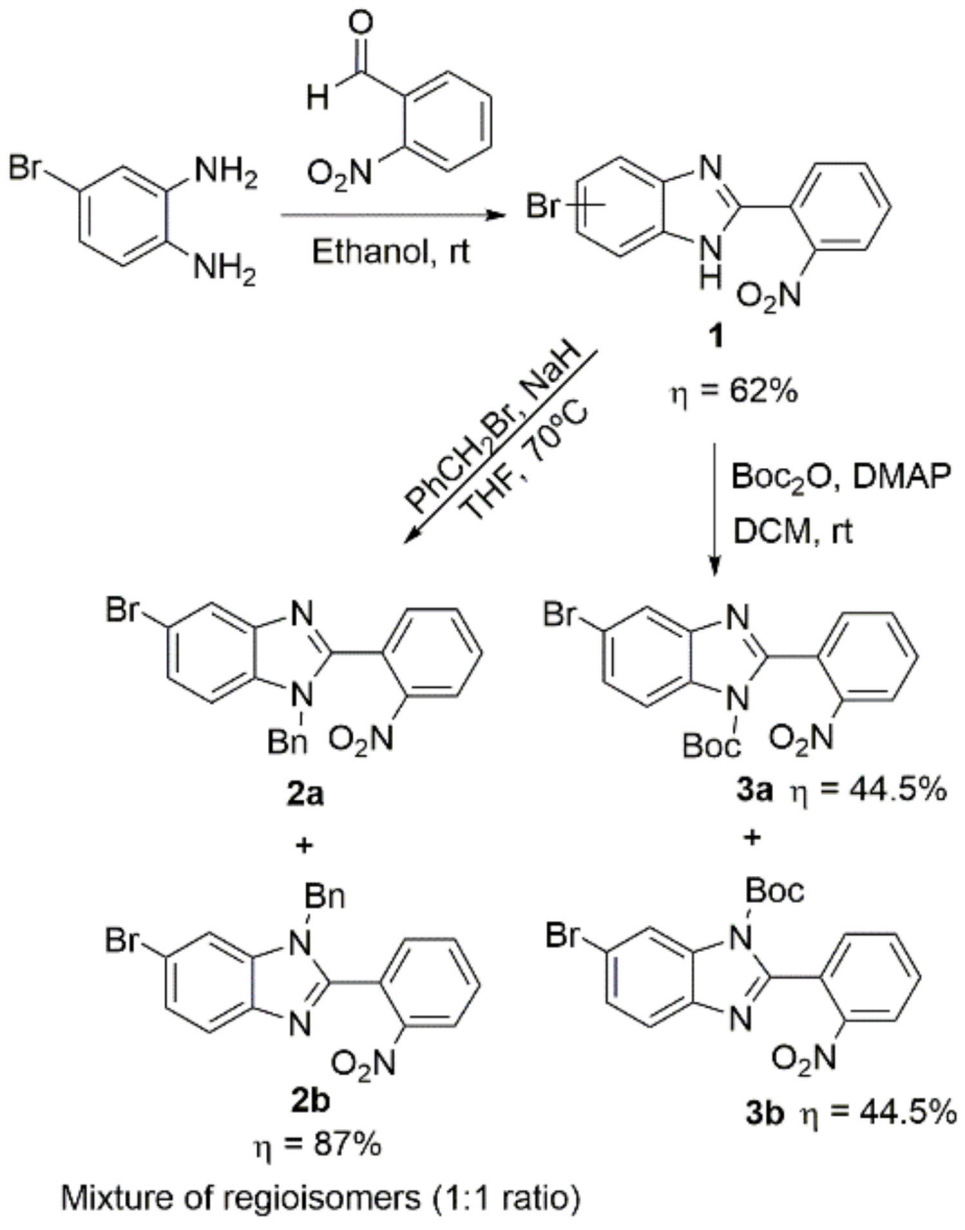
Synthesis of the core 5(6)-bromo-2-(2-nitrophenyl)-1*H*-benzimidazole substrate.

A simple synthetic pathway was developed for modulation of the desired 5(6)-position of the benzimidazole ring, starting with a set of reagents with an embedded functional group that could then be transformed to yield chemically diverse derivatives. Therefore, aiming toward the preparation of benzimidazoles [44] we started with 4-bromo-1,2-diaminobenzene and 2-nitrobenzaldehyde (Scheme 1).

The optimization of reaction conditions was performed for the synthesis of the nitro derivative **1**. The conditions explored and the results obtained are depicted in Table 2. Firstly, the reaction was carried out by mixing approximately equimolar quantities of 4-bromo-1,2-diaminobenzene and 2-nitrobenzaldehyde, using nitrobenzene both as solvent and oxidant, and the temperature was maintained at 180 °C for 8 h (Table 2, entry 1).

Since the product could not be isolated by precipitation from the reaction mixture, the nitrobenzene was distilled at reduced pressure. The product was then purified by column chromatography, using silica gel as stationary phase, and a mixture of dichloromethane/ethyl acetate as eluent. Under these conditions, product **1** was obtained at 48% isolated yield. ^13^C and ^1^H nuclear magnetic resonance showed broad signals (118.1, 116.5, 114.7 ppm), typical of tautomerism associated with this class of compounds (see Supplementary Materials: Figures S5 and S6). Then, in order to avoid this troublesome distillation step, nitrobenzene was replaced by ethanol and the reaction was carried out both at reflux temperature (80 °C) and room temperature (25 °C) in air atmosphere, over 3 h (Table 2, entry 2–3). This methodology gave similar yields for product **1** (47–50%), but a significantly easier work-up. Indeed, oxidation/aromatization could proceed using the atmospheric oxygen [45], without the need of using nitrobenzene, usually described as the oxidant [44,46]. To improve the yield via activation of carbonyl group, we evaluated the effect of Montmorillonite K10 as a heterogeneous acid reusable catalyst, and both the reproducibility of the reaction and the final isolated yield increased to 62% when the reaction was performed at 25 °C (Table 2, entries 4). Using this synthetic methodology, compound **1** was obtained at a multi-gram scale (2.64 g, 8.3 mmol) and was then used as a starting material for further modifying the 5(6)-position of the benzimidazole ring. Before starting the functionalization through Pd-catalyzed reactions, we proceeded with the benzimidazole –NH protection using benzyl or boc as protecting groups. In short, to a solution of the starting material **1**, in dry tetrahydrofuran (THF) at 0 °C and under inert atmosphere, NaH was added, followed by benzyl bromide and a catalytic amount of tetra-*n*-butylammonium iodide (TBAI). The reaction was then heated to 70 °C for 2 h. After conventional work-up procedures, product **2** (**2a** + **2b**) was isolated as a mixture of two regioisomers with 87% yield. As can be seen from the ^1^H NMR spectra (see Supplementary Materials: Figure S11), the isomers were formed in a 1:1 ratio, and were used as such in further reactions.

**Scheme 2:**
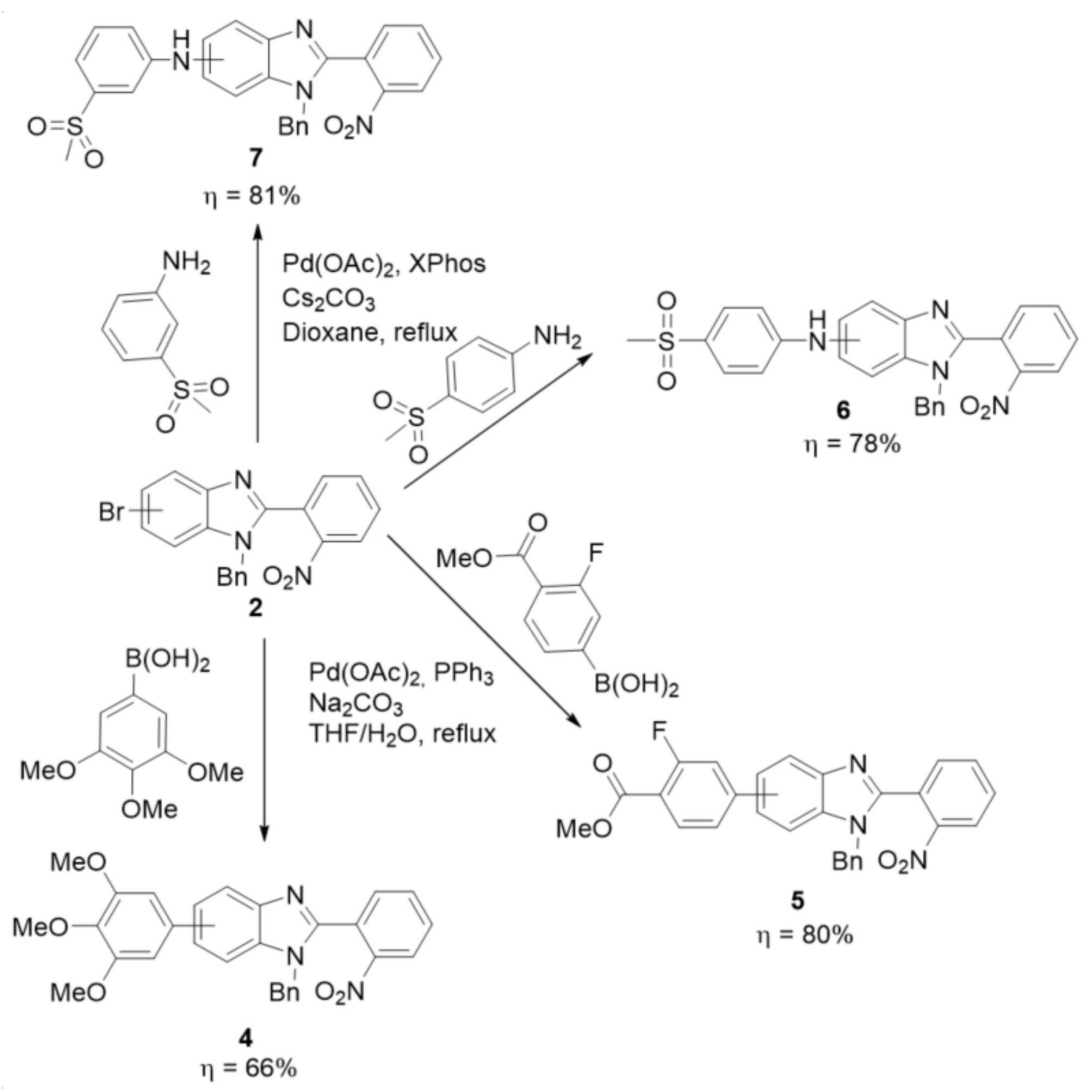
Pd-catalyzed reactions involving the *N*-benzyl-protected benzimidazole (**2**).

We proceeded with Pd-catalyzed reactions, using the mixture of *N*-benzyl-benzimidazole isomers, **2**, as substrate. Scheme 2 shows all the optimized reaction conditions and corresponding yields.

First, based on the computational model (mentioned above), we choose two boronic acids with hydrogen-bond acceptors present in different functional groups: (i) ether (3,4,5-trimethoxyphenylboronic acid); and (ii) ester and fluorine (3-fluoro-4-(methoxycarbonyl)phenylboronic acid).

In the first approach, through a Suzuki–Miyaura Pd-coupling reaction [47,48,49], the substrate **2** and the selected boronic acid were dissolved in a mixture of DME/EtOH 1:1, and the solvent was degassed. Then, Pd(OAc)_2_ and PPh_3_ were added and, after an incubation time of 15 min at room temperature, an aqueous solution of K_2_CO_3_ was added, after which the reaction was heated and maintained at 90 °C for 72 h. After the usual work-up procedure followed by purification by column silica gel chromatography, the compound **4** was isolated, as a mixture of isomers, in 30% yield. Aiming to improve the yield of this reaction, the solvent was changed to a mixture of THF/H_2_O (4:1). After being degassed, Pd(OAc)_2_, Ph_3_P, and K_2_CO_3_ were added, and the reaction was carried out at 70 °C for only 16 h. After a simple work-up, **4** was isolated in 66% yield. By applying these optimized reaction conditions, the formation of ester hydrolysis products was not observed and **5** was isolated in 80% yield.

Then, to prepare a family of molecules containing the sulfonylaniline group at 5(6)-benzimidazol position (the best scoring family according to the model: Table 1, entry 1), the optimization of Pd-catalyzed Buchwald–Hartwig amination [50] reaction was carried out and the results are presented in Table 3.

In a typical experiment, substrate **2** was mixed with 4-(methylsulfonyl)aniline, using Cs_2_CO_3_ as base and Pd(OAc)_2_/phosphine, and the reaction was carried out at 100 °C for 16 h. First, the reaction was performed in toluene, using Pd/BINAP, Pd/DPEphos, and Pd/XPhos and a remarkable effect of the phosphine structure was observed (Table 3, entries 1–3). The reaction could only be carried out in the presence of palladium/XPhos, yielding 91% conversion after 16 h (Table 3, entry 3). To improve the solubility of the reaction components and evaluate the solvent effect, toluene was then replaced by dioxane and the reaction was performed under the same conditions. Again, BINAP and DPEphos did not originate an active catalytic system (Table 3, entry 4–5), the palladium/XPhos being slightly more active in this solvent (100% conversion, Table 3, entry 6). Then, we reduced the reaction time from 16 h to 8 h, and 79% conversion was obtained (Table 3, entry 7). Finally, we increased the substrate/catalyst ratio from 10 to 20 and, after 16 h, only 57% substrate conversion was obtained (Table 3, entry 8). Overall, these results show that the catalytic activity is strongly dependent of the structure of palladium/phosphine catalyst, with the sterically hindered monodentate phosphine XPhos providing the most active catalytic system under these conditions, as previously reported [51].

Under our optimized conditions (Table 3, entry 6), and upon work-up procedure and purification by column chromatography in silica gel, the products **6** and **7** were isolated in yields of 78% and 81%, respectively (Scheme 2). Do, despite the lower nucleophilicity of the *para* derivative, when compared with the *meta* analogue, this factor did not translate into a noteworthy difference in reaction yield under the described conditions. To obtain the initially designed structures (Table 1), deprotection of the benzyl group was performed via catalytic hydrogenation using H_2_ and Pd/C [52], under mild conditions (50 °C, 3 bar H_2_) for 8 h. Nevertheless, after this time no benzyl deprotection occurred, and only the reduction of –NO_2_ was observed. Therefore, we used more vigorous reaction conditions (80 °C, 5 bar H_2_), but a complex mixture of products was obtained.

**Scheme 3:**
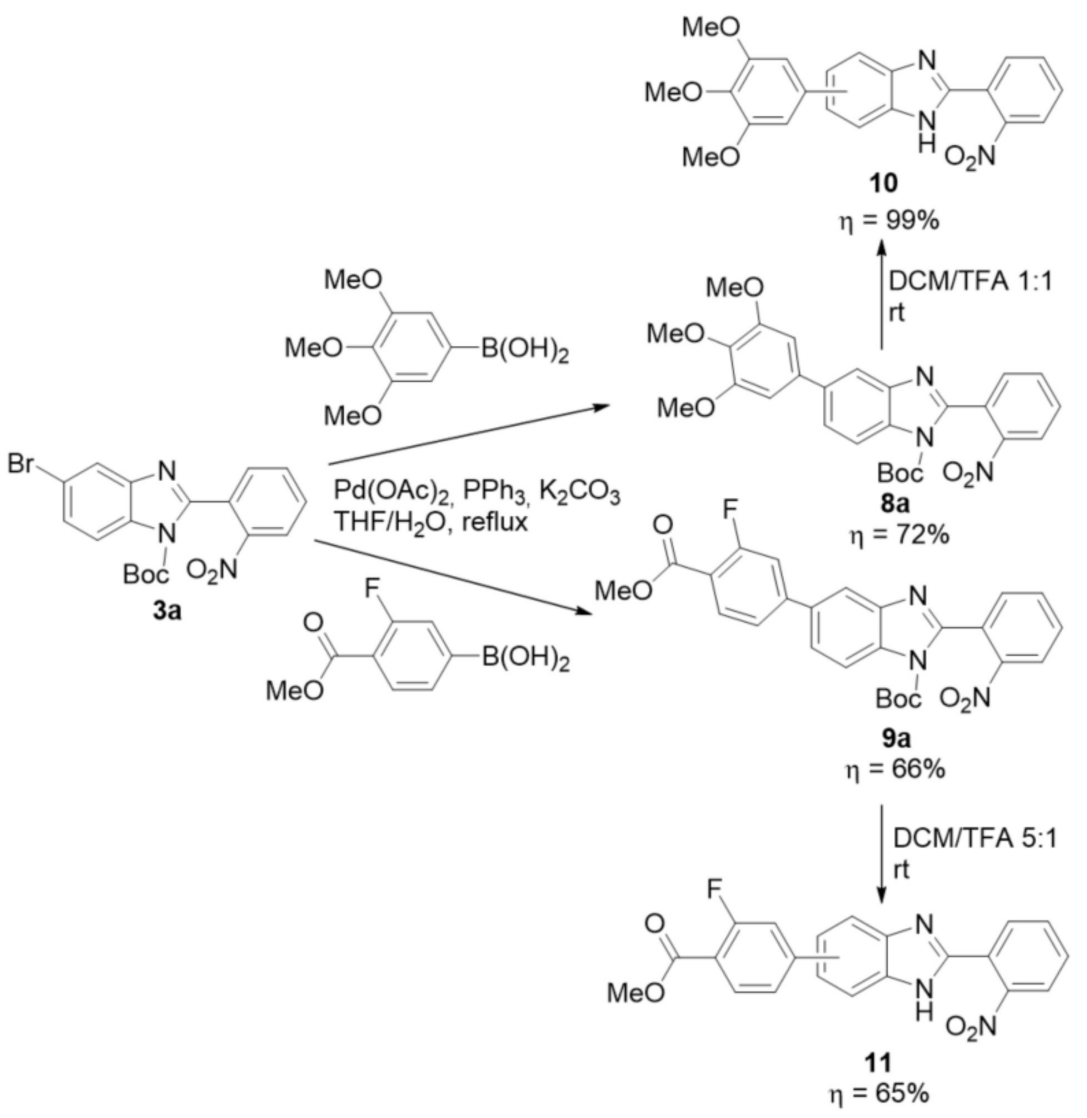
Suzuki coupling involving boc-protected benzimidazole (3a), and subsequent deprotection.

To overcome this synthetic challenge, we decided to protect the benzimidazole **1** with boc, yielding **3a** and **3b** (Scheme 1). The reaction was carried out in dichloromethane (DCM) for 24 h, at room temperature, with the addition of *tert*-butyldicarbonate and 4-dimethylaminopyridine (DMAP) as base. Following standard work-up, products **3a** and **3b** were obtained at 44.5% yield each (89% combined yield). It should be noted that, in this case, both isomers **3a** and **3b** could be easily separated by silica chromatography, being isolated and fully characterized. The assignment of each regioisomer was done using 2D-NOESY (see Supplementary Materials: Figures S18 and S23). Then, using the reaction conditions described above, **3a** or **3b** were coupled with 3,4,5-trimethoxyphenyl boronic acid or 3-fluoro-4-(methoxycarbonyl)phenyl boronic acid, giving products **8a** or **8b** and **9a** or **9b** with approximately the same NMR yields (75% and 70%, respectively). This result points to the fact that both regioisomers have similar reactivity towards Suzuki-Miyaura coupling. For further studies, isolation and full characterization, only the products **8a** and **9a** have been isolated upon column chromatography, in 72% and 66% isolated yields, respectively (Scheme 3). Full characterization is presented in the experimental section and SI.

For **8a**, deprotection was performed under the usual trifluoroacetic acid (TFA)/dichloromethane (DCM) 1:1 for 2 h, resulting in quantitative yield of product **10**. For **9a**, due to the presence of the methyl ester group, we decided to use TFA/DCM 1:5 (5 h) and, following purification, **11** was obtained in 65% yield.

**Scheme 4:**
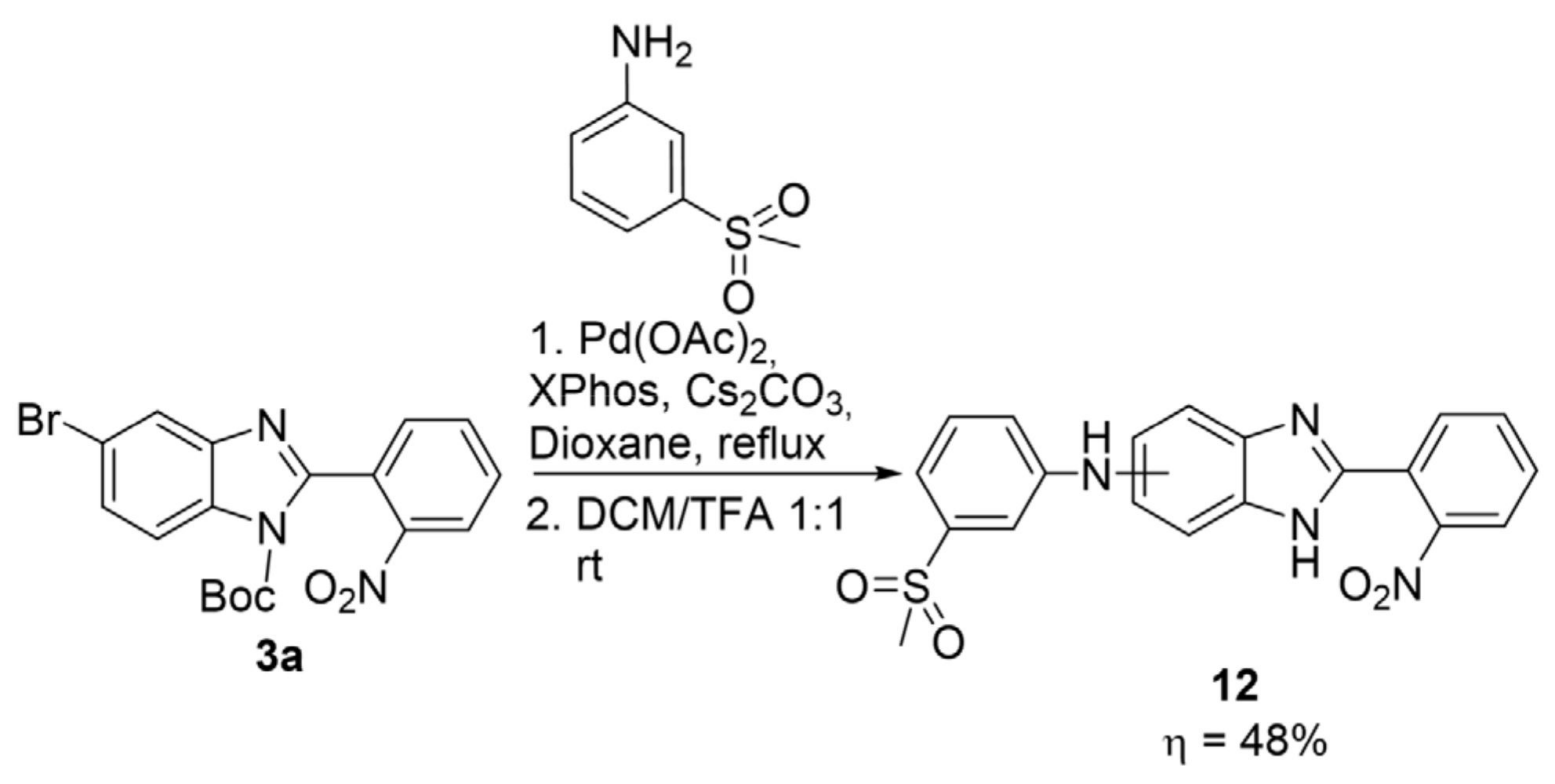
Buchwald–Hartwig coupling involving boc-protected benzimidazole (**3a**), and subsequent deprotection.

It should be noted that in the amination reaction of **3a** with 3-(methylsulfonyl)aniline (Scheme 4), using the reaction conditions described above, without isolation of the protected product, we obtained product **12** in 48% isolated yield. This slightly lower yield may be attributed to partial deprotection of the boc group during the reaction, since carbamates are much easier to cleave under basic conditions with less basic amines such as imidazole or benzimidazole [53]. Therefore, the boc-protected product was not isolated, and we proceeded directly to the deprotection step. When the reaction was finished, the solid was filtered, the solvent was evaporated and the solid was re-dissolved in a mixture of TFA/DCM 1:1 for 2 h. After basic work-up to neutralize the acid, product **12** was purified by column chromatography, and isolated in 48% yield.

**Scheme 5:**
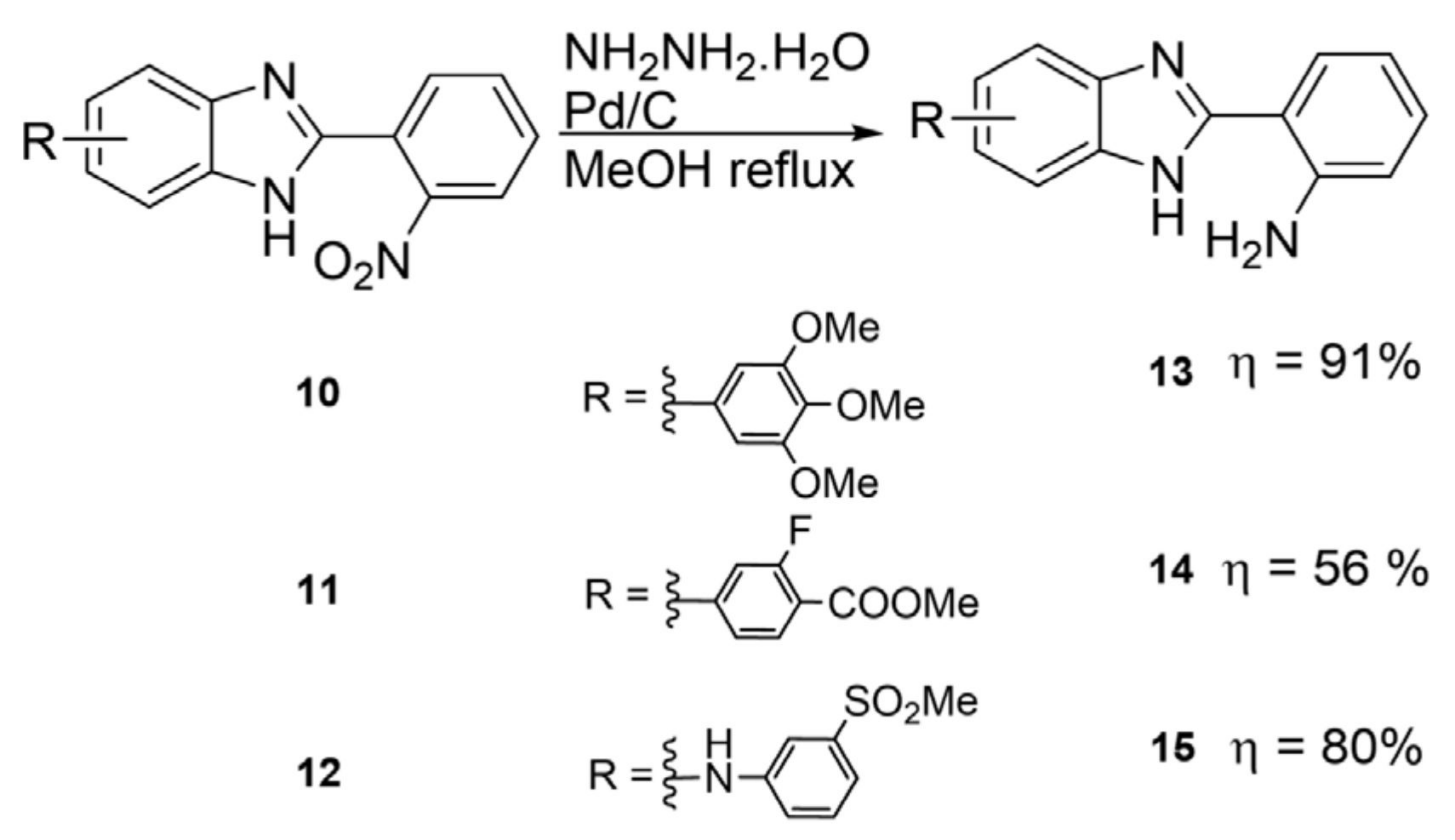
Pd/C catalyzed reduction of compounds **10**–**12** using hydrazine monohydrat.

Finally, to prepare our initially designed compounds (Table 1) with all the appropriate functional groups, according to the pharmacophore model developed, a catalytic hydrogenation of the nitrophenyl group was the final step of the synthetic route (Scheme 5). Briefly, the substrate, NH_2_NH_2_.H_2_O, and Pd/C were mixed in MeOH. The reaction was conducted at reflux temperature for 10–30 min, then filtered, and the solvent was evaporated, to give **13** in 91% yield, **14** in 56% yield (contaminated with dimeric amide in ~25%, see Supplementary Materials: Figure S55), and **15** in 80% yield. These new chemical entities show a good match to our initially proposed computational model (ChemScore of 33.7, 28.7 and 27.5 for compounds **15**, **14**, and **13**, respectively).

## 3. Materials and Methods

### 3.1. Generation of the Pharmacophore Model

Through a comprehensive literature search [9,10,35,36,37,38,39,40,41], several ligands of the *E. coli* DNA gyrase subunit B with different affinities were identified. From a total of 145 compounds, 61 were classified as actives (IC_50_ ≤ 1.0 µM), 54 as intermediates (1.0 µM < IC_50_ < 100 µM) and 30 as inactives (IC_50_ ≥ 100 µM). From the actives dataset, the most structurally diverse 18 compounds were selected as the training set while the remaining 43 were included in the test set. In cases where multiple derivatives were present, the compound with the highest activity was chosen. For the purpose of generating the pharmacophore queries, Molecular Operating Environment (MOE) 2018.0802 software [11] was used, using the Unified annotation scheme. After the training set ligands’ structural alignment, common features were identified using MOE’s Unified annotation scheme. From a set of about 50 pharmacophoric queries generated through variation of feature type, number, and radii, the best model was selected based on their performance in discriminating between actives and inactives in the test sets.

### 3.2. Docking Studies on E. coli DNA GyrB

Docking studies were performed in GOLD 5.4 (Cambridge Crystallographic Data Centre, Cambridge, UK) [43], using *E. coli*’s DNA Gyrase subunit B ATPase binding domain crystallographic structure (PDB entry 4KFG) [42]. For protein preparation, hydrogen atoms were added to the binding site residues and correct tautomers and protonation states were assigned. Water molecules and the ligand were deleted from the crystal structure before the docking studies. The binding site region was defined as the amino acid residues within a radius of 15 Å around the THR165 residue. To validate our protocol, the crystal structure ligand was docked into the defined binding site and the best scoring pose was able to reproduce the crystallographic pose with a root-mean-square deviation (RMSD) value of 0.57 Å. A set of 10 structurally diverse compounds with varying values of IC_50_ was docked using the defined protocol. The following scoring functions were tested: ChemScore, GOLDscore, and ChemPLP. The scoring function that yielded a better docking score correlation with experimental IC_50_ (ChemScore) was selected. From a set containing 6681 computationally generated derivatives of 2-aminophenyl-5(6)-substituted benzimidazoles, GOLD’s ChemScore function was used to rank their predicted inhibitory activity. The best scoring poses were then graphically analyzed in MOE and the relevant protein side-chain interactions were determined.

### 3.3. General Synthetic Methods

All reagents were purchased from commercial sources and used without further purification. Non-deuterated solvents (dichloromethane, ethyl acetate, hexane, 1,4-dioxane, ethanol, THF, methanol) and deuterated solvents (CDCl_3_, CD_3_OD, DMSO-*d*_6_ or acetone-*d*_6_) were purchased from Sigma-Aldrich (Lisbon, Portugal). Benzyl bromide, Montmorillonite K10, Cs_2_CO_3_, Pd(OAc)_2_, PPh_3_, Sodium, benzophenone, DMAP, tetra-*n*-butylammonium iodide, di-tert-butyl dicarbonate and silica gel powder/plates were also purchased from Sigma-Aldrich (Lisbon, Portugal). CaCl_2_, NaH, 3,4,5-trimethoxyphenylboronic acid, K_2_CO_3_, Na_2_SO_4_, NaHCO_3_, 3-fluoro-4-(methoxycarbonyl)phenylboronic acid, 4-(methylsulfonyl)aniline, 4-bromo-1,2-benzenediamine, Pd/C 5% were purchased from Fluorochem (Derbyshire, United Kingdom). 2-nitrobenzaldehyde was purchased from Alfa Aesar (Lancashire, United Kingdom). 3-(methylsulfonyl)aniline was purchased from Apollo Scientific (Cheshire, United Kingdom). ^1^H NMR and ^13^C NMR spectra were recorded on a Bruker Avance 400 spectrometer (Chemistry Department, University of Coimbra, Portugal) at 400 and 101 MHz, respectively. ^1^H and ^13^C NMR spectra were acquired in CDCl_3_, CD_3_OD, DMSO-*d*_6_ or acetone-*d*_6_, and the signals were referenced using the solvent peak or tetramethylsilane as an internal standard. High-resolution mass spectrometry (HRMS) analyses were conducted on a Bruker Microtof (Unidade de Espectrometría de Masas e Proteómica, Universidade de Santiago de Compostela, Spain). NMR and HRMS spectra are presented for all compounds in SI. All the reactions were monitored by thin-layer chromatography (TLC) using GF254 silica gel-coated TLC plates. Dichloromethane, ethyl acetate and hexane were distilled over anhydrous CaCl_2_ prior to use. 1,4-dioxane was dried using Na/benzophenone and stored over 4Å molecular sieves. For the Pd-catalyzed coupling reactions, all solvents were subjected to at least three cycles of the freeze-pump-thaw degassing method prior to the reaction, and the reactions were performed under inert atmosphere.

#### Procedures for the Synthesis of Products

**5(6)-bromo-2-(2-nitrophenyl)-1*H*-benzimidazole (1):**

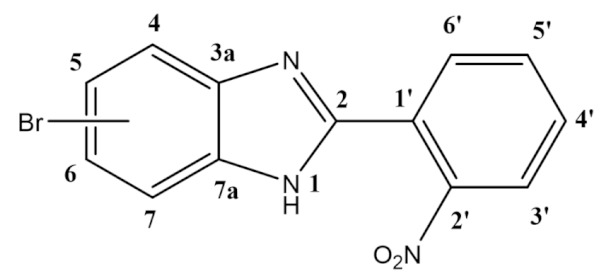

In a round-bottom flask, 4-bromo-1,2-benzenediamine (2.5 g; 13.4 mmol), 2-nitrobenzaldehyde (2.23 g; 14.8 mmol), and 250 mg of Montmorillonite K10 were mixed in ethanol (30 mL). The reaction was stirred at room temperature for 4 h. The mixture was filtered, the solvent was evaporated, and the dark orange slurry obtained was dissolved in ethyl acetate, followed by the addition of silica powder and the evaporation of the solvent to dryness. The resulting solid was loaded in a silica column, and the crude was purified dichloromethane/ethyl acetate 10:1 (R_f_ = 0.33). The product was obtained as a yellow solid in 62% yield (2.64 g). Characterization data in accordance to literature [45]. ^1^H NMR (400 MHz, DMSO-*d*_6_) ppm: 14.50–12.00 (brs, 1H_1_), 8.05 (dd, *J* = 8.0, 1.3 Hz, 1H_3′_), 7.97 (dd, *J* = 7.8, 1.5 Hz, 1H_6′_), 7.88 (td, *J* = 7.6, 1.3 Hz, 1H_5′_), 7.82 (d, *J* = 1.9 Hz, 1H_4/7_), 7.78 (td, *J* = 7.8, 1.5 Hz, 1H_4′_), 7.59 (d, *J* = 8.5 Hz, 1H_7/4_), 7.39 (dd, *J* = 8.5, 2.0 Hz, 1H_6/5_); ^13^C NMR (101 MHz, DMSO-*d*_6_) ppm: 148.9 (C2′), 148.7 (C2), 132.8 (C5′), 131.2 (C4′), 131.1 (C6′), 125.4 (C6/5), 124.4 (C3′), 123.9 (C1′), 118.1 (C4/7), 116.5 (C7/4), 114.7 (C5/6); HRMS (ESI-TOF) calcd. for C_13_H_9_BrN_3_O_2_: [M + H]^+^: 317.9878, found 317.9877.

**1-benzyl-5(6)-bromo-2-(2-nitrophenyl)-1*H*-benzimidazole (2):** In an oven-dried *Schlenk* tube coupled with a condenser, 5-bromo-2-(2-nitrophenyl)-1*H*-benzimidazole (2.5 g, 7.86 mmol) was dissolved in dry THF (5 mL). The system was degassed with 5x vacuum/argon cycles. To the stirring solution at 0 °C, NaH (60% in mineral oil) (226 mg, 9.43 mmol) was slowly added under an argon flow. After the evolution of the hydrogen gas, benzyl bromide (1.12 mL, 9.43 mmol) and a catalytic amount of tetra-*n*-butylammonium iodide were added. The mixture was heated at reflux temperature (70 °C) for 2 h. After cooling, the reaction was quenched with methanol, and the solvent was evaporated. The crude was purified by column chromatography using dichloromethane/ethyl acetate 1:20 (R_f_ = 0.40). The isomer mixture was obtained as a yellow solid in 87% yield (2.8 g). ^1^H NMR (400 MHz, CDCl_3_) (1:1 mixture of regioisomers) ppm: 8.17–8.10 (m, 1H_Ar_), 7.89 (d, *J* = 1.8 Hz, 0.5H_Ar_), 7.65–7.59 (m, 2.5H_Ar_), 7.44–7.33 (m, 2H_Ar_), 7.30 (dd, *J* = 8.6, 1.7 Hz, 0.5H_Ar_), 7.22–7.16 (m, 3H_Ar_), 7.03 (d, *J* = 1.8 Hz, 0.5H_Ar_), 6.96–6.90 (m, 2H_Ar_), 5.03 (m, 2H_benzyl_); ^13^C NMR (101 MHz, CDCl_3_) (1:1 mixture of regioisomers) ppm: 150.9, 150.5, 148.8, 144.1, 141.8, 136.2, 135.1, 135.0, 134.1, 133.6, 133.5, 132.73, 132.70, 131.58, 131.56, 129.2, 129.1, 128.41, 128.38, 126.8, 126.76, 126.72, 126.4, 125.38, 125.4, 125.2, 125.1, 123.1, 121.5, 117.0, 116.0, 113.8, 112.1, 48.7, 48.6; HRMS (ESI-TOF) calcd. for C_20_H_15_BrN_3_O_2_: [M + H]^+^: 408.0348, found 408.0345.

**General procedure for synthesis, isolation and characterization of 1-boc-5-bromo-2-(2-nitrophenyl)-benzimidazole (3a) and 1-boc-6-bromo-2-(2-nitrophenyl)-benzimidazole (3b):** In a round-bottom flask, 5-bromo-2-(2-nitrophenyl)-1*H*-benzimidazole (1.66 g, 5.22 mmol), di-tert-butyl dicarbonate (2.28 g, 10.44 mmol) and DMAP (638 mg, 5.22 mmol) were mixed in dichloromethane (30 mL), and the reaction was left stirring at room temperature for 24 h. The solvent was evaporated, and the product was purified by column chromatography using hexane/ethyl acetate 4:1 as eluent (R_f_ = 0.40 for **3a** R_f_ = 0.27 for **3b**). Two pure fractions of light yellow solids (isomers **3a** and **3b**) were obtained in a 1:1 ratio with an overall yield of 89% (1.95 g).

**1-boc-5-bromo-2-(2-nitrophenyl)-benzimidazole (3a)**:

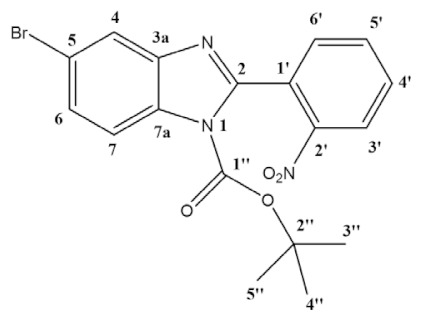

^1^H NMR (400 MHz, CDCl_3_) ppm: 8.30 (dd, *J* = 8.2, 1.3 Hz, 1H_3′_), 7.95 (d, *J* = 8.7 Hz, 1H_7_), 7.92 (d, *J* = 1.9 Hz, 1H_4_), 7.79 (td, *J* = 7.5, 1.3 Hz, 1H_5′_), 7.70 (td, *J* = 7.9, 1.5 Hz, 1H_4′_), 7.64 (dd, *J* = 7.5, 1.6 Hz, 1H_6′_), 7.54 (dd, *J* = 8.7, 1.9 Hz, 1H_6_), 1.37 (s, 9H_3″–5″_). ^13^C NMR (101 MHz, CDCl_3_) ppm: 151.0 (C2), 147.8 (C1″), 147.7 (C2′), 144.0 (C3a), 133.9 (C5′), 132.2 (C7a), 132.1 (C6′), 130.8 (C4′), 128.8 (C1′), 128.7 (C6), 124.7 (C3′), 123.4 (C4), 117.7 (C5), 116.7 (C7), 86.4 (C2″), 27.7 (C3″,C4″,C5″); Assignment of regioisomer was done using 2D-NMR, see Supplementary Materials: Figure S18;

**1-boc-6-bromo-2-(2-nitrophenyl)-benzimidazole (3b)**:

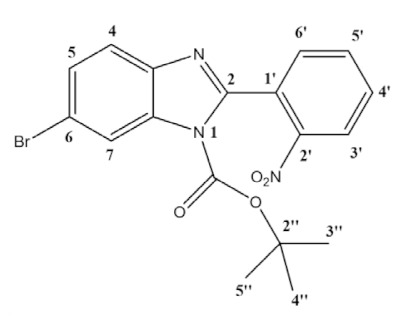

^1^H NMR (400 MHz, CDCl_3_) ppm: 8.30 (dd, *J* = 8.2, 1.3 Hz, 1H_3′_), 8.28 (d, *J* = 1.9 Hz, 1H_7_), 7.79 (td, *J* = 7.5, 1.3 Hz, 1H_5′_), 7.70 (td, *J* = 7.9, 1.5 Hz, 1H_4′_), 7.65 (dd, *J* = 7.5, 1.5 Hz, 1H_6′_), 7.64 (d, *J* = 8.5, 1H_4_), 7.52 (dd, *J* = 8.5, 1.9 Hz, 1H_5_), 1.37 (s, 9H_3″–5″_). ^13^C NMR (101 MHz, CDCl_3_) ppm: 150.2, 147.6, 147.6, 141.6, 133.9, 133.8, 132.0, 130.7, 128.7, 127.9, 124.6, 121.4, 119.0, 118.5, 86.4, 27.5; HRMS (ESI-TOF) calcd. for C_18_H_17_BrN_3_O_4_: [M + H]^+^: 418.0402, found 418.0403. Assignment of regioisomer was done using 2D-NMR, see Supplementary Materials: Figure S23.

**1-benzyl-5(6)-(3,4,5-trimethoxyphenyl)-2-(2-nitrophenyl)-benzimidazole (4):** In a *Schlenk* tube, 1-benzyl-5(6)-bromo-2-(2-nitrophenyl)-benzimidazole (500 mg, 1.22 mmol) and 3,4,5-trimethoxyphenylboronic acid (526 mg, 2.48 mmol) were dissolved in a mixture of THF/H_2_O 4:1 (15 mL). Under an argon flow, Pd(OAc)_2_ (27 mg; 0.12 mmol), PPh_3_ (80 mg; 0.30 mmol) and K_2_CO_3_ (380 mg, 9.2 mmol) were added and the reaction was heated to 70 °C for 16 h. After cooling, ethyl acetate was added to the reaction, and the insoluble solid was filtered. The organic phase was then washed with a 1 M solution of NaOH (5x), followed by water (2x). After drying with Na_2_SO_4_ and filtering, the solvent was evaporated. Then, a column chromatography in silica gel was performed using dichloromethane/ethyl acetate 5:1 as eluent (R_f_ = 0.38). The isomer mixture was obtained as a yellow solid in 66% yield (399 mg). ^1^H NMR (400 MHz, CDCl_3_) (1:1 mixture of regioisomers) ppm: 8.20–8.11 (m, 1H_Ar_), 7.94 (d, *J* = 1.6 Hz, 0.5H_Ar_), 7.79 (d, *J* = 8.4 Hz, 0.5H_Ar_), 7.67–7.58 (m, 2H_Ar_), 7.48–7.40 (m, 2H_Ar_), 7.27–7.18 (m, 4H_Ar_), 7.02–6.95 (m, 2H_Ar_), 6.76 (s, 1H_Ar_), 6.63 (s, 1H_Ar_), 5.19 (s, 1H_benzyl_), 5.17 (s, 1H_benzyl_), 3.87–3.80 (m, 9H_OMe_); ^13^C NMR (101 MHz, CDCl_3_) (1:1 mixture of regioisomers) ppm: 153.57, 153.55, 150.4, 150.3, 148.9, 137.71, 137.67, 137.5, 137.1, 135.6, 135.4, 135.3, 134.5, 133.54, 133.51, 132.83, 132.77, 131.54, 131.46, 129.12, 129.08, 128.3, 128.2, 126.9, 126.8, 125.7, 125.2, 125.1, 123.6, 122.9, 120.2, 118.4, 111.0, 109.3, 105.0, 104.9, 61.1, 56.3, 48.7; HRMS (ESI-TOF) calcd. for C_29_H_26_N_3_O_5_: [M + H]^+^: 496.1872, found 496.1870.

**1-benzyl-5(6)-(3-fluoro-4-(methoxycarbonyl)phenyl)-2-(2-nitrophenyl)-benzimidazole (5):** In a *Schlenk* tube, 1-benzyl-5(6)-bromo-2-(2-nitrophenyl)-benzimidazole (300 mg, 0.74 mmol) and 3-fluoro-4-(methoxycarbonyl)phenylboronic acid (293 mg, 1.48 mmol) were dissolved in a mixture of THF/H_2_O 4:1 (8 mL). Under an argon flow, Pd(OAc)_2_ (16 mg; 0.074 mmol), PPh_3_ (48 mg; 0.18 mmol), and K_2_CO_3_ (100 mg, 1.38 mmol) were added and the reaction was heated to 70 °C for 16 h. After cooling, ethyl acetate was added to the reaction, and the insoluble solid was filtered. The organic phase was then washed with water (2x). After drying with Na_2_SO_4_ and filtering, the solvent was evaporated. Then, a column chromatography in silica gel was performed using dichloromethane/ethyl acetate 5:1 as eluent (R_f_ = 0.38). The isomer mixture was obtained as a yellow solid in 80% yield (286 mg). **^1^**H NMR (400 MHz, acetone-*d*_6_) (1:1 mixture of regioisomers) ppm: 8.28–8.20 (m, 1H_Ar_), 8.06 (d, *J* = 1.7 Hz, 0.5H_Ar_), 8.04–7.95 (m, 1H_Ar_), 7.95 (d, *J* = 1.7 Hz, 0.5H_Ar_), 7.90–7.83 (m, 2H_Ar_), 7.80 (d, *J* = 8.5 Hz, 0.5H_Ar_), 7.76–7.52 (m, 4.5H_Ar_), 7.33–7.23 (m, 3H_Ar_), 7.20–7.15 (m, 2H_Ar_), 5.28 (s, 1H_benzyl_), 5.25 (s, 1H_benzyl_), 3.94 (s, 1.5H_OMe_), 3.93 (s, 1.5H_OMe_); ^13^C NMR (101 MHz, acetone-*d*_6_) (1:1 mixture of regioisomers) ppm: 165.0, 164.9, 164.9, 164.9, 164.3, 164.2, 161.7, 161.6, 152.0, 151.9, 150.4, 150.3, 149.3, 149.2, 149.1, 149.0, 144.9, 144.9, 137.2, 137.1, 137.1, 136.9, 134.4, 134.3, 134.3, 134.2, 133.8, 133.8, 133.3, 133.2, 133.2, 132.3, 132.3, 129.6, 129.5, 128.6, 128.6, 127.9, 127.9, 126.5, 126.4, 125.8, 125.7, 123.6, 123.6, 123.4, 122.6, 121.2, 119.4, 117.6, 117.5, 117.5, 117.4, 116.1, 116.1, 115.9, 115.8, 112.5, 110.7, 52.5, 52.5, 49.0, 48.8; HRMS (ESI-TOF) calcd. for C_28_H_21_FN_3_O_4_: [M + H]^+^: 482.1516, found 482.1513.

**1-benzyl-*N*-(4-(methylsulfonyl)phenyl)-2-(2-nitrophenyl)-1-benzimidazol-5(6)-amine (6):** In a *Schlenk* tube, Pd(OAc)_2_ (0.074 mmol, 16.5 mg), XPhos (0.11 mmol, 53 mg), 1-benzyl-5(6)-bromo-2-(2-nitrophenyl)-benzimidazole (300 mg, 0.74 mmol), 4-(methylsulfonyl)aniline (151 mg, 0.88 mmol) and Cs_2_CO_3_ (1.47 mmol, 479 mg) were added to dry dioxane (5 mL) and the temperature was set to 100 °C. The reaction was carried out at reflux temperature for 16 h. After cooling, the crude mixture was filtered and the solid was washed with acetone. The filtrate was evaporated and a column chromatography in silica gel was performed using dichloromethane/ethyl acetate 1:1 as eluent (R_f_ = 0.29). After drying, a brown solid was obtained in 78% yield (282 mg). ^1^H NMR (400 MHz, acetone-*d*_6_): (1:1 mixture of regioisomers) ppm: 8.25–8.10 (m, 2H_Ar_), 7.90–7.80 (m, 2H_Ar_), 7.80–7.55 (m, 4H_Ar_), 7.42 (d, *J* = 8.6 Hz, 0.5H_Ar_), 7.36–7.16 (m, 6H_Ar_), 7.16–7.08 (m, 1.5H_Ar_), 7.04 (d, *J* = 8.9 Hz, 1H_Ar_), 5.46 (s, 1H_benzyl_), 5.44 (s, 1H_benzyl_), 3.01 (s, 3H_Me_). ^13^C NMR (101 MHz, acetone-*d*_6_) (1:1 mixture of regioisomers) ppm: 151.6, 151.3, 150.8, 150.6, 150.4, 145.1, 141.0, 137.6, 137.3, 136.9, 136.8, 134.21, 134.15, 133.4, 133.3, 133.2, 132.2, 132.1, 130.8, 130.4, 130.1, 130.0, 129.62, 129.57, 128.6, 127.91, 127.87, 126.60, 126.56, 125.73, 125.70, 121.4, 119.9, 118.4, 114.5, 114.2, 114.1, 113.7, 112.5, 104.1, 48.9, 44.94, 44.88; HRMS (ESI-TOF) calcd. for C_27_H_23_N_4_O_4_S: [M + H]^+^: 499.1440, found 499.1434.

**1-benzyl-*N*-(3-(methylsulfonyl)phenyl)-2-(2-nitrophenyl)-benzimidazol-5(6)-amine (7):** In a *Schlenk* tube, Pd(OAc)_2_ (0.074 mmol, 16.5 mg), XPhos (0.11 mmol, 53 mg), 1-benzyl-5(6)-bromo-2-(2-nitrophenyl)-benzimidazole (300 mg, 0.74 mmol), 3-(methylsulfonyl)aniline (151 mg, 0.88 mmol) and Cs_2_CO_3_ (1.47 mmol, 479 mg) were added to dry dioxane (5 mL) and the temperature was set to 100 °C. The reaction was carried out at reflux temperature for 16 h. After cooling, the crude mixture was filtered and the solid was washed with acetone. The filtrate was evaporated and a column chromatography in silica gel was performed using dichloromethane/ethyl acetate 1:1 as eluent (R_f_ = 0.23). After drying, a brown solid was obtained in 81% yield (300 mg). ^1^H NMR (400 MHz, acetone-*d*_6_) (1:1 mixture of regioisomers) ppm: 8.12 (s, 1H_Ar_), 7.77 (dd, *J* = 7.6, 1.7 Hz, 1H_Ar_), 7.45–7.36 (m, 2H_Ar_), 7.31 (dd, *J* = 7.3, 1.8 Hz, 1H_Ar_), 7.04–6.95 (m, 4H_Ar_), 6.85–6.75 (m, 5H_Ar_), 6.70–6.64 (m, 3H_Ar_), 4.95 (m, 2H_benzyl_), 2.70 (s, 3H_Me_); ^13^C NMR (101 MHz, acetone-*d*_6_) (1:1 mixture of regioisomers) ppm: 149.7, 149.0, 146.3, 143.5, 141.8, 136.9, 136.3, 133.6, 132.2, 131.6, 131.2, 130.3, 128.6, 127.7, 127.0, 124.94, 124.93, 118.7, 117.6, 116.0, 111.9, 111.6, 109.9, 47.6, 43.7; HRMS (ESI-TOF) calcd. for C_27_H_23_N_4_O_4_S: [M + H]^+^: 499.1440, found 499.1436.

**1-boc-5-(3,4,5-trimethoxyphenyl)-2-(2-nitrophenyl)-benzimidazole (8a):** In a *Schlenk* tube, 1-boc-5-bromo-2-(2-nitrophenyl)-benzimidazole (300 mg, 0.72 mmol) and 3,4,5-trimethoxyphenylboronic acid (229 mg, 1.08 mmol) were dissolved in a mixture of THF/H_2_O 4:1 (5 mL). Under an argon flow, Pd(OAc)_2_ (16 mg; 0.72 mmol), PPh_3_ (47 mg; 0.18 mmol), and K_2_CO_3_ (89 mg, 2.2 mmol) were added and the reaction was heated to 70 °C for 16 h. After cooling, ethyl acetate was added to the reaction, and the insoluble solid was filtered. The organic phase was then washed with a 1 M solution of NaOH (5x), followed by water (2x). After drying with Na_2_SO_4_ and filtering, the solvent was evaporated. Then, a column chromatography in silica gel was performed using a gradient mixture as eluent, starting with dichloromethane and ending in dichloromethane/ethyl acetate 20:1 (R_f_ = 0.50). The light yellow solid was obtained in 72% yield (260 mg). ^1^H NMR (400 MHz, acetone-*d*_6_) ppm: 8.37–8.34 (m, 1H_Ar_), 8.14 (d, *J* = 8.6 Hz, 1H_Ar_), 8.00–7.95 (m, 2H_Ar_), 7.91–7.85 (m, 2H_Ar_), 7.78 (dd, *J* = 8.6, 1.8 Hz, 1H_Ar_), 7.04 (s, 2H_Ar_), 3.96 (s, 6H_OMe_), 3.79 (s, 3H_OMe_), 1.39 (s, 9H_boc_); ^13^C NMR (101 MHz, acetone-*d*_6_) ppm: 154.9, 151.3, 148.93, 148.91, 144.6, 139.1, 139.0, 137.6, 135.0, 133.5, 133.3, 131.8, 130.1, 125.5, 125.4, 119.2, 116.2, 105.8, 86.5, 60.73, 60.65, 56.7, 27.7; HRMS (ESI-TOF) calcd. for C_27_H_28_N_3_O_7_: [M + H]^+^: 506.1927, found 506.1924.

**1-boc-5-(3-fluoro-4-(methoxycarbonyl)phenyl)-2-(2-nitrophenyl)-benzimidazole (9a):** In a *Schlenk* tube, 1-boc-5-bromo-2-(2-nitrophenyl)-benzimidazole (300 mg, 0.74 mmol) and 3-fluoro-4-(methoxycarbonyl)phenylboronic acid (170 mg, 0.86 mmol) were dissolved in a mixture of THF/H_2_O 4:1 (5 mL). Under an argon flow, Pd(OAc)_2_ (16 mg; 0.074 mmol), PPh_3_ (48 mg; 0.18 mmol), and K_2_CO_3_ (298 mg, 2.15 mmol) were added and the reaction was heated to 70 °C for 16 h. After cooling, ethyl acetate was added to the reaction, and the insoluble solid was filtered. The organic phase was then washed with water (2x). After drying with Na_2_SO_4_ and filtering, the solvent was evaporated. Then, a column chromatography in silica gel was performed using dichloromethane as eluent (R_f_ = 0.36). The product was obtained as a yellow solid in 66% yield (235 mg). ^1^H NMR (400 MHz, acetone-*d*_6_) ppm: 8.32 (d, *J* = 1.8 Hz, 1H_Ar_), 8.24 (d, *J* = 8.6 Hz, 1H_Ar_), 7.94 (t, *J* = 7.9 Hz, 1H_Ar_), 7.86 (td, *J* = 7.5, 1.3 Hz, 1H_Ar_), 7.80–7.66 (m, 4H_Ar_), 7.59 (dd, *J* = 8.2, 1.8 Hz, 1H_Ar_), 7.52 (dd, *J* = 12.3, 1.8 Hz, 1H_Ar_), 3.80 (s, 6H_OMe_), 1.26 (s, 9H_boc_). ^13^C NMR (101 MHz, acetone-*d*_6_) ppm: 165.0 (d, *J* = 3.7 Hz), 163.1 (d, *J* = 258.1 Hz), 152.0, 148.87 (d, *J* = 8.8 Hz), 148.84, 148.79, 144.5, 136.8 (d, *J* = 2.0 Hz), 135.1, 134.8, 133.7 (d, *J* = 1.7 Hz), 133.3, 131.9, 129.9, 125.4, 124.8, 123.9 (d, *J* = 3.4 Hz), 121.6, 118.2 (d, *J* = 10.6 Hz), 116.4 (d, *J* = 23.6 Hz), 115.0, 86.8, 52.6, 27.7; HRMS (ESI-TOF) calcd. for C_26_H_23_FN_3_O_6_: [M + H]^+^: 492.1571, found 492.1564.

**5(6)-(3,4,5-trimethoxyphenyl)-2-(2-nitrophenyl)-1*H*-benzimidazole (10):** In a round-bottom flask, 1-boc-5-(3,4,5-trimethoxyphenyl)-2-(2-nitrophenyl)-1*H*-benzimidazole (0.28 mmol, 140 mg) was dissolved in a mixture of DCM/TFA 1:1 (2.0 mL). After 3 h, the acid was neutralized by a slow addition of a saturated aqueous solution of NaHCO_3_. Dichloromethane was added and the organic phase was washed 2x with a saturated solution of NaHCO_3_ and then 2x with H_2_O, followed by drying anhydrous Na_2_SO_4_, filtering and solvent evaporation. The product was purified by column chromatography in silica gel using DCM/Ethyl acetate 1:2 as eluent (R_f_ = 0.37). After drying, a yellow solid was obtained in 99% yield (112 mg). ^1^H NMR (400 MHz, DMSO-*d*_6_) ppm: 13.09 (brs, 1H_NH_), 8.04 (dd, *J* = 8.1, 1.2 Hz, 1H_Ar_), 8.00 (dd, *J* = 7.8, 1.4 Hz, 1H_Ar_), 7.91–7.87 (m, 2H_Ar_), 7.77 (td, *J* = 7.8, 1.4 Hz, 1H_Ar_), 7.67 (d, *J* = 8.4 Hz, 1H_Ar_), 7.59 (dd, *J* = 8.5, 1.7 Hz, 1H_Ar_), 6.97 (s, 2H_Ar_), 3.89 (s, 6H_OMe_), 3.70 (s, 3H_boc_); ^13^C NMR (101 MHz, DMSO-*d*_6_) ppm: 153.2, 149.0, 148.0, 136.80, 136.78, 135.3, 132.7, 131.0, 130.9, 124.3, 124.1, 122.2, 104.4, 60.1, 56.0; HRMS (ESI-TOF) calcd. for C_22_H_20_N_3_O_5_: [M + H]^+^: 406.1403, found 406.1404.

**5(6)-(3-fluoro-4-(methoxycarbonyl)phenyl)-2-(2-nitrophenyl)-1*H*-benzimidazole (11):** In a round-bottom flask, 1-boc-5-(3-fluoro-4-(methoxycarbonyl)phenyl)-2-(2-nitrophenyl)-benzimidazole (0.42 mmol, 208 mg) was dissolved in DCM (2.0 mL) and TFA (0.4 mL) was added. After 5 h, the acid was neutralized by a slow addition of a saturated aqueous solution of NaHCO_3_. Dicloromethane was added and the organic phase was washed 2x with a saturated solution of NaHCO_3_ and then 2x with H_2_O, followed by drying anhydrous Na_2_SO_4_, filtering and solvent evaporation. The product was purified by column chromatography in silica gel using DCM/Ethyl acetate 6:1 as eluent (R_f_ = 0.27). After drying, a yellow solid was obtained in 65% yield (107 mg). ^1^H NMR (400 MHz, acetone-*d*_6_) ppm: 12.20–12.15 (m, 1H_NH_), 8.09–7.93 (m, 4H_Ar_), 7.88 (td, *J* = 7.6, 1.4 Hz, 1H_Ar_), 7.83–7.59 (m, 5H_Ar_), 3.92 (s, 3H_OMe_); ^13^C NMR (101 MHz, acetone-*d*_6_) ppm: 165.0 (d, *J* = 3.8 Hz), 163.0 (d, *J* = 258.1 Hz), 150.5, 149.9, 149.3, 133.5, 133.4 (d, *J* = 1.7 Hz), 132.1, 131.8, 125.8, 125.3, 123.6 (d, *J* = 3.2 Hz), 117.4, 116.0 (d, *J* = 23.4 Hz), 52.5; HRMS (ESI-TOF) calcd. for C_21_H_15_FN_3_O_4_: [M + H]^+^: 392.1047, found 392.1042.

***N*-(3-(methylsulfonyl)phenyl)-2-(2-nitrophenyl)-1*H*-benzimidazol-5(6)-amine (12):** In a *Schlenk* tube, Pd(OAc)_2_ (0.072 mmol, 16.1 mg), XPhos (0.11 mmol, 52 mg), 1-benzyl-5(6)-bromo-2-(2-nitrophenyl)-benzimidazole (300 mg, 0.72 mmol), 3-(methylsulfonyl)aniline (147 mg, 0.86 mmol) and Cs_2_CO_3_ (1.43 mmol, 467 mg) were added to dry dioxane (5 mL) and the temperature was set to 100 °C. The reaction was carried out at reflux temperature for 16 h. After cooling, the crude mixture was filtered and the solid was washed with acetone. The filtrate was evaporated and dissolved in a mixture of DCM/TFA 1:1 (4.0 mL). After 2 h, the acid was neutralized by a slow addition of a saturated aqueous solution of NaHCO_3_. Ethyl acetate was added to the mixture, and the organic phase was washed 2x with a saturated solution of NaHCO_3_ and then 2x with H_2_O. The organic phase was dried with anhydrous Na_2_SO_4_, filtered, and evaporated. A purification by column chromatography in silica gel was performed using dichloromethane/ethyl acetate 1:1 as eluent (R_f_ = 0.38). After drying, a brown solid was obtained in 48% yield (140 mg). **^1^**H NMR (400 MHz, acetone-*d*_6_) ppm: 11.95 (brs, 1H_NH_), 8.02 (dd, *J* = 7.7, 1.5 Hz, 1H_Ar_), 7.98 (dd, *J* = 8.1, 1.3 Hz, 1H_Ar_), 7.90–7.80 (m, 2H_Ar_), 7.74 (m, 1H_Ar_), 7.65–7.55 (m, 2H_Ar_), 7.47 (s, 1H_Ar_), 7.44 (d, *J* = 7.9 Hz, 1H_Ar_), 7.35 (d, *J* = 8.2 Hz, 1H_Ar_), 7.31 (d, *J* = 7.7 Hz, 1H_Ar_), 7.16 (d, *J* = 8.6 Hz, 1H_Ar_), 3.08 (s, 3H_Me_); ^13^C NMR (101 MHz, acetone-*d*_6_) ppm: 150.4, 143.4, 133.3, 131.8, 131.4, 131.1, 125.9, 125.2, 117.8, 113.7, 44.3; HRMS (ESI-TOF) calcd. for C_20_H_17_N_4_O_4_S: [M + H]^+^: 409.0971, found 409.0964.

**2-(2-aminophenyl)-5(6)-(3,4,5-trimethoxyphenyl)-1*H*-benzimidazole (13):** In a round-bottom flask, 5(6)-(3,4,5-trimethoxyphenyl)-2-(2-nitrophenyl)-1*H*-benzimidazole (50 mg, 0.125 mmol), hydrazine monohydrate (45 μL, 1.25 mmol) and Pd/C 5% (10 mg) were mixed in methanol (1.0 mL). The solution was stirred for 30 min at reflux temperature (70 °C). The resulting solution was filtrated and the solvent evaporated. A white solid was obtained in 91% yield (43 mg). **^1^**H NMR (400 MHz, CD_3_OD) ppm: 7.77 (brs, 1H_Ar_), 7.72 (dd, *J* = 7.8, 1.3 Hz, 1H_Ar_), 7.61 (brs, 1H_Ar_), 7.48 (dd, *J* = 8.3, 1.7 Hz, 1H_Ar_), 7.19 (ddd, *J* = 8.4, 7.2, 1.5 Hz, 1H_Ar_), 6.88 (dd, *J* = 8.2, 1.2 Hz, 1H_Ar_), 6.75 (ddd, *J* = 8.2, 7.1, 1.2 Hz, 1H_Ar_), 3.92 (s, 6H_OMe_), 3.81 (s, 3H_OMe_); ^13^C NMR (101 MHz, CD_3_OD) ppm: 154.8, 154.7, 149.1, 139.7, 138.4, 131.8, 128.6, 123.1, 117.9, 117.7, 113.2, 105.8, 61.2, 56.7; HRMS (ESI-TOF) calcd. for C_22_H_22_N_3_O_3_: [M + H]^+^: 376.1661, found 376.1655.

**2-(2-aminophenyl)-5(6)-(3-fluoro-4-(methoxycarbonyl)phenyl)-1*H*-benzimidazole (14):** In a round-bottom flask, 5(6)-(3-fluoro-4-(methoxycarbonyl)phenyl)-2-(2-nitrophenyl)-1*H*-benzimidazole (20 mg, 0.051 mmol), hydrazine monohydrate (25 μL, 0.75 mmol) and Pd/C 5% (2 mg) were mixed in methanol (2.0 mL). The solution was stirred for 1 h at reflux temperature (70 °C). The product precipitated on the reaction mixture, and thus the crude was filtrated and the filtrate was dissolved in DMF. After evaporation of the solvent, a yellow solid was obtained in 56% yield (15.7 mg). **^1^**H NMR (400 MHz, DMSO-*d*_6_) ppm: 12.83 (brs, 1H_NH_), 8.13–7.54 (m, 7H_Ar_), 7.29 (brs, 2H_Ar_), 7.18 (t, *J* = 7.7 Hz, 1H_Ar_), 6.85 (d, *J* = 8.2 Hz, 1H_Ar_), 6.67 (t, *J* = 7.5, 1H_Ar_), 3.88 (s, 3H_OMe_); ^13^C NMR (101 MHz, DMSO-*d*_6_) ppm: 164.0, 161.8 (d, *J* = 295 Hz), 154.0 (d, *J* = 24 Hz), 148.4, 143.7, 134.3, 132.3, 130.7, 127.4, 122.6, 121.9, 121.1, 118.6, 116.2, 115.0, 111.3, 109.7 (d, *J* = 6.8 Hz), 52.3; HRMS (ESI-TOF) calcd. for C_21_H_16_FN_3_O_2_: [M + H]^+^: 362.1305, found 362.1310.

**2-(2-aminophenyl)-*N*-(3-(methylsulfonyl)phenyl)-1*H*-benzimidazol-5(6)-amine (15):** In a round-bottom flask, *N*-(3-(methylsulfonyl)phenyl)-2-(2-nitrophenyl)-1*H*-benzimidazol-5(6)-amine (40 mg, 0.098 mmol), hydrazine monohydrate (50 μL, 1.5 mmol) and Pd/C 5% (8 mg) were mixed in methanol (2.0 mL). The solution was stirred for 30 min at reflux temperature (70 °C). The resulting solution was filtrated and the solvent evaporated. A white solid was obtained in 80% yield (30 mg). ^1^H NMR (400 MHz, CD_3_OD) ppm: 7.68 (dd, *J* = 7.9, 1.5 Hz, 1H_Ar_), 7.54 (m, 2H_Ar_), 7.39 (m, 2H_Ar_), 7.27 (m, 2H_Ar_), 7.18 (m, 1H_Ar_), 7.07 (dd, *J* = 8.6, 2.1 Hz, 1H_Ar_), 6.87 (dd, *J* = 8.2, 1.2 Hz, 1H_Ar_), 6.74 (td, *J* = 7.6, 1.2 Hz, 1H_Ar_), 3.08 (s, 3H_Me_); ^13^C NMR (101 MHz, CD_3_OD) ppm: 154.4, 148.9, 148.5, 142.8, 131.6, 131.3, 128.5, 120.7, 117.9, 117.8, 117.4, 113.5, 113.3, 44.4; HRMS (ESI-TOF) calcd. for C_20_H_19_N_4_O_2_S: [M + H]^+^: 379.1229, found 379.1228.

## 4. Conclusions

The development of a pharmacophore model of *E. coli* DNA Gyrase B inhibitors, followed by docking studies using the crystallographic structure of this target, proved to be a relevant tool for the design and synthesis of the new chemical entities, based on 2-(aminophen-2-yl)-5(6)-substituted-1*H*-benzimidazoles.

The optimization of the condensation/oxidation reaction of 4-bromo-1,2-diaminobenzene with 2-nitrobenzaldehyde, yielding 5(6)-bromo-2-(nitrophen-2-yl)-1*H*-benzimidazole **1**, led us to find a sustainable synthetic approach, using ethanol as solvent and Montmorillonite K10 as a reusable catalyst, which allowed a significant improvement of product yield and isolation process. Additionally, the *N*-boc revealed to be the ideal benzimidazole protecting group since its deprotection significantly does not lead to the formation of the side products obtained when using benzyl protecting group.

These studies pave the way for efficient functionalization of the more synthetically challenging 5(6) position, via cross-coupling Suzuki–Miyaura and Buchwald–Hartwig reactions, using multifunctionalized phenylboronic acids (products **8a** and **9a** were isolated with 72% and 66%, respectively) and amines (3 or 4-(methylsulfonyl)aniline), catalyzed by Pd/XPhos (79–81% isolated yields of **6** and **7**). Finally, the use of NH_2_NH_2_.H_2_O and Pd/C, under air atmosphere, revealed to be a clean strategy to promote the reduction of the nitro group to the corresponding amine, giving **13**, **14**, and **15** with 91%, 56%, and 80% yields respectively.

In summary, we have developed efficient synthetic methods to prepare two different families of 2-(2-aminophenyl)-5(6)-substituted-1*H*-benzimidazoles, which encompass in their structures hydrogen bond donor groups at 2-position, and hydrogen bond acceptor groups at 5(6)- position. According to the predicted docking pose, these groups will favor interactions with Asp73 and Arg136, respectively, and therefore will potentially increase their inhibition potential for *E. coli*’s DNA gyrase B. These new classes of molecules may open new perspectives regarding a broad-range of medicinal applications, particularly for the development of alternative antimicrobial chemical entities effective against *E. coli*. Biological studies are currently underway.

## Figures and Tables

**Table 1:** Selected 2-(2-aminophenyl)-5(6)-substituted-benzimidazole derivatives with corresponding ChemScore and predicted log IC_50_ activity.

#	Structure	ChemScore	Predicted log IC_50_ (nM)
1	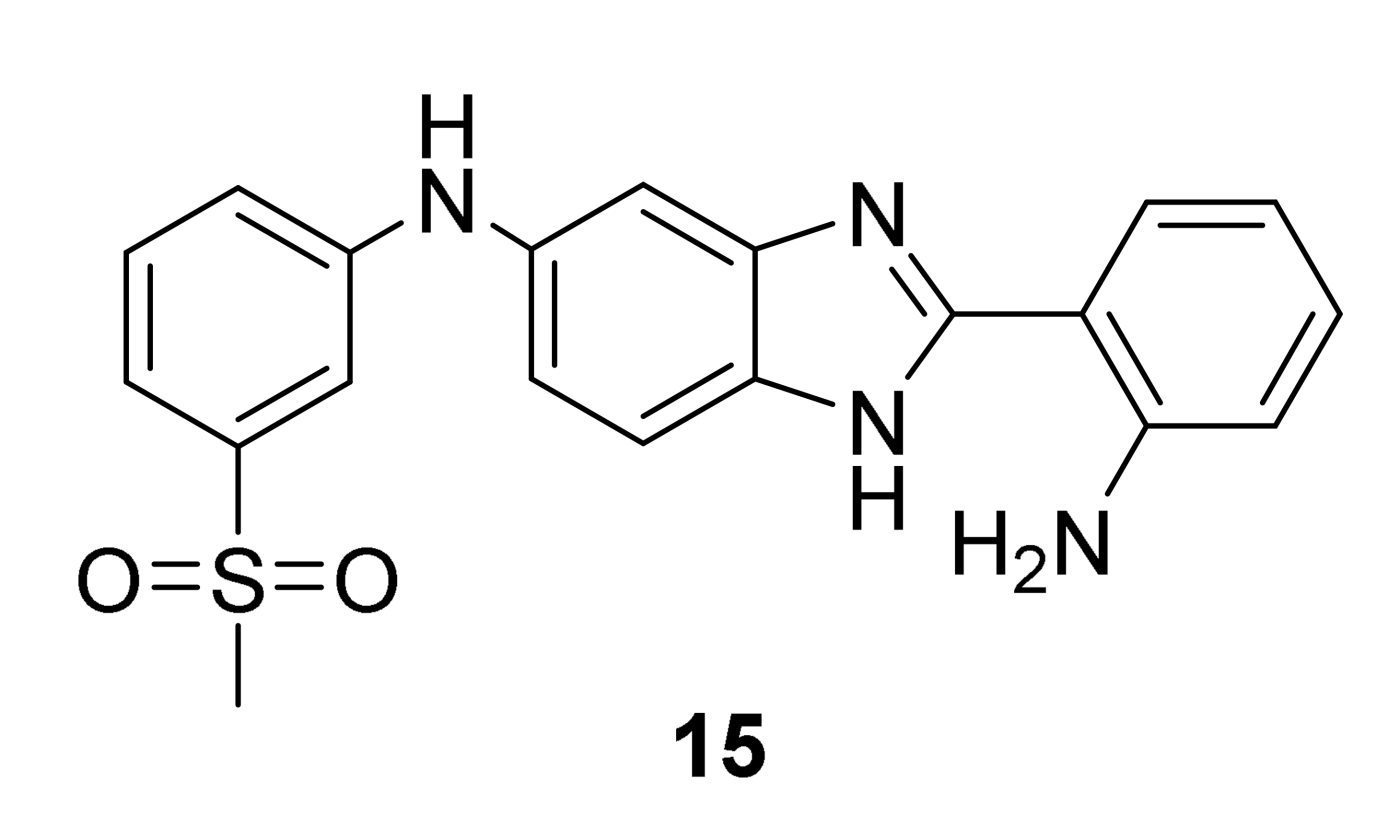	33.7	−0.025 ± 0.56
2	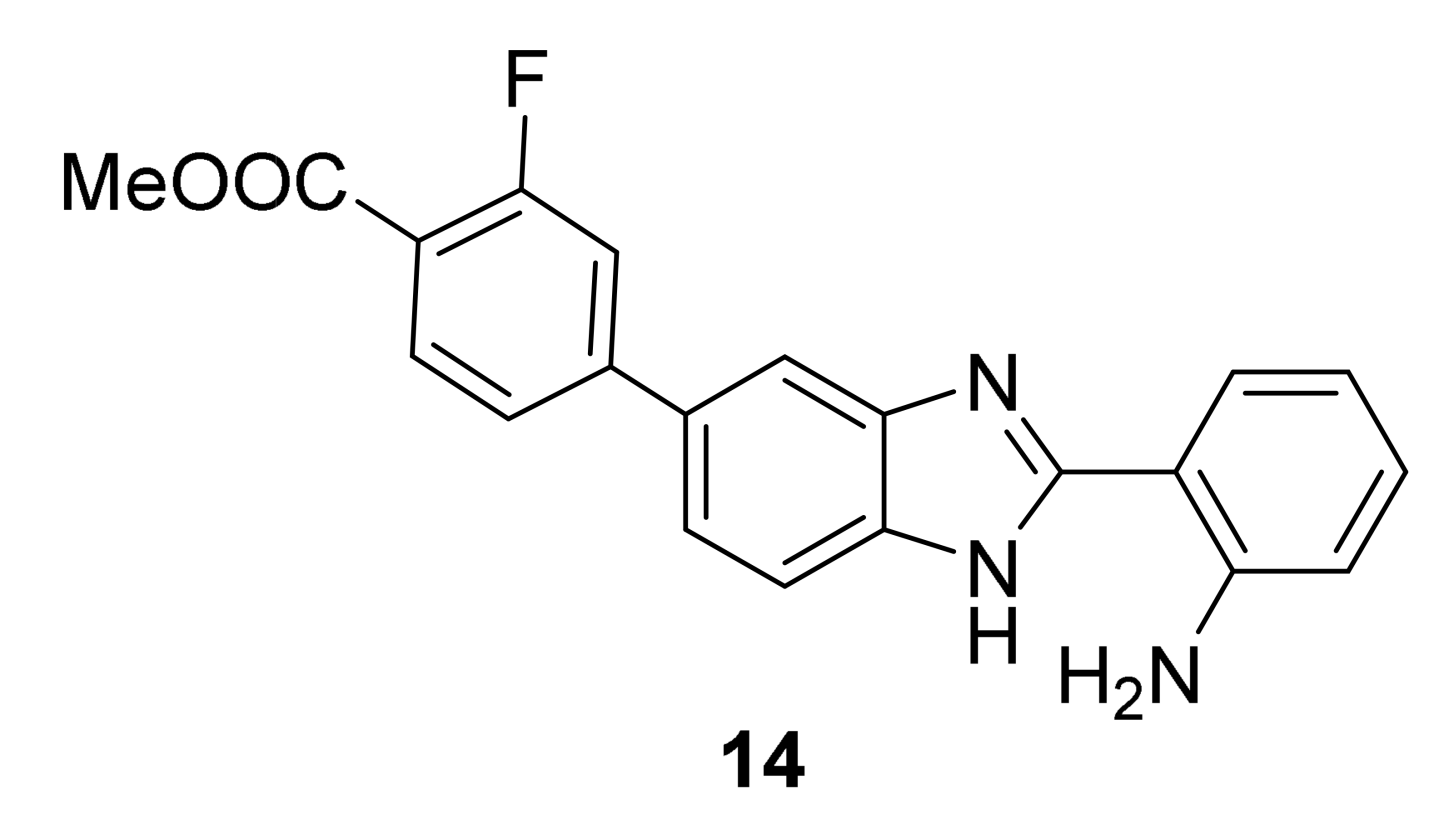	28.7	2.7 ± 1.0
3	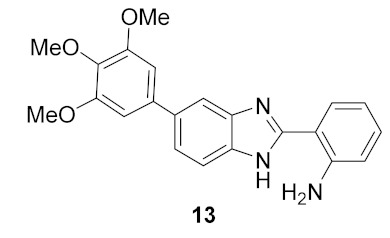	27.5	3.4 ± 1.1

**Table 2:** Optimization of the reaction conditions for the condensation reaction between 4-bromo-1,2-diaminobenzene and 2-nitrobenzaldehyde ^(a)^.

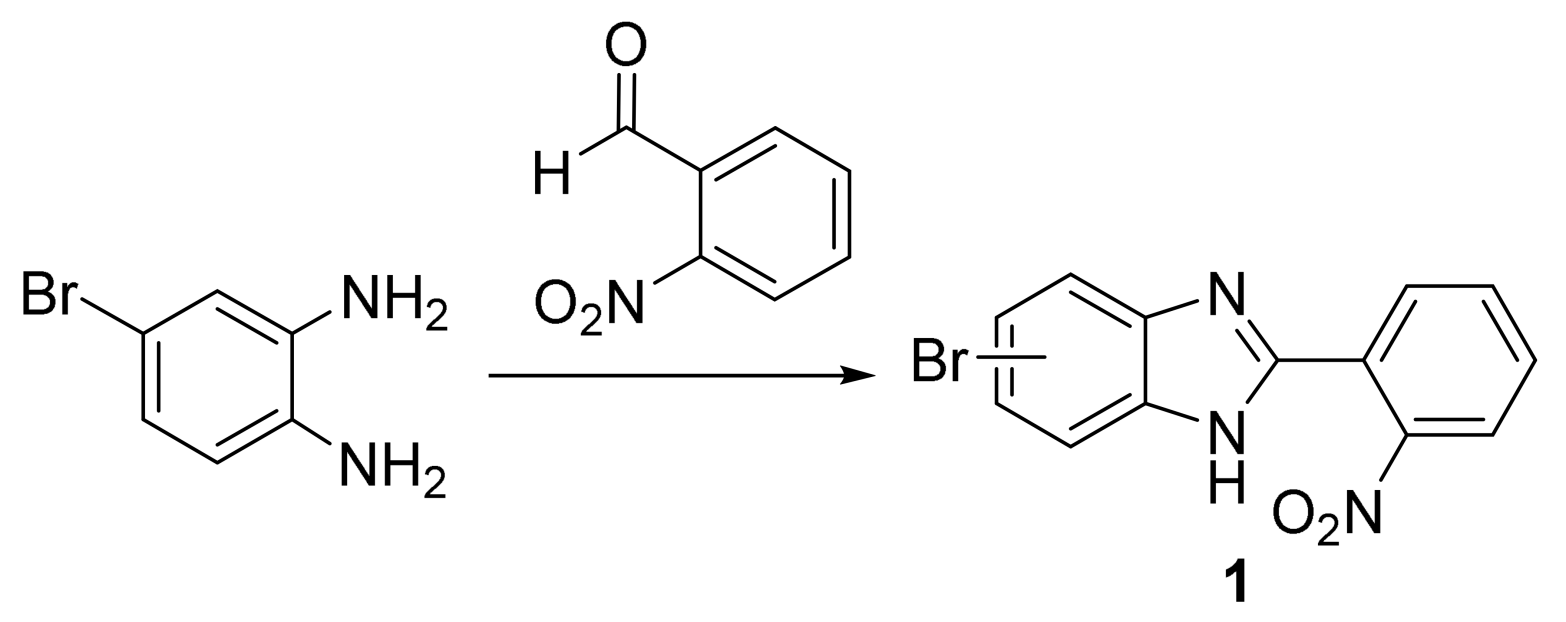					
#	T (°C)	Time (h)	Solvent	Catalyst	Isolated Yield–1 (%)
1	180	8	Nitrobenzene	-	48
2	80	3	Ethanol	-	47
3	25	6	Ethanol	-	50
4	25	4	Ethanol	Montmorillonite K10	62
5	80	2	Ethanol	Montmorillonite K10	54

^(a)^ Typical reaction conditions: 4-bromo-1,2-diaminobenzene (1.1 mmol), 2-nitrobenzaldehyde (1.2 mmol) and the catalyst (20 mg) were mixed in the solvent (3 mL) and heated to the designated temperature, over a variable time period.

**Table 3:** Optimization of the reaction conditions for the Buchwald-Hartwig coupling of benzyl protected benzimidazole **2** with 4-(methylsulfonyl)aniline ^(a)^.

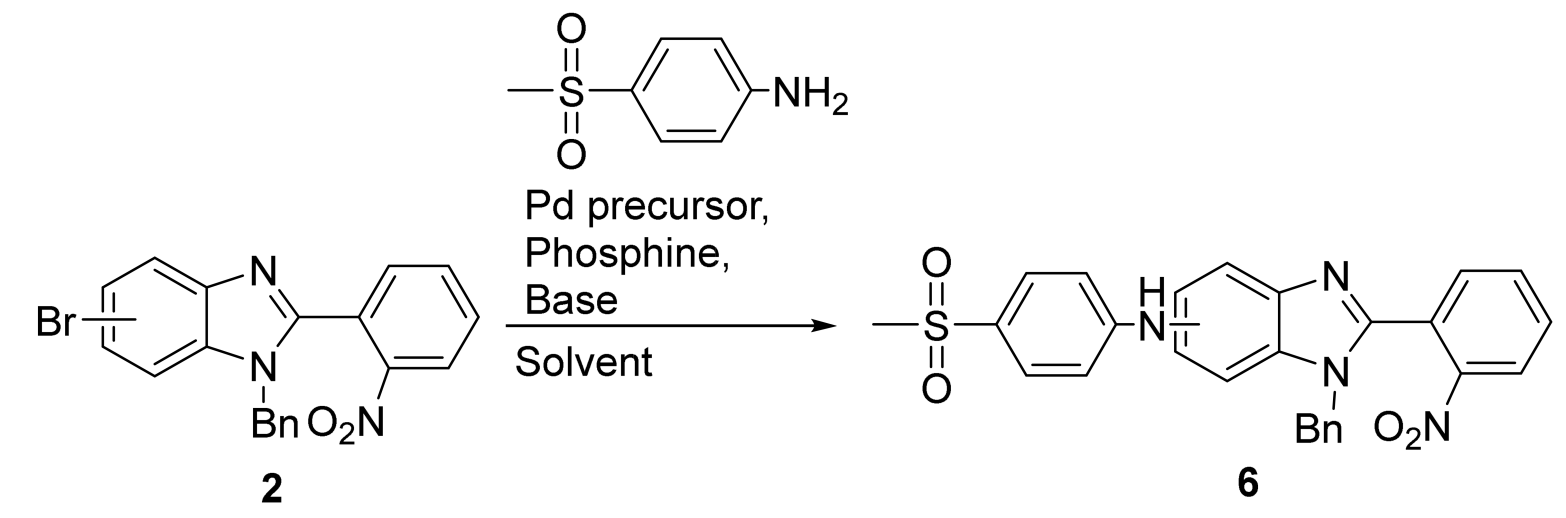				
#	Phosphine	Solvent	Sub/Cat	Conv (%)
1	15 mol% BINAP	Toluene	10	trace
2	15 mol% DPEphos	Toluene	10	trace
3	15 mol% XPhos	Toluene	10	91
4	15 mol% BINAP	Dioxane	10	trace
5	15 mol% DPEphos	Dioxane	10	trace
6	15 mol% XPhos	Dioxane	10	100 (78)^b)^
7^c)^	15 mol% XPhos	Dioxane	10	79
8	7.5 mol% XPhos	Dioxane	20	57

BINAP:(2,2′-bis(diphenylphosphino)-1,1′-binaphthyl); DPEphos: bis[(2-diphenylphosphino)phenyl] ether; Xphos: [2-Dicyclohexylphosphino-2′,4′,6′-triisopropylbiphenyl]. ^(a)^ Typical reaction conditions: **2** (0.74 mmol); 4-(methylsulfonyl)aniline (0.88 mmol); Cs_2_CO_3_ (1.47 mmol); Pd(OAc)_2_ (0.074 mmol); phosphine (0.11 mmol); solvent (5 mL); Temperature = 100 °C; Time = 16 h; (**b**) Isolated yield; (**c**) Time = 8 h.

## Data Availability

The data presented in this paper are available in Supplementary Materials.

## Supplementary Materials

The following are available online. Figure S2: Training set for the development of *Escherichia coli*’s GyrB pharmacophore model; Figure S3: Computational pharmacophore model and docking test/correlation data; Figure S4: Structure of phosphines used; Figures S5–S60: ^1^H and ^13^C NMR and HRMS (ESI-TOF) spectra for all synthesized compounds.
