# Supplementary material for: Synthesis of Computationally Designed 2,5(6)-Benzimidazole Derivatives via Pd-Catalyzed Reactions for Potential E. coli DNA Gyrase B Inhibition
Source: Molecules. 2021 Mar 2;26(5):1326. doi: 10.3390/molecules26051326 (PMC7958342; doi:10.3390/molecules26051326)
Supplement: Supplementary file 1 [file molecules-26-01326-s001.pdf]

Supporting Information (SI)

Table of contents:

Computational chemistry data **S2**

Structures of phosphines used **S4**

Structural characterization data **S5**

5(6)-bromo-2-(nitrophen-2-yl)-1*H*-benzimidazole **(1)** S5

1-benzyl-5(6)-bromo-2-(nitrophen-2-yl)-1H-benzimidazole **(2)** S11

1-boc-5-bromo-2-(nitrophen-2-yl)-1*H*-benzimidazole **(3a)** S14

1-boc-6-bromo-2-(nitrophen-2-yl)-1*H*-benzimidazole **(3b)** S21

1-benzyl-5(6)-(3,4,5-trimetoxiphen-1-yl)-2-(nitrophen-2-yl)-1*H*-benzimidazole **(4)** S25

1-benzyl-5(6)-(3-fluoro-4-(methoxycarbonyl)phen-1-yl)-2-(nitrophen-2-yl)-1*H*-benzimidazole **(5)** ... S28

1-benzyl-*N*-(4-(methylsulfonyl)phenyl)-2-(nitrophen-2-yl)-1-benzimidazol-5(6)-amine **(6)** S31

1-benzyl-*N*-(3-(methylsulfonyl)phenyl)-2-(nitrophen-2-yl)-1*H*-benzimidazol-5(6)-amine **(7)** S34

1-boc-5-(3,4,5-trimetoxiphen-1-yl)-2-(nitrophen-2-yl)-1*H*-benzimidazole **(8a)** S37

1-boc-5-(3-fluoro-4-(methoxycarbonyl)phen-1-yl)-2-(nitrophen-2-yl)-benzimidazole **(9a)** S40

N-(3-(methylsulfonyl)phenyl)-2-(nitrophen-2-yl)-1*H*-benzimidazol-5(6)-amine **(10)** S43

5(6)-(3,4,5-trimetoxiphen-1-yl)-2-(nitrophen-2-yl)-1*H*-benzimidazole **(11)** S46

5(6)-(3-fluoro-4-(methoxycarbonyl)phen-1-yl)-2-(nitrophen-2-yl)-1*H*-benzimidazole **(12)** S49

2-(aminophen-2-yl)-5(6)-(3,4,5-trimetoxiphen-1-yl)-1*H*-benzimidazole **(13)** S52

2-(aminophen-2-yl)-5(6)-(3-fluoro-4-(methoxycarbonyl)phen-1-yl)-1*H*-benzimidazole **(14)** S55

2-(aminophen-2-yl)-*N*-(3-(methylsulfonyl)phenyl)-1*H*-benzimidazol-5(6)-amine **(15)** S58

Computational chemistry data

Training set for the development of *Escherichia coli*'s GyrB pharmacophore model

Active molecules (IC_{50} ≤ 1.0 μM) that comprise the training set.

**Compound:** 1<br>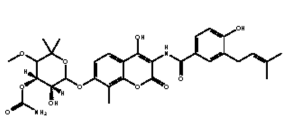<br>**IC50 (μM):** 0.1700	**Compound:** 2<br>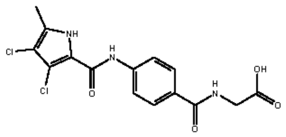<br>**IC50 (μM):** 0.2800	**Compound:** 3<br>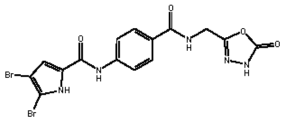<br>**IC50 (μM):** 0.0800	**Compound:** 4<br>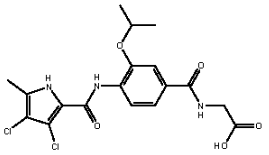<br>**IC50 (μM):** 0.0470
**Compound:** 5<br>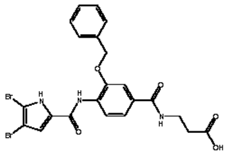<br>**IC50 (μM):** 0.0870	**Compound:** 6<br>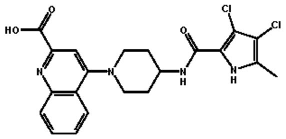<br>**IC50 (μM):** 0.0009	**Compound:** 7<br>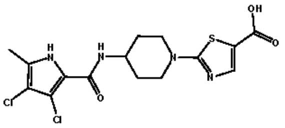<br>**IC50 (μM):** 0.0250	**Compound:** 8<br>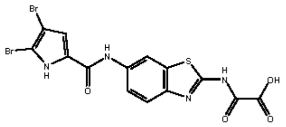<br>**IC50 (μM):** 0.0380
**Compound:** 9<br>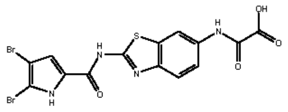<br>**IC50 (μM):** 0.0580	**Compound:** 10<br>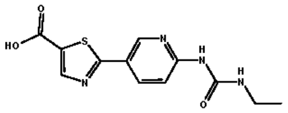<br>**IC50 (μM):** 0.2400	**Compound:** 11<br>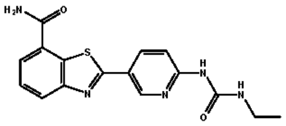<br>**IC50 (μM):** 0.0940	**Compound:** 12<br>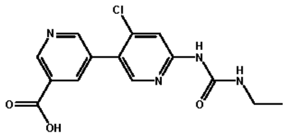<br>**IC50 (μM):** 0.0580
**Compound:** 13<br>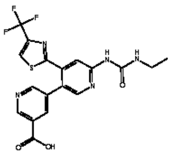<br>**IC50 (μM):** 0.0100	**Compound:** 14<br>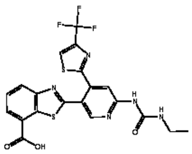<br>**IC50 (μM):** 0.0100	**Compound:** 15<br>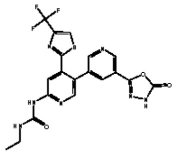<br>**IC50 (μM):** 0.0100	**Compound:** 16<br>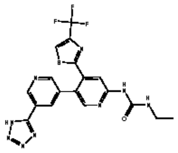<br>**IC50 (μM):** 0.0100
**Compound:** 17<br>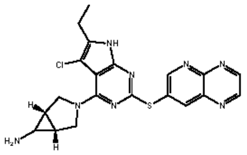<br>**IC50 (μM):** 0.0010	**Compound:** 18<br>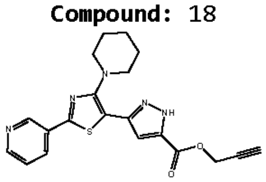<br>**IC50 (μM):** 0.0110		

A: Performance of the selected pharmacophore model against a dataset of know actives, moderately actives and inactives

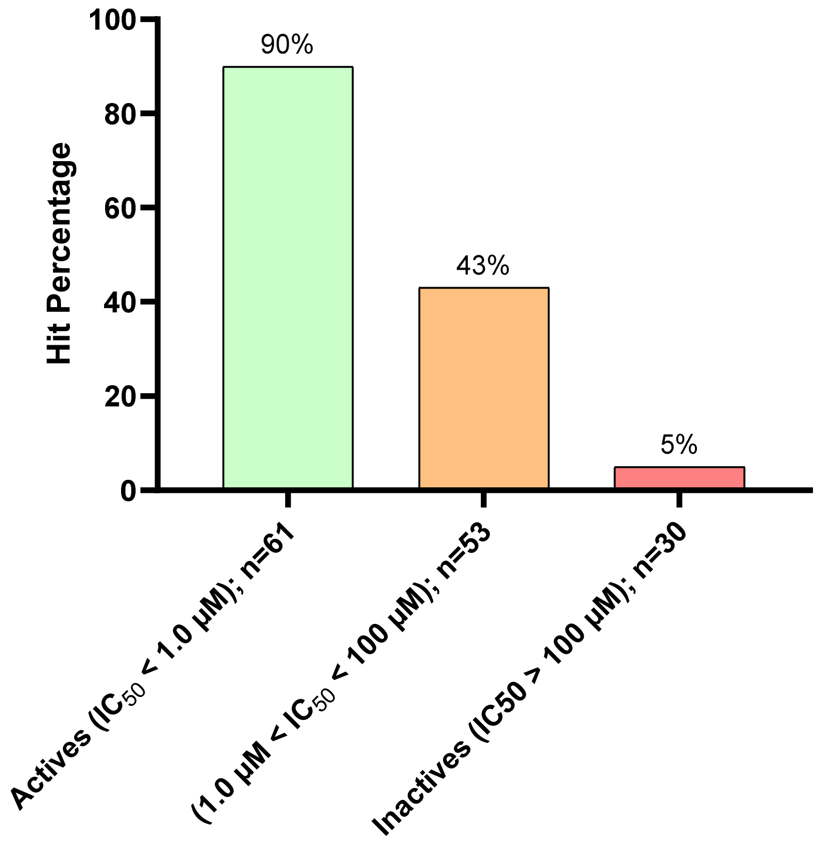

B:Correlation between GOLD ChemScore values and experimental IC_{50} values from molecules retrieved from literature

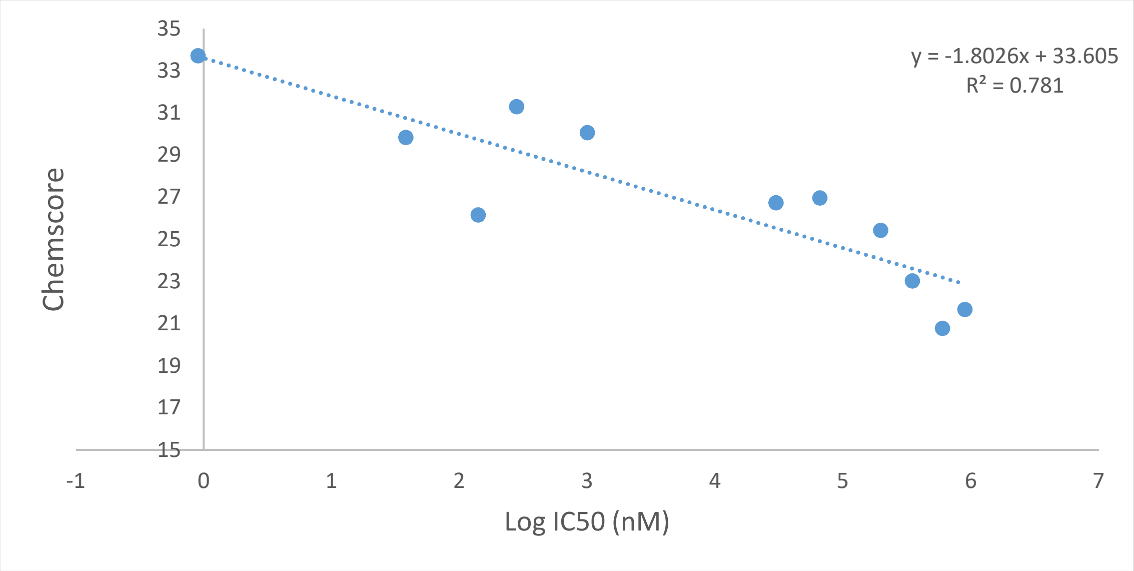

Structures of phosphines used

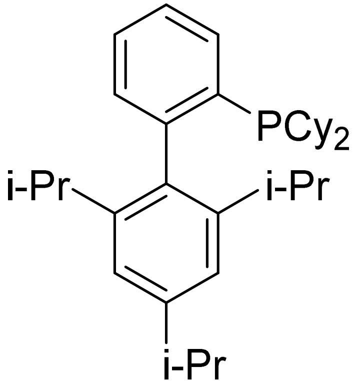

XPhos: [2-Dicyclohexylphosphino-2’,4’,6’-triisopropylbiphenyl]

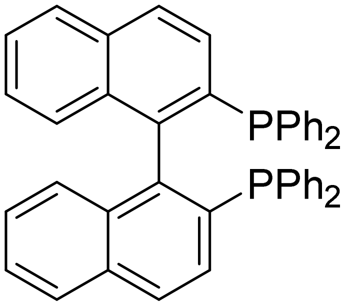

BINAP: (2,2’-bis(diphenylphosphino)-1,1’-binaphthyl)

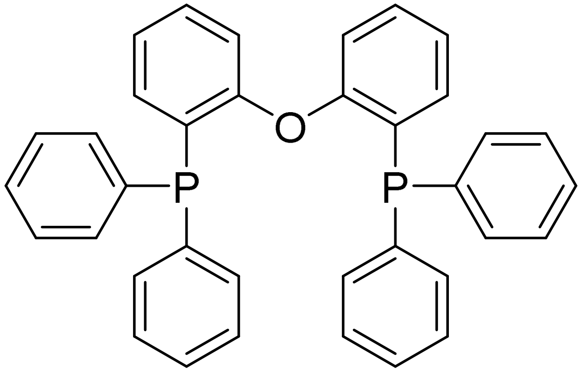

DPEphos: bis[(2-diphenylphosphino)phenyl] ether

5(6)-bromo-2-(nitrophen-2-yl)-1*H*-benzimidazole **(1)**

^{1}H NMR (400 MHz, DMSO-d_6)

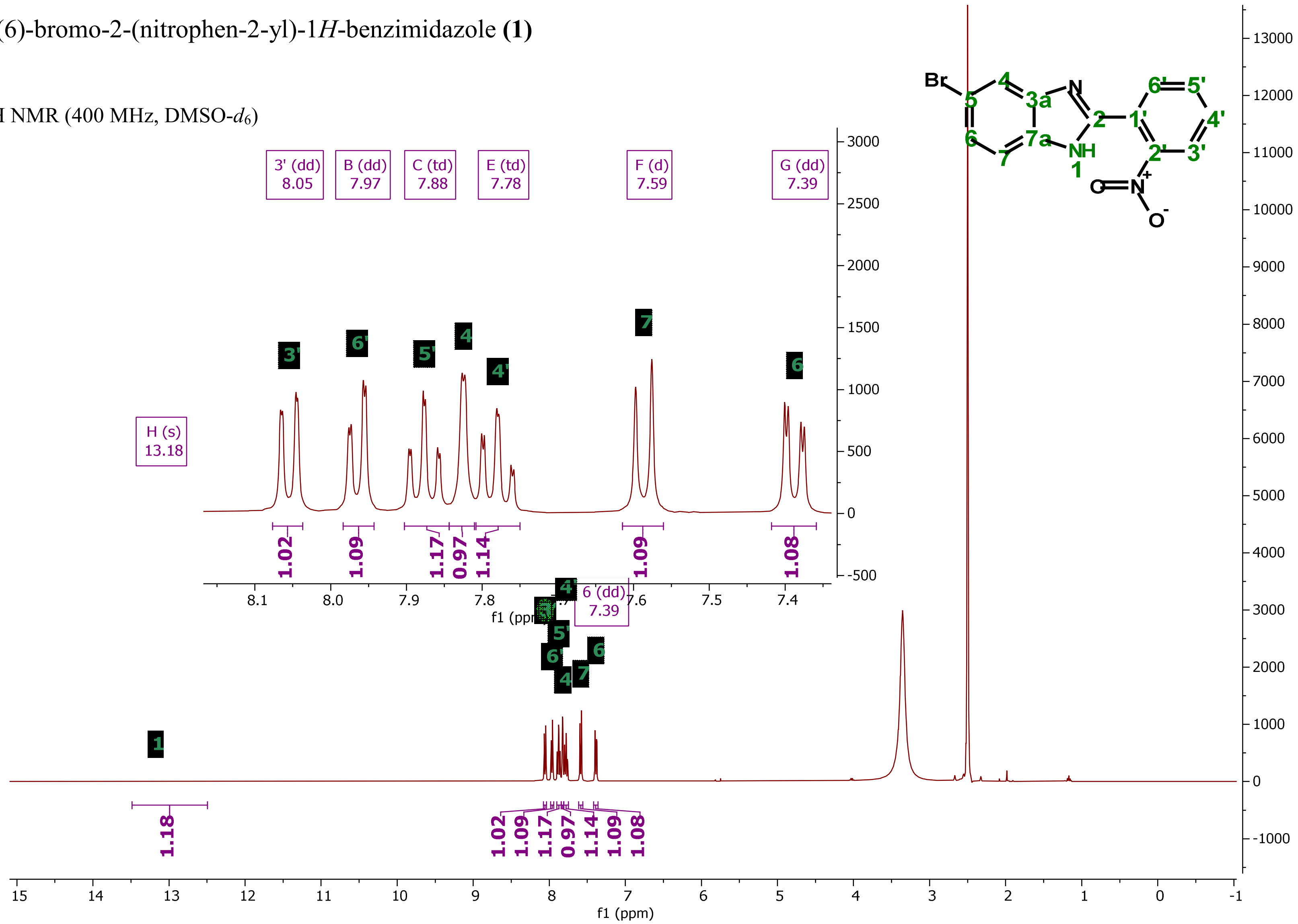

$^{13}C\{^1H\}$ NMR (101 MHz, DMSO-d_6)

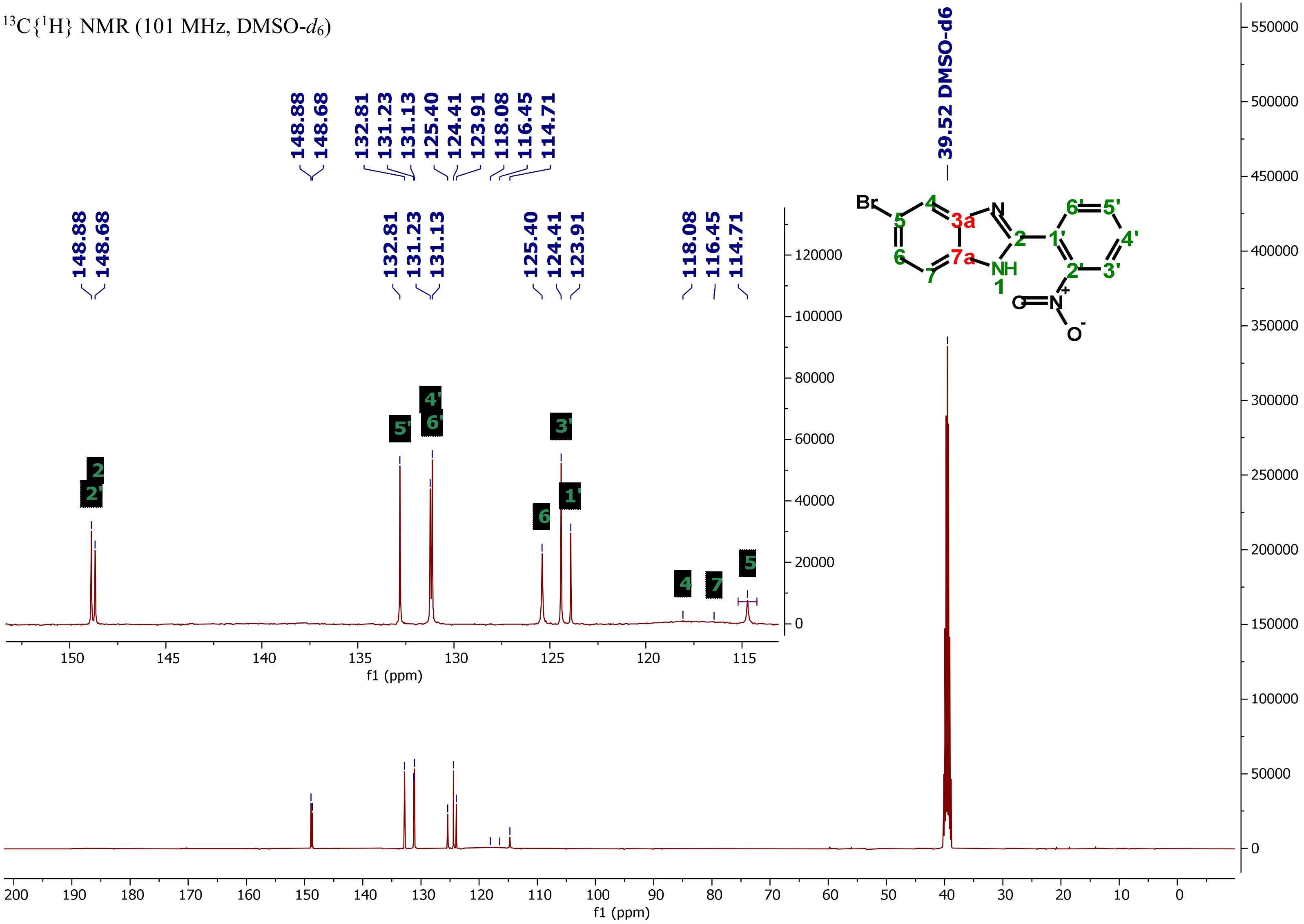

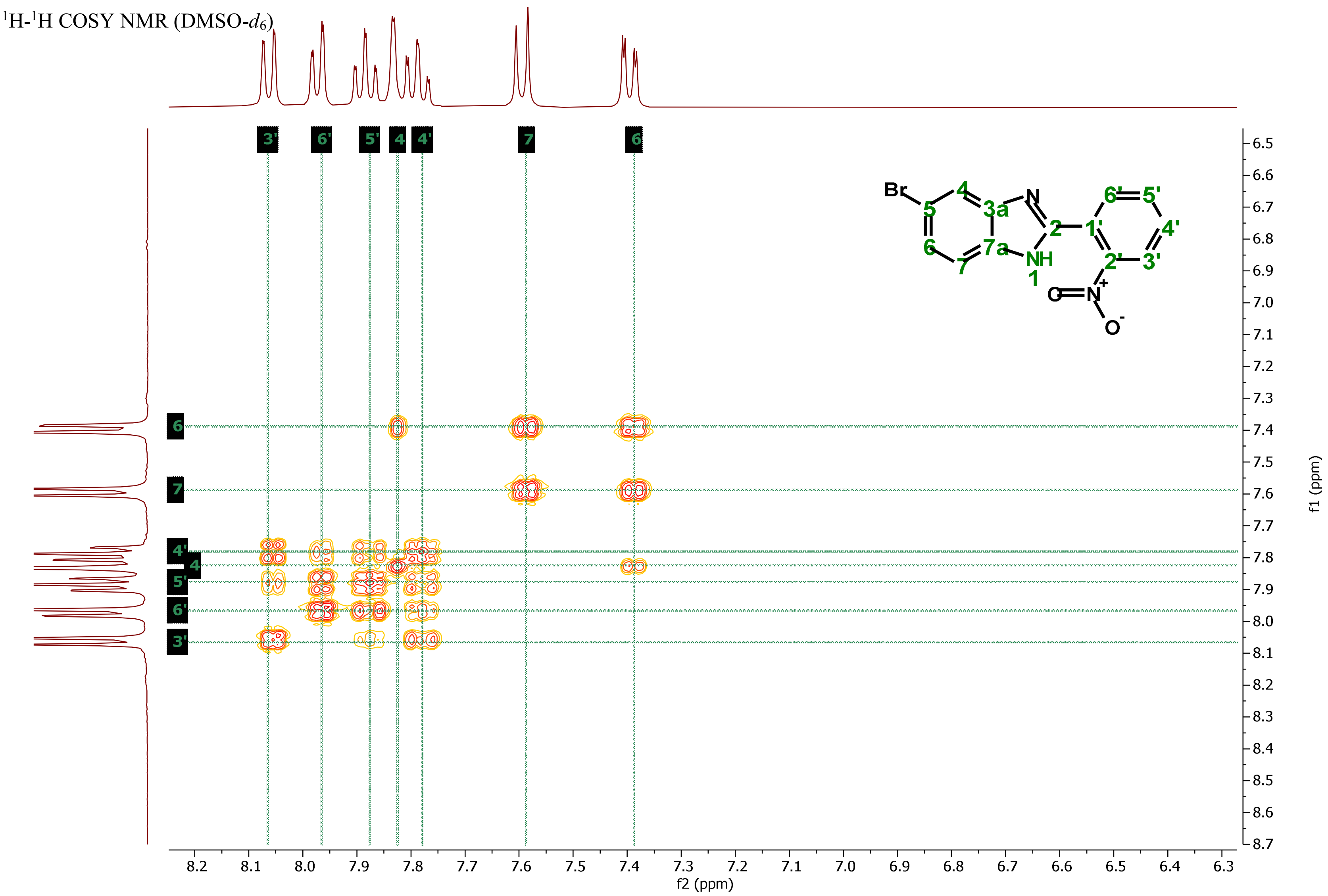
^{1}H-^{1}H COSY NMR (DMSO-d_6)
3'
6'
5'
4
4'
7
6
Br
N
NH
1
O
N
O
f2 (ppm)
f1 (ppm)

^{1}H-^{13}C HSQC NMR (DMSO-d_6)

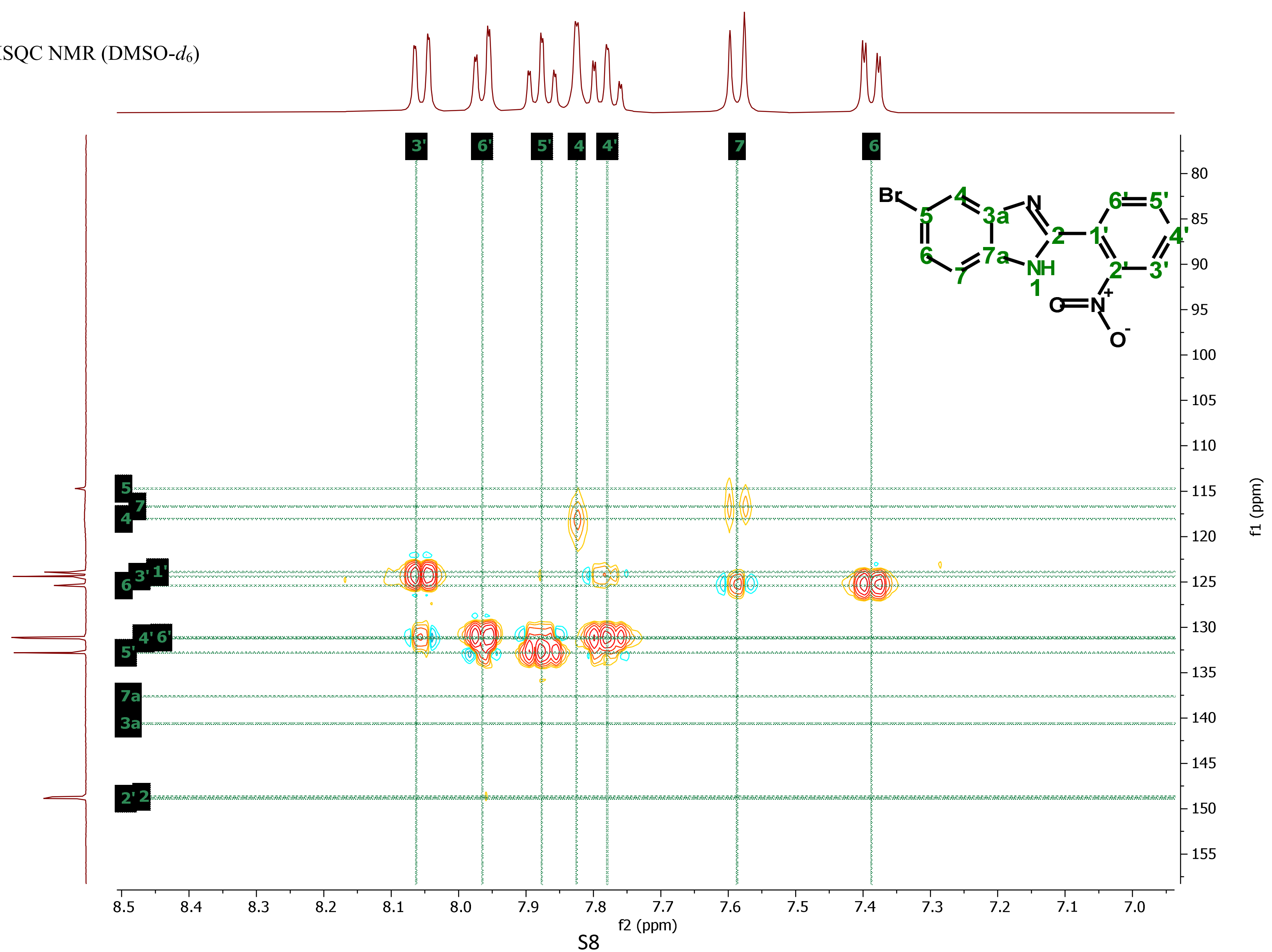

^{1}H-^{13}C HMBC NMR (DMSO-d_6)

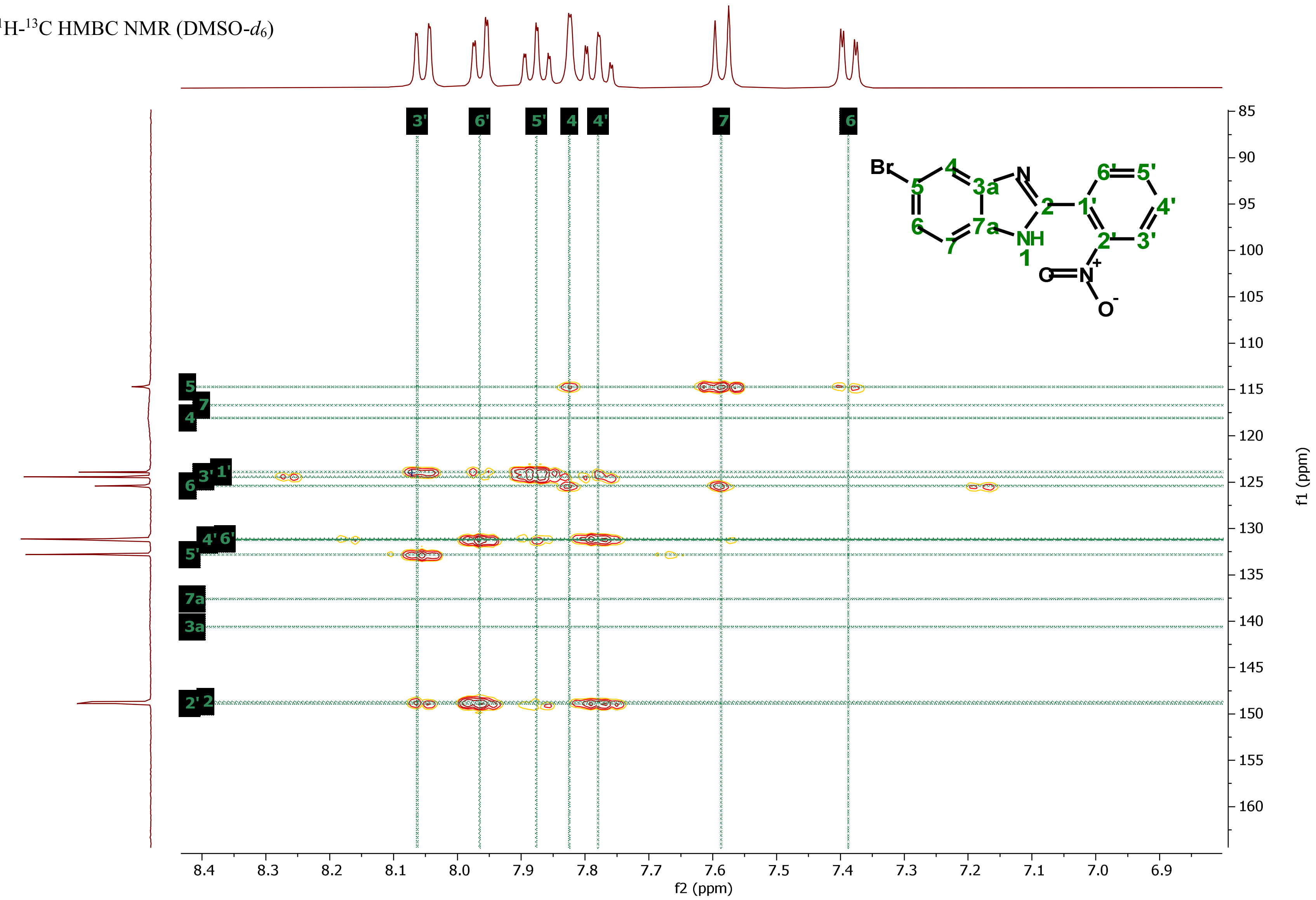

HRMS (ESI-TOF)

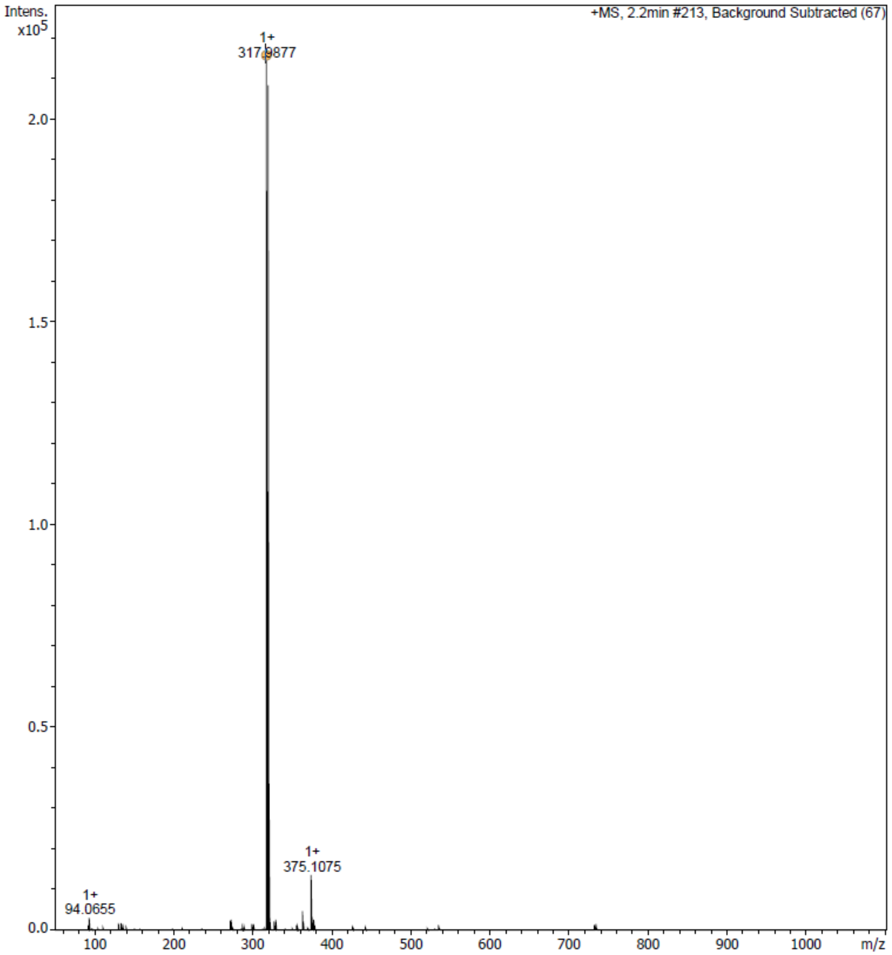

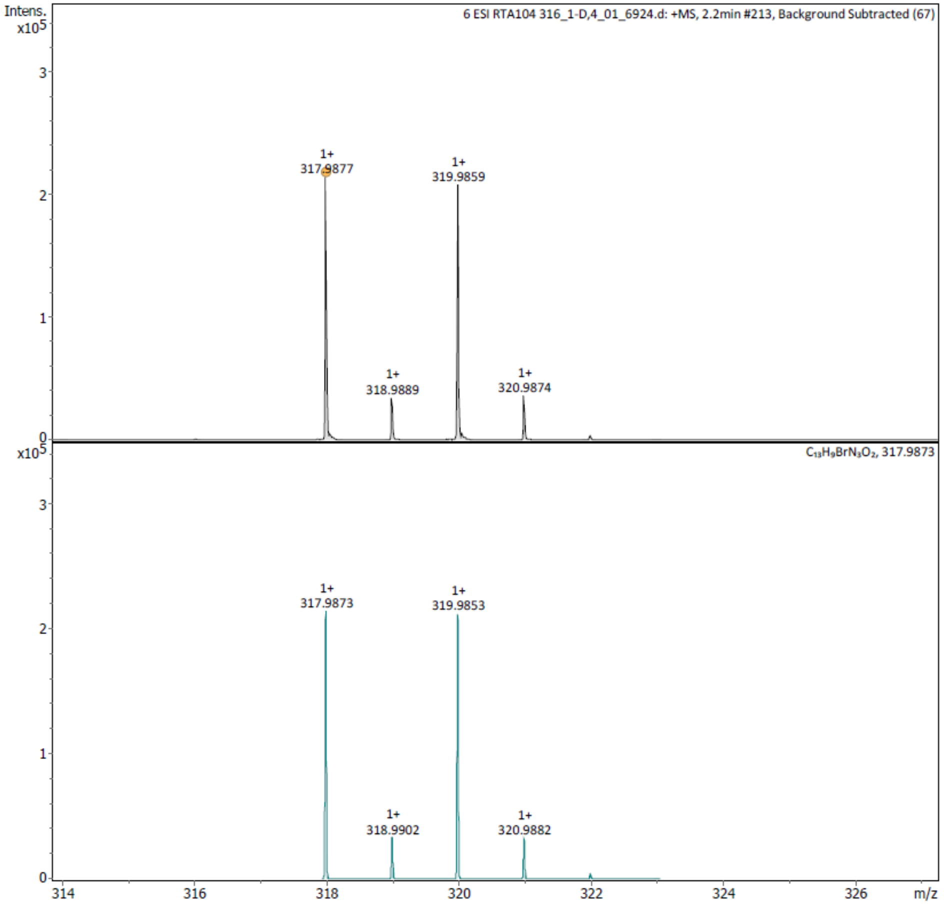

Meas. m/z	#	Ion Formula	Score	m/z	err [mDa]	err [ppm]	mSigma	rdb	e¯ Conf	N-Rule
317.9877	1	C15H11BrO3	84.33	317.9886	0.9	2.7	9.8	12.5	odd	ok
	2	C13H9BrN3O2	100.00	317.9873	-0.5	-1.5	11.6	13.0	even	ok

1-benzyl-5(6)-bromo-2-(nitrophen-2-yl)-1H-benzimidazole **(2)**

^{1}H NMR (400 MHz, $CDCl_3$)

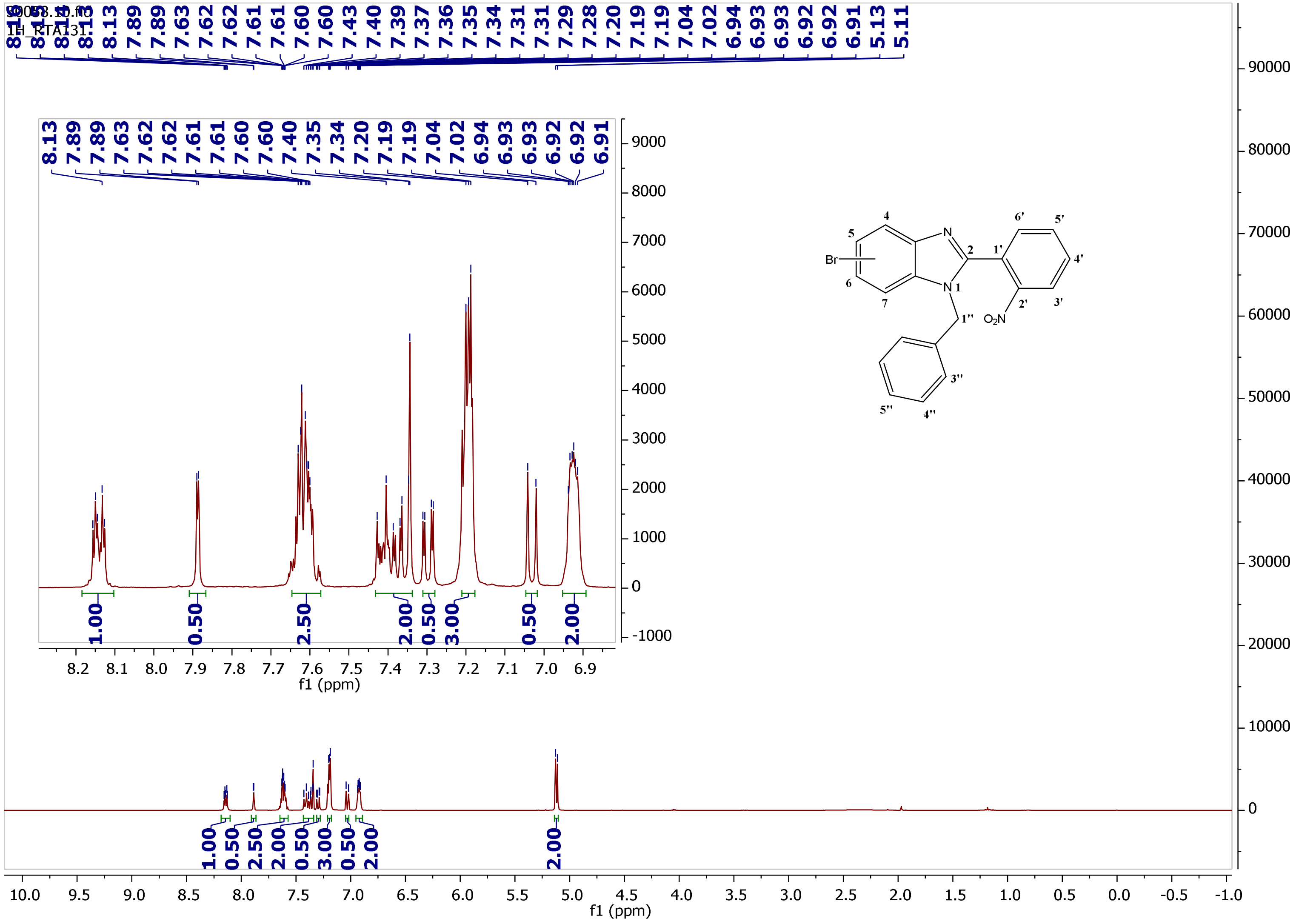

$^{13}C\{^1H\}$ NMR (101 MHz, $CDCl_3$)

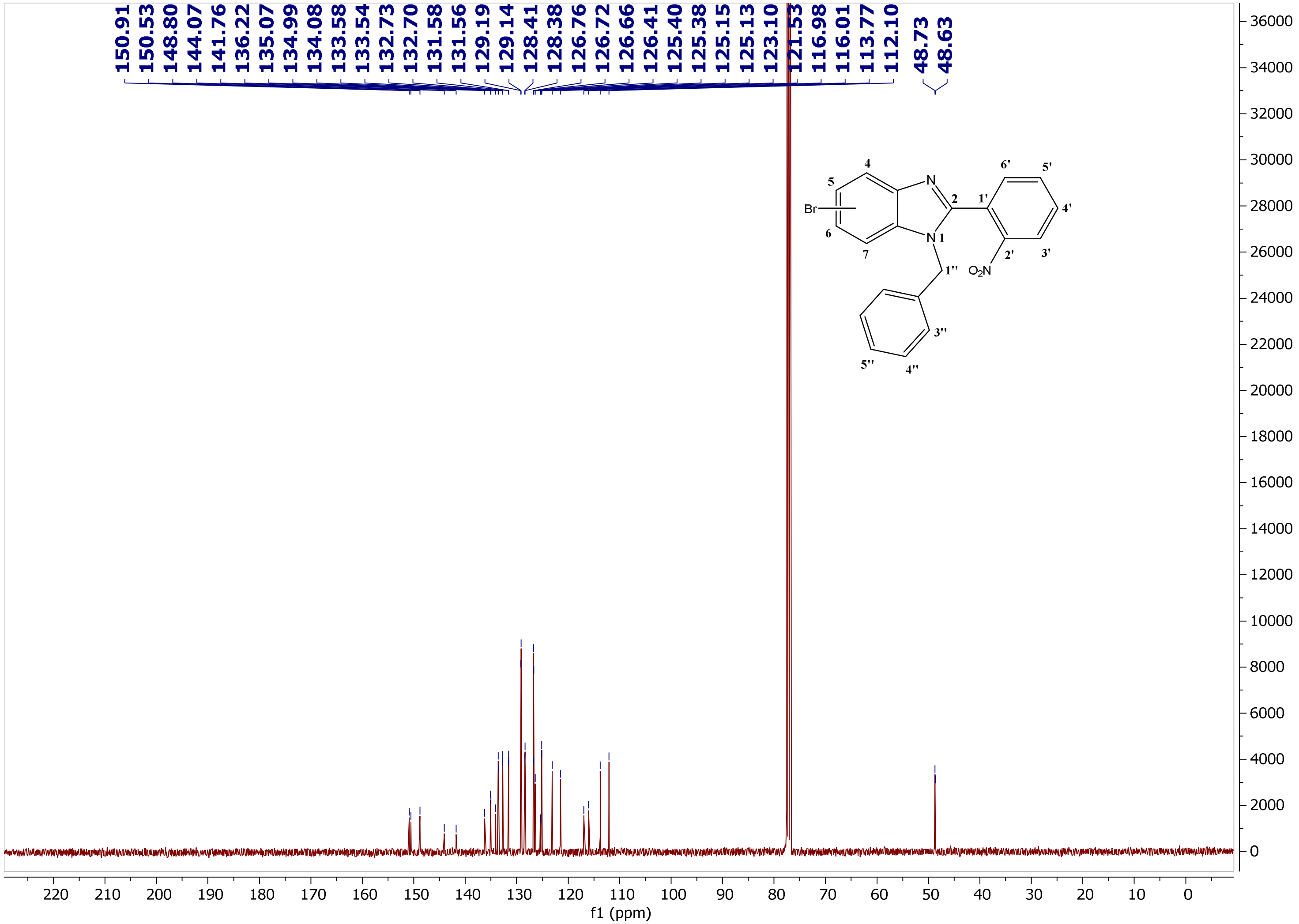

HRMS (ESI-TOF):

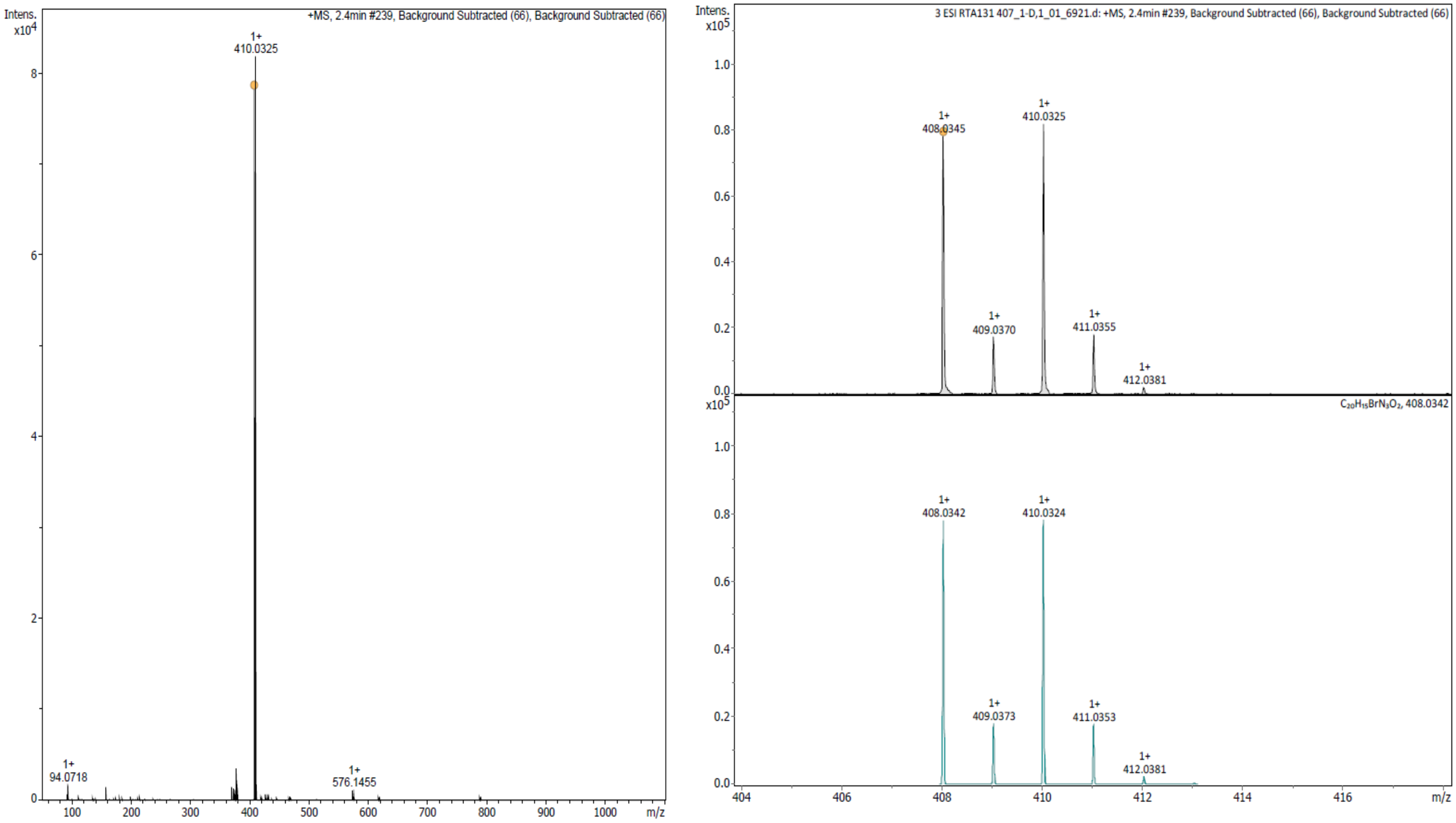
Intens.
x10^4
+MS, 2.4min #239, Background Subtracted (66), Background Subtracted (66)
1+
410.0325
1+
94.0718
1+
576.1455
m/z
Intens.
x10^5
3 ESI RTA131 407_1-D,1_01_6921.d: +MS, 2.4min #239, Background Subtracted (66), Background Subtracted (66)
1+
408.0345
1+
410.0325
1+
409.0370
1+
411.0355
1+
412.0381
x10^5
C20H15BrN3O2, 408.0342
1+
408.0342
1+
410.0324
1+
409.0373
1+
411.0353
1+
412.0381
m/z

1-boc-5-bromo-2-(nitrophen-2-yl)-1H-benzimidazole **(3a)**

^{1}H NMR (400 MHz, $CDCl_3$)

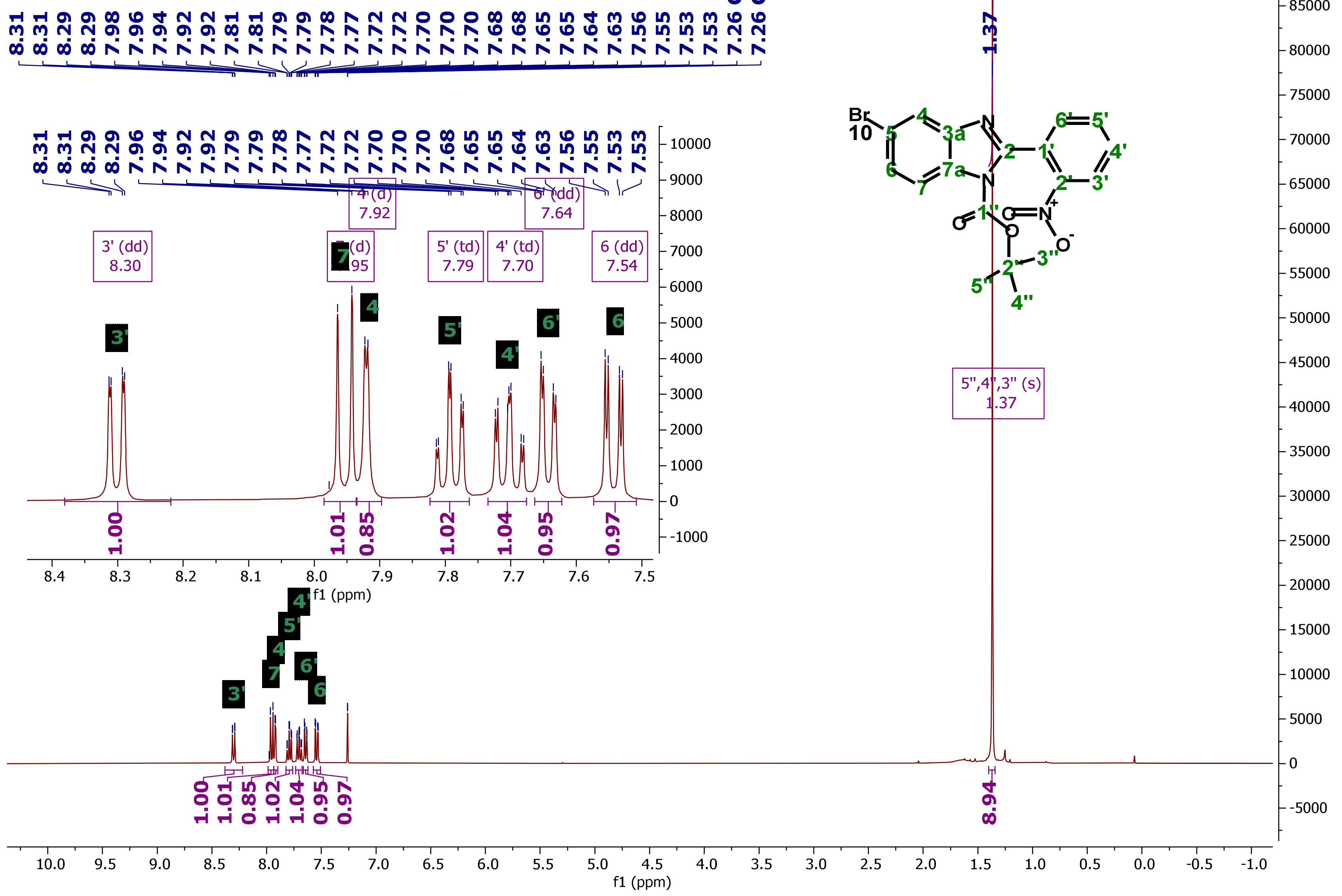

$^{13}C\{^1H\}$ NMR (101 MHz, $CDCl_3$)

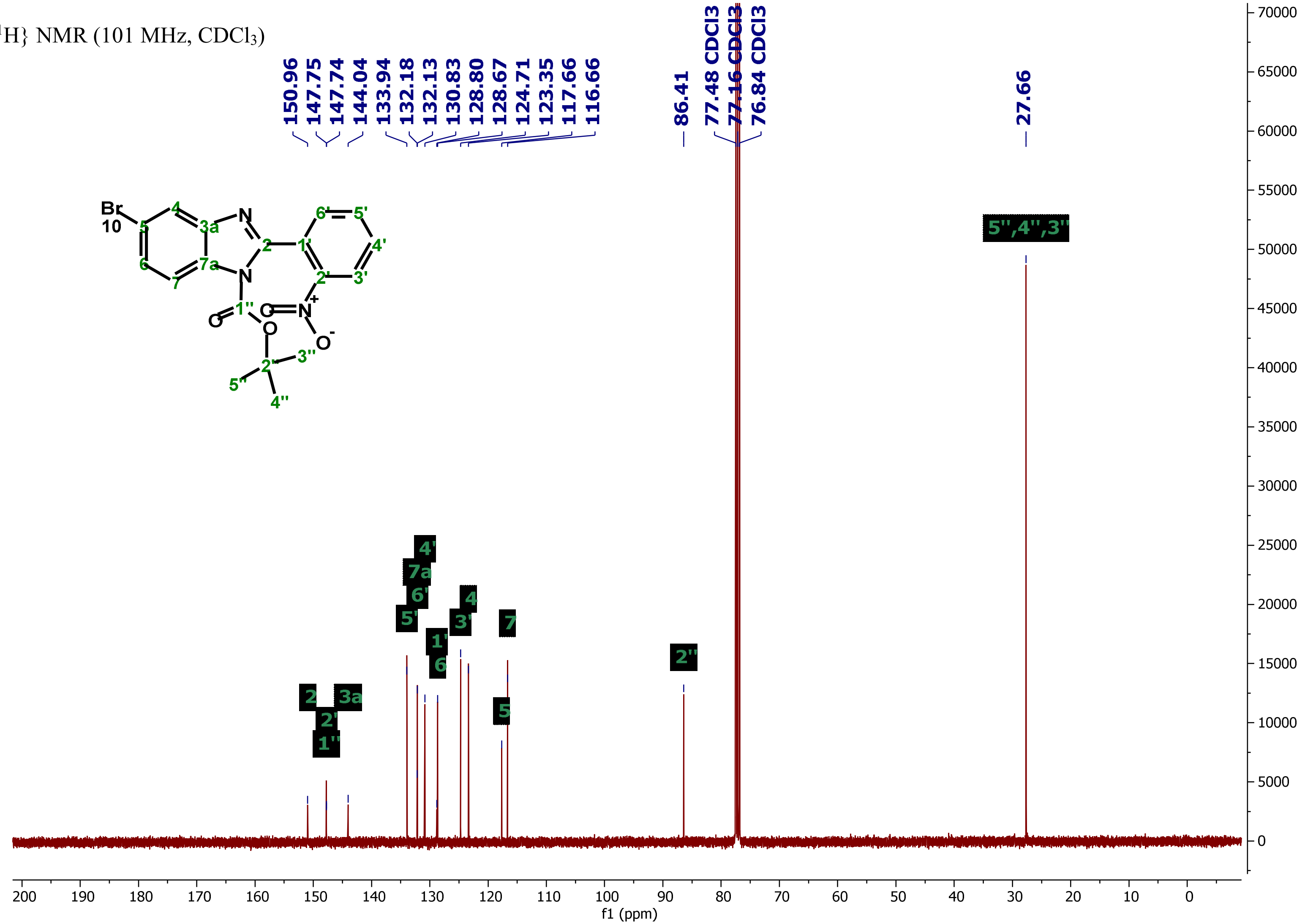

$^{13}C\{^{1}H\}$ DEPT-135 NMR (101 MHz, $CDCl_3$)

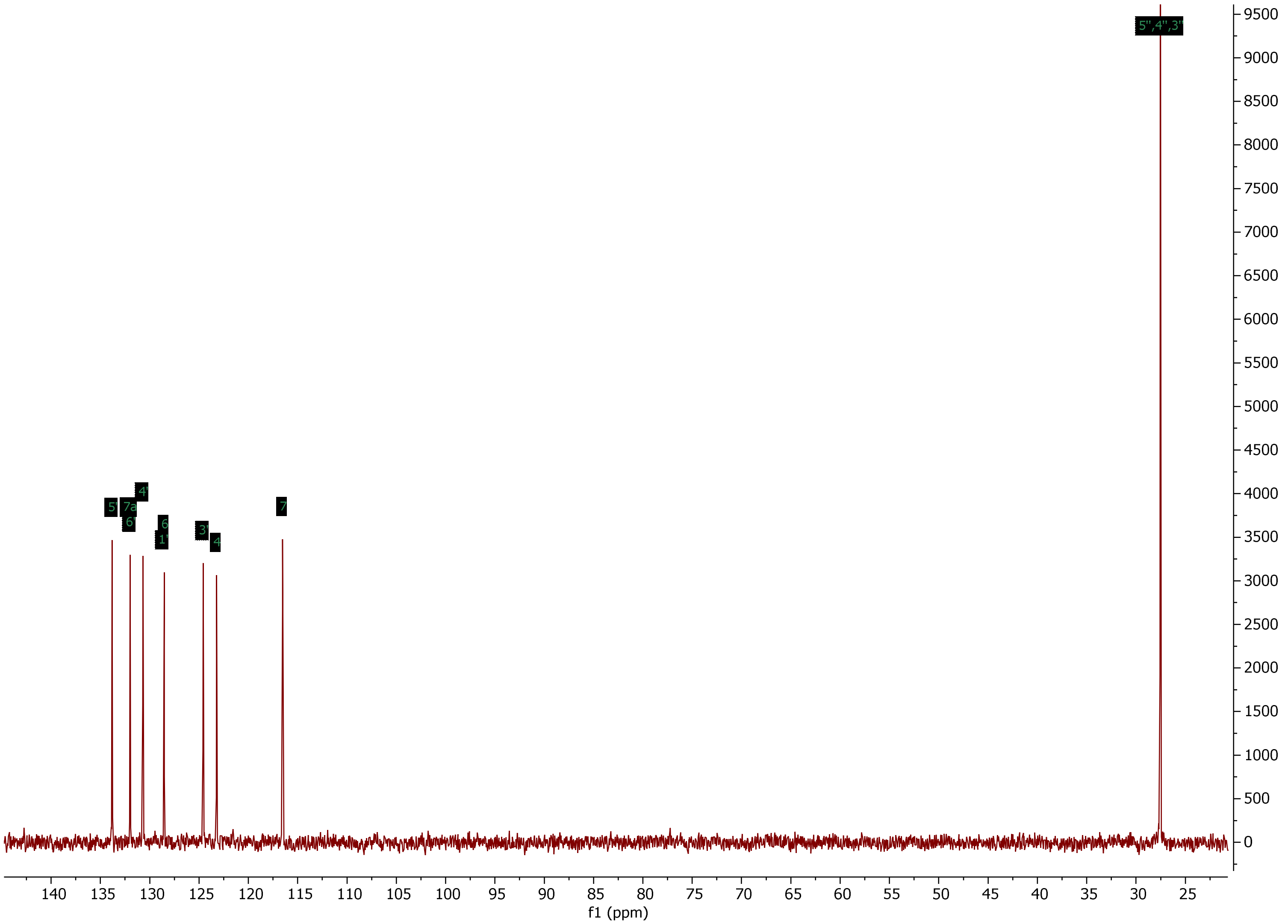

^{1}H-^{1}H COSY NMR ($CDCl_3$)

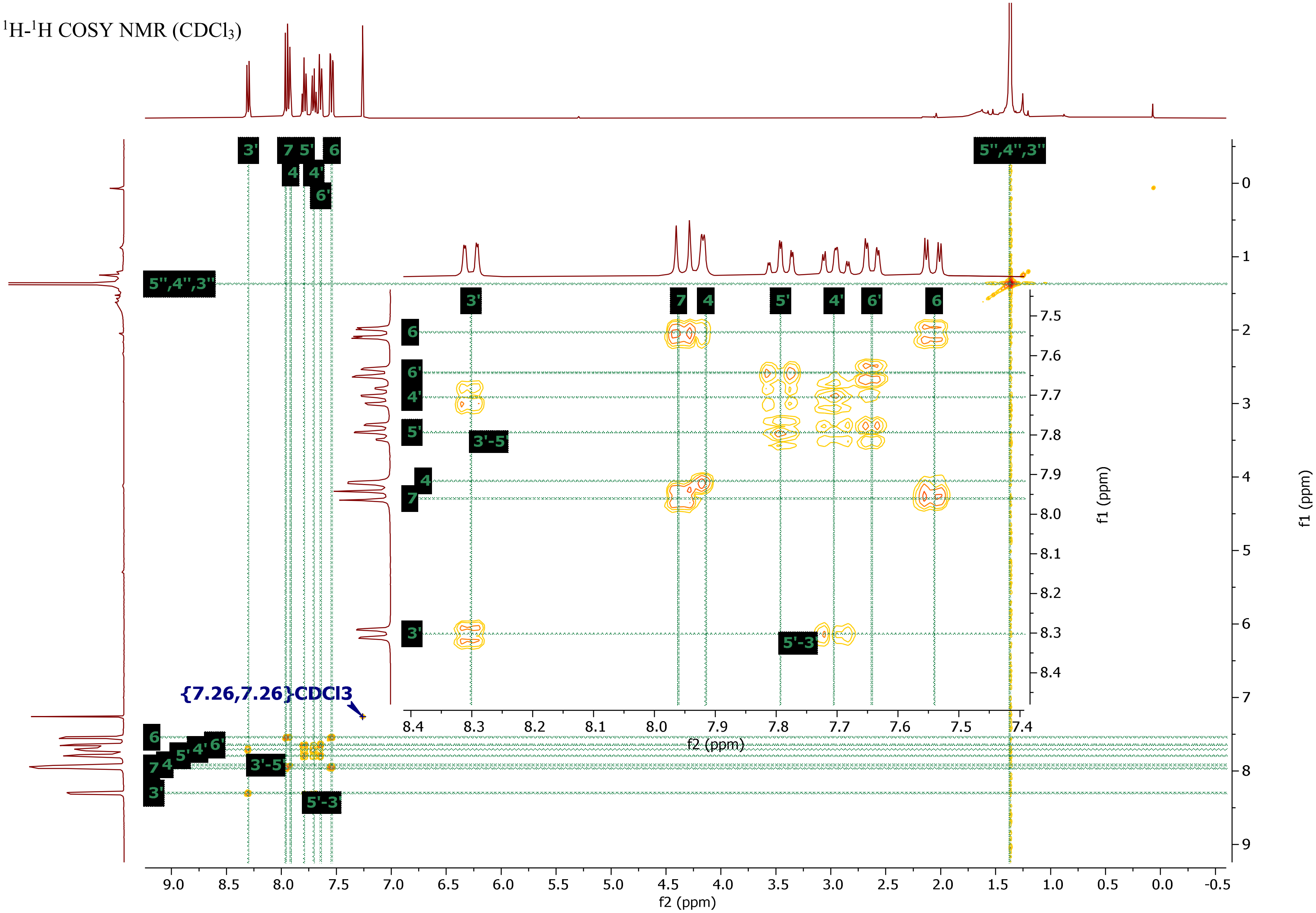

^{1}H-^{1}H NOESY NMR ($CDCl_3$)

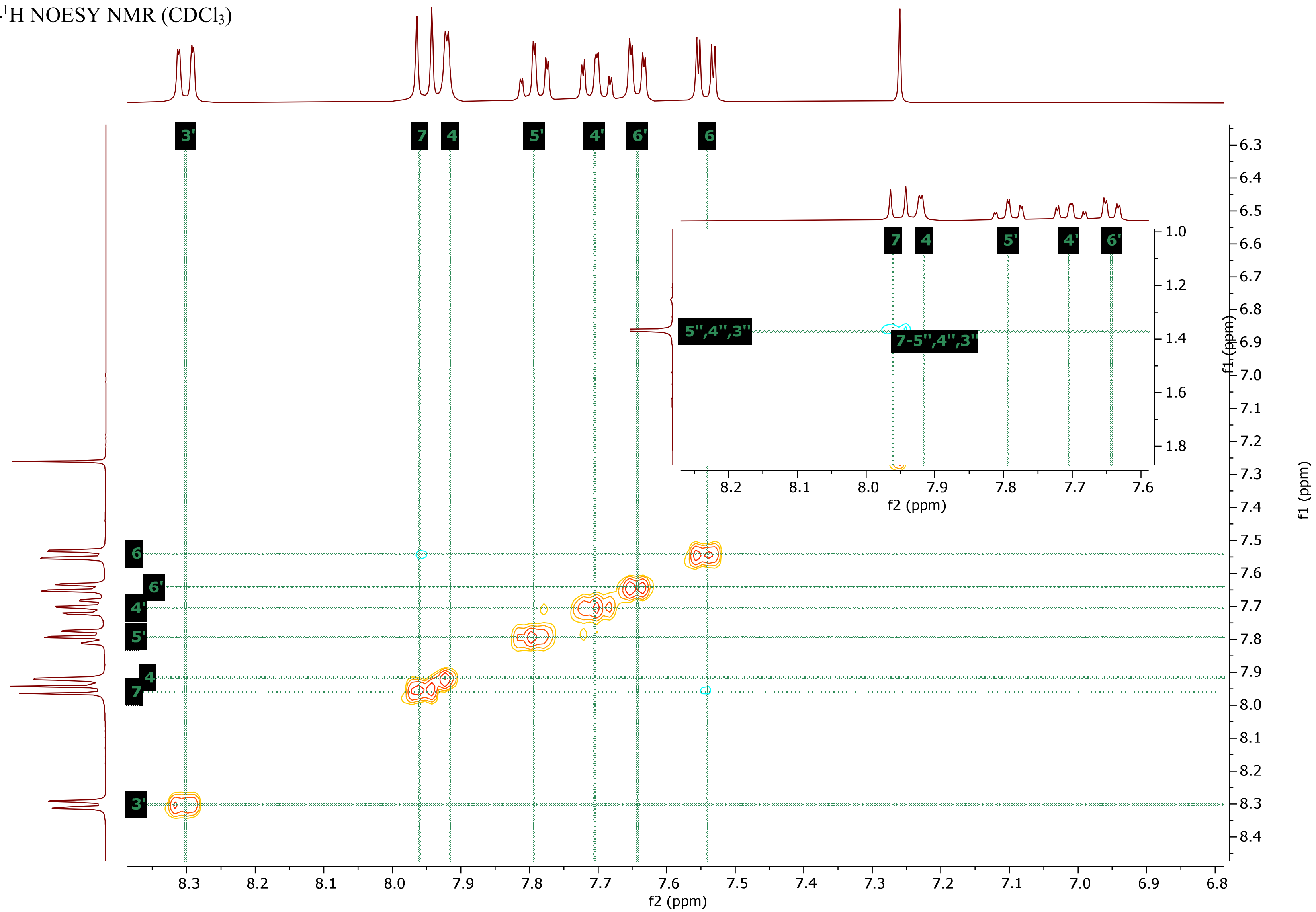

^1H-^{13}C HSQC NMR (DMSO-d_6)

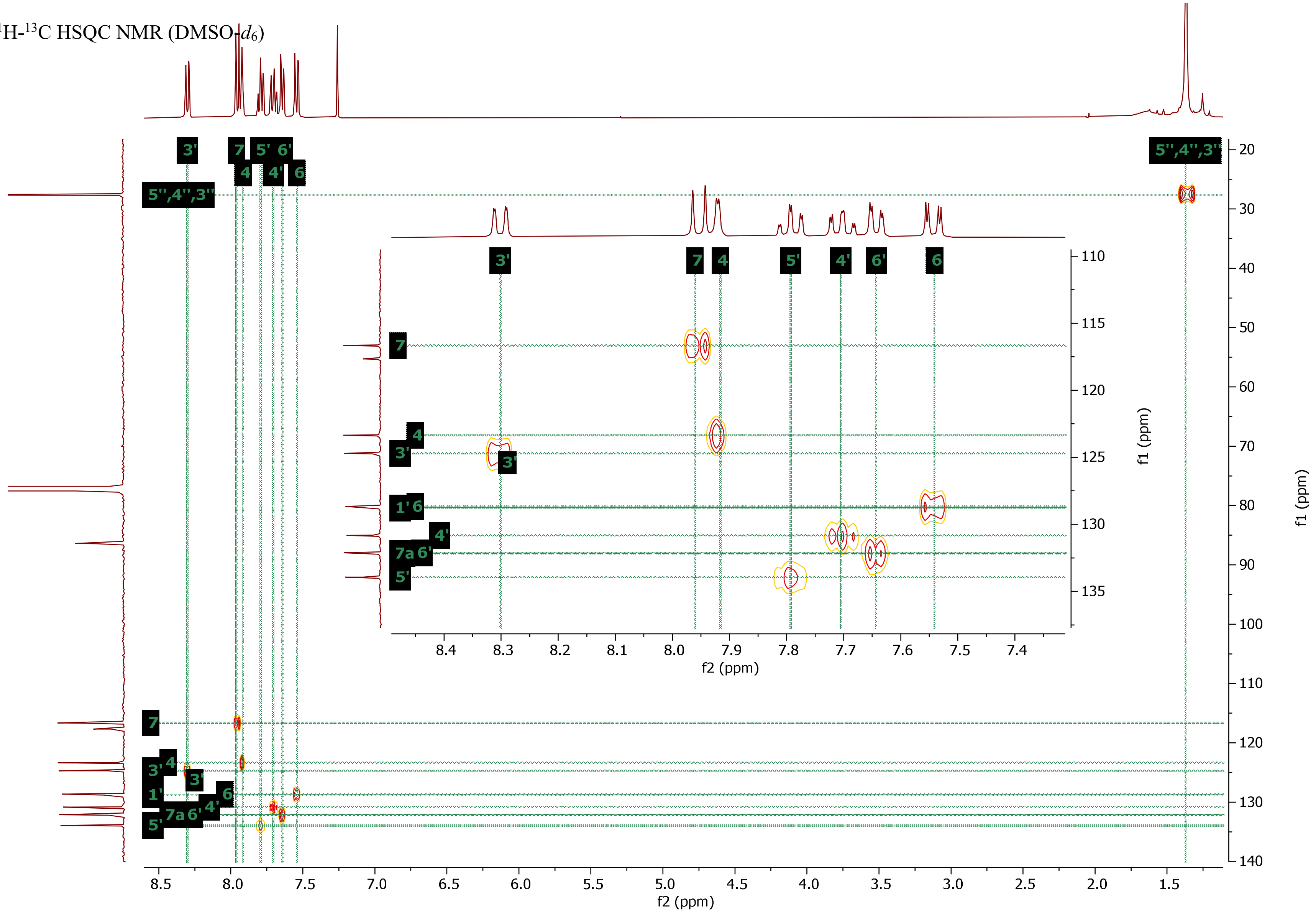

^{1}H-^{13}C HMBC NMR (DMSO-d_6)

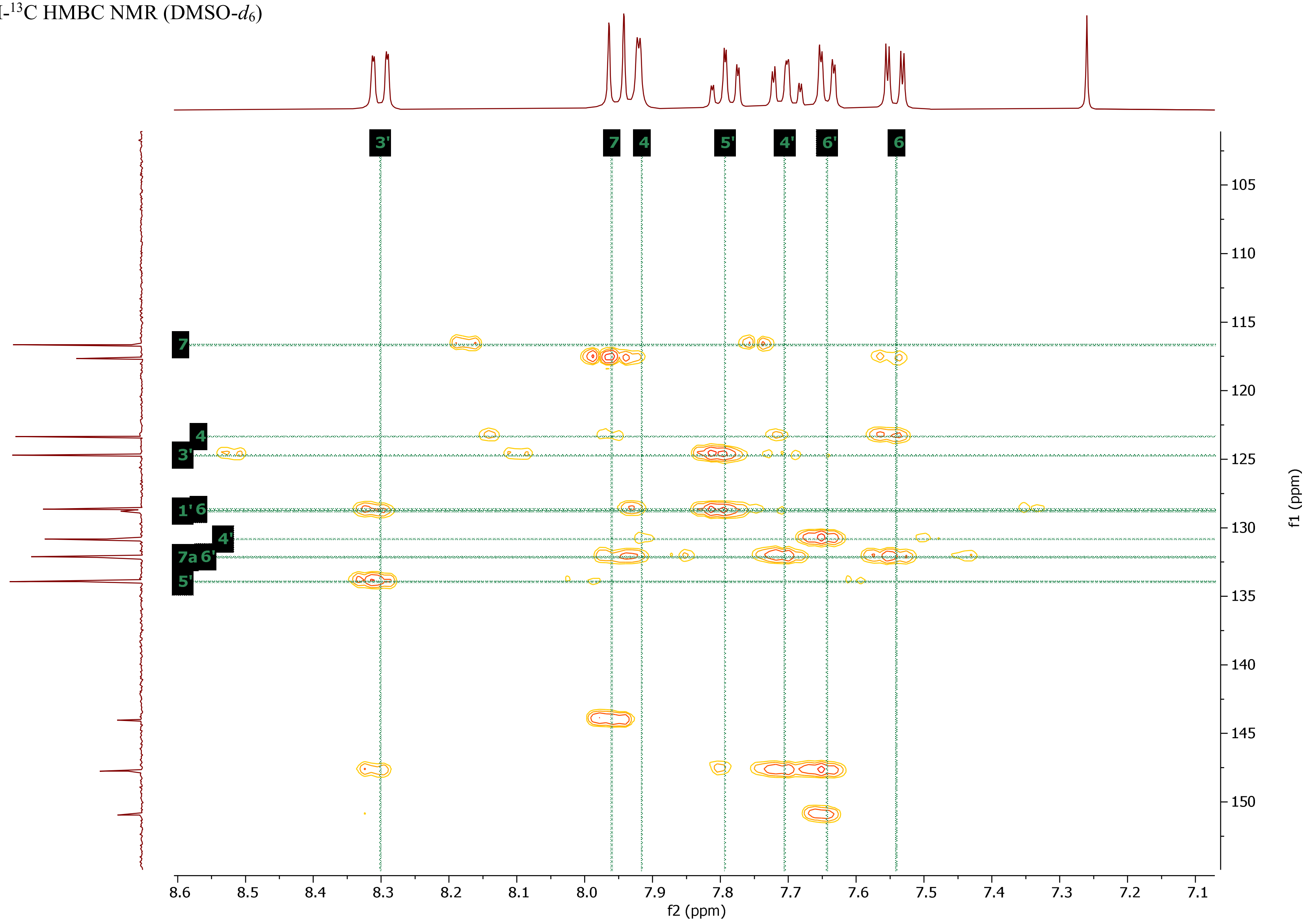

1-boc-6-bromo-2-(nitrophen-2-yl)-1H-benzimidazole **(3b)**

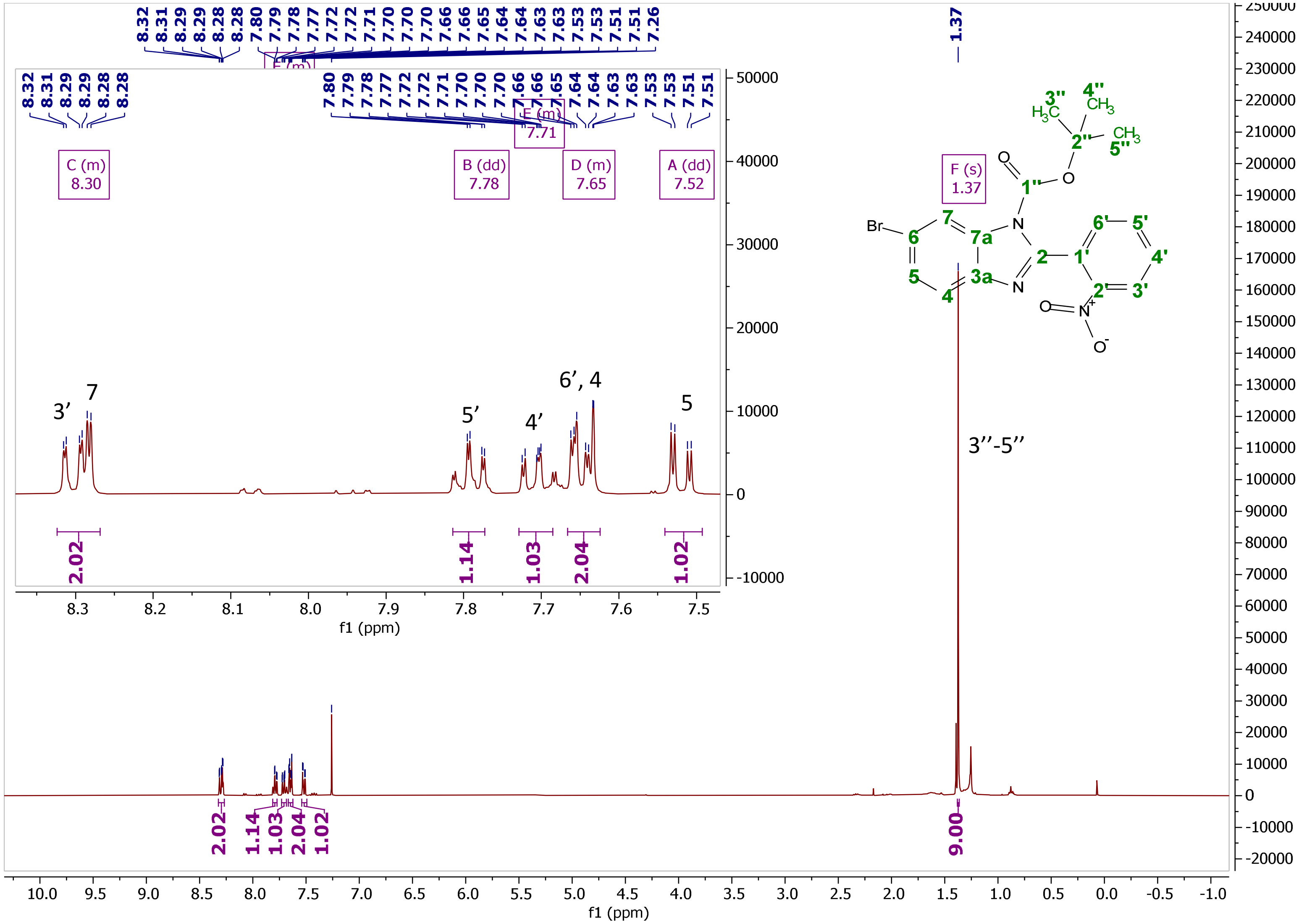

$^{13}C\{^1H\}$ NMR (101 MHz, $CDCl_3$)

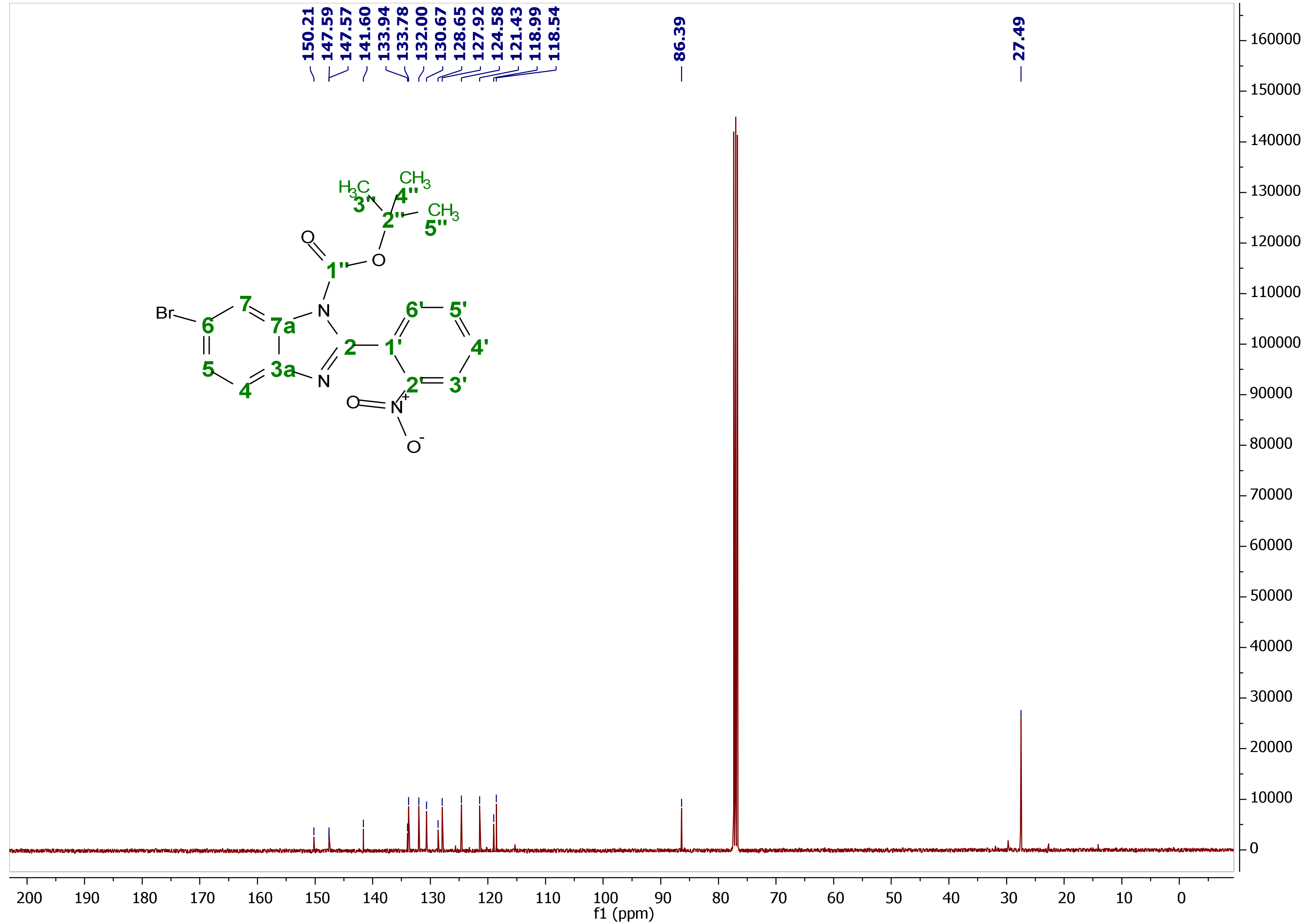

^1H-^1H NOESY NMR (400 MHz, $CDCl_3$)

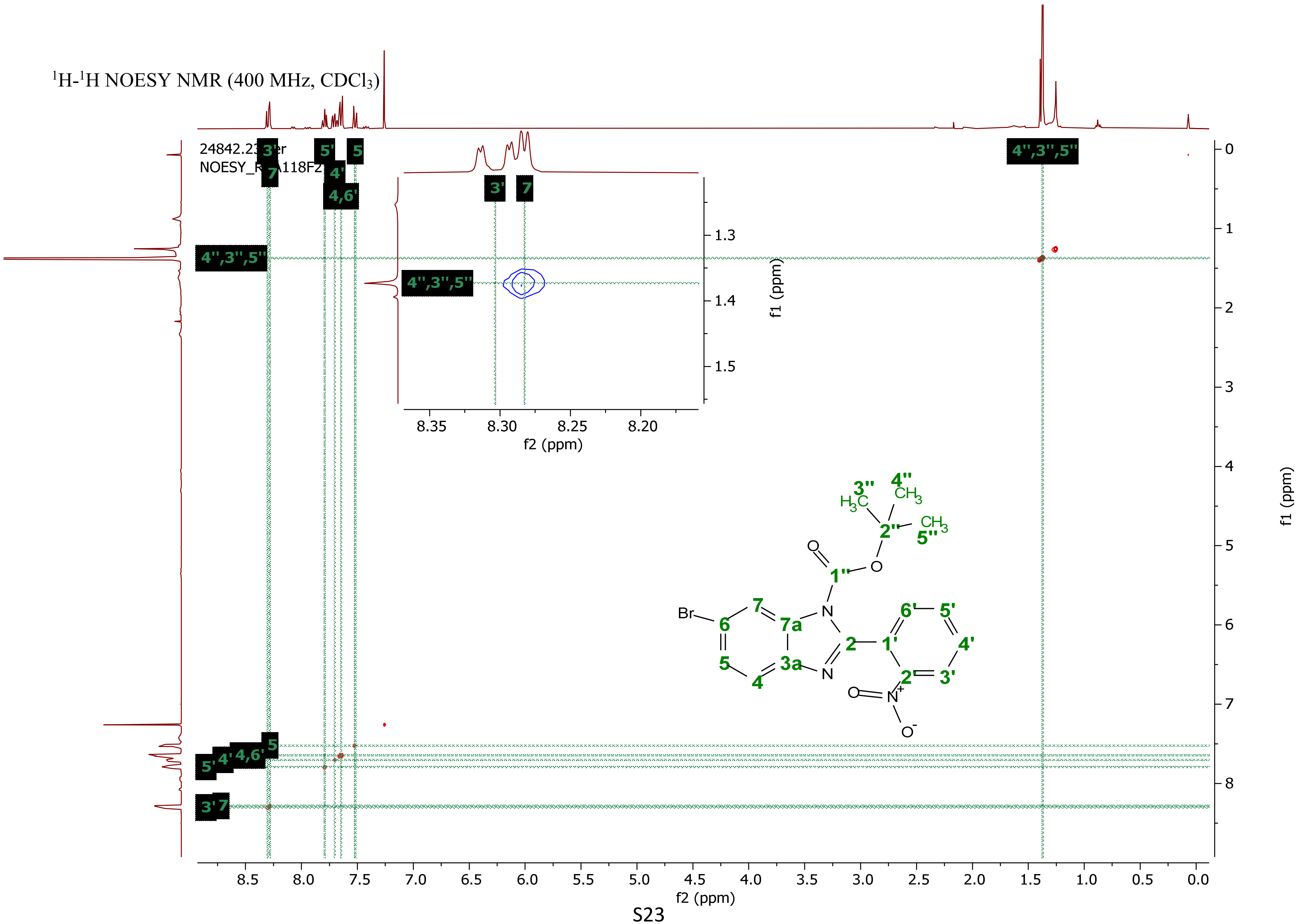

HRMS (ESI-TOF):

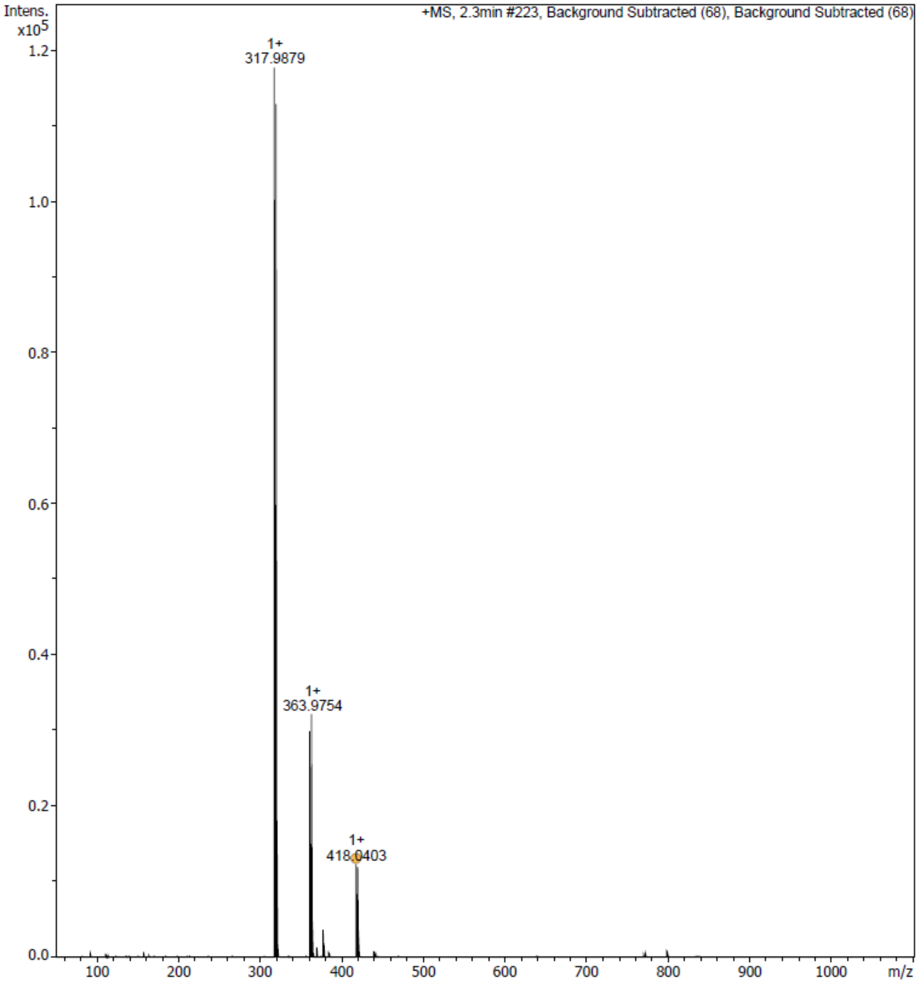

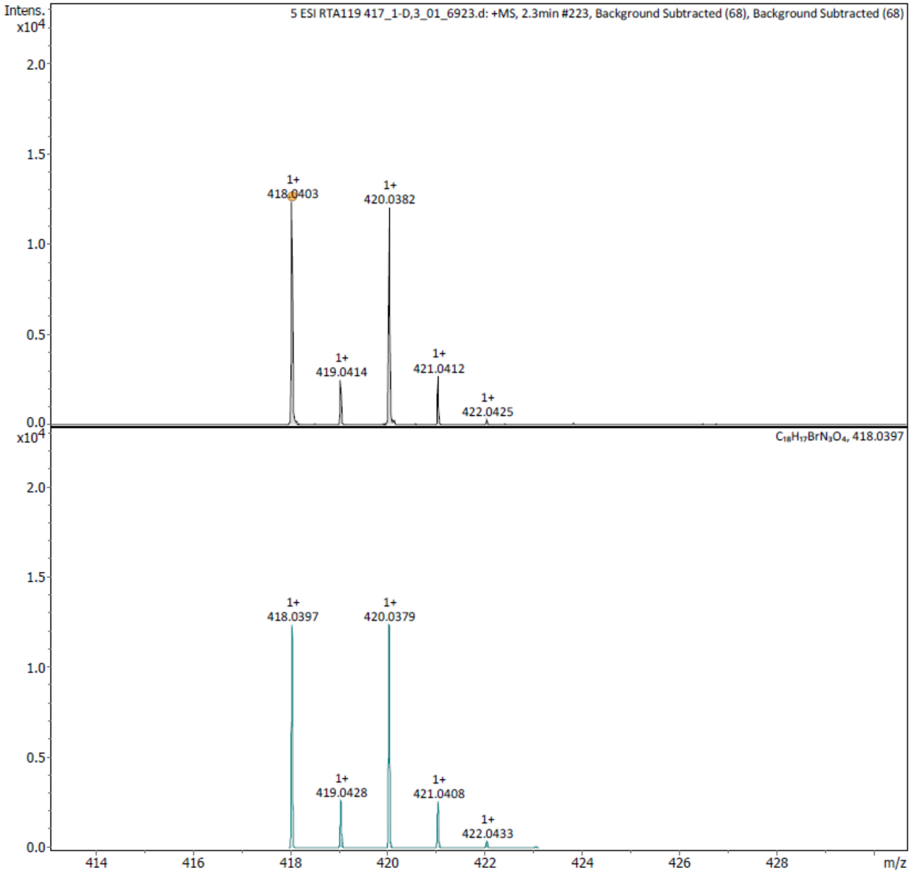

Meas. m/z	#	Ion Formula	Score	m/z	err [mDa]	err [ppm]	mSigma	rdb	e⁻ Conf	N-Rule
418.0403	1	C18H17BrN3O4	100.00	418.0397	-0.6	-1.4	13.9	14.0	even	ok
	2	C20H19BrO5	87.74	418.0410	0.8	1.9	15.1	13.5	odd	ok
	3	C19H13BrN7	82.36	418.0410	0.8	1.8	18.4	19.0	even	ok

1-benzyl-5(6)-(3,4,5-trimetoxiphen-1-yl)-2-(nitrophen-2-yl)-1H-benzimidazole **(4)**

^{1}H NMR (400 MHz, $CDCl_3$)

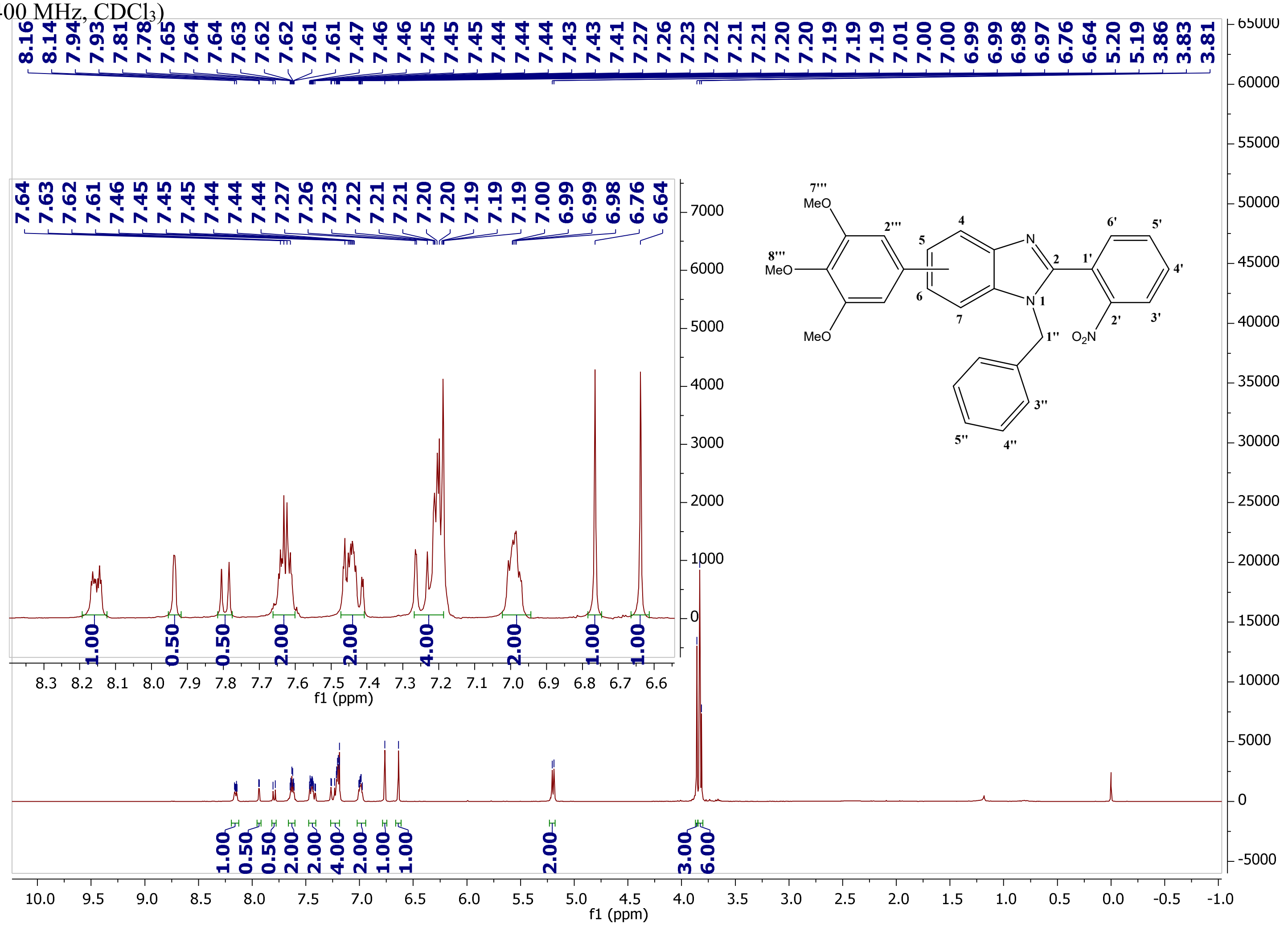

$^{13}C\{^{1}H\}$ NMR (101 MHz, $CDCl_3$)

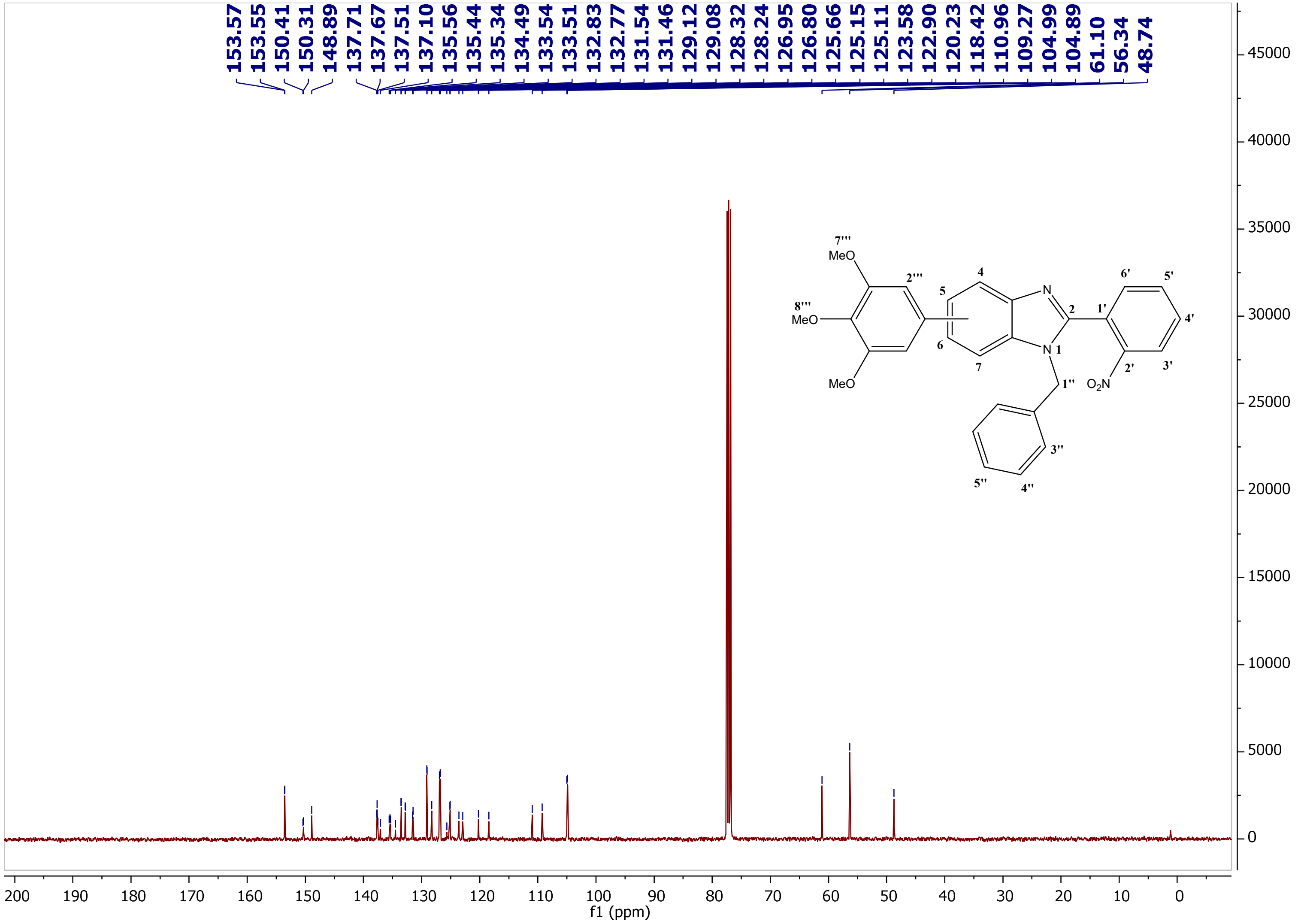

HRMS (ESI-TOF):

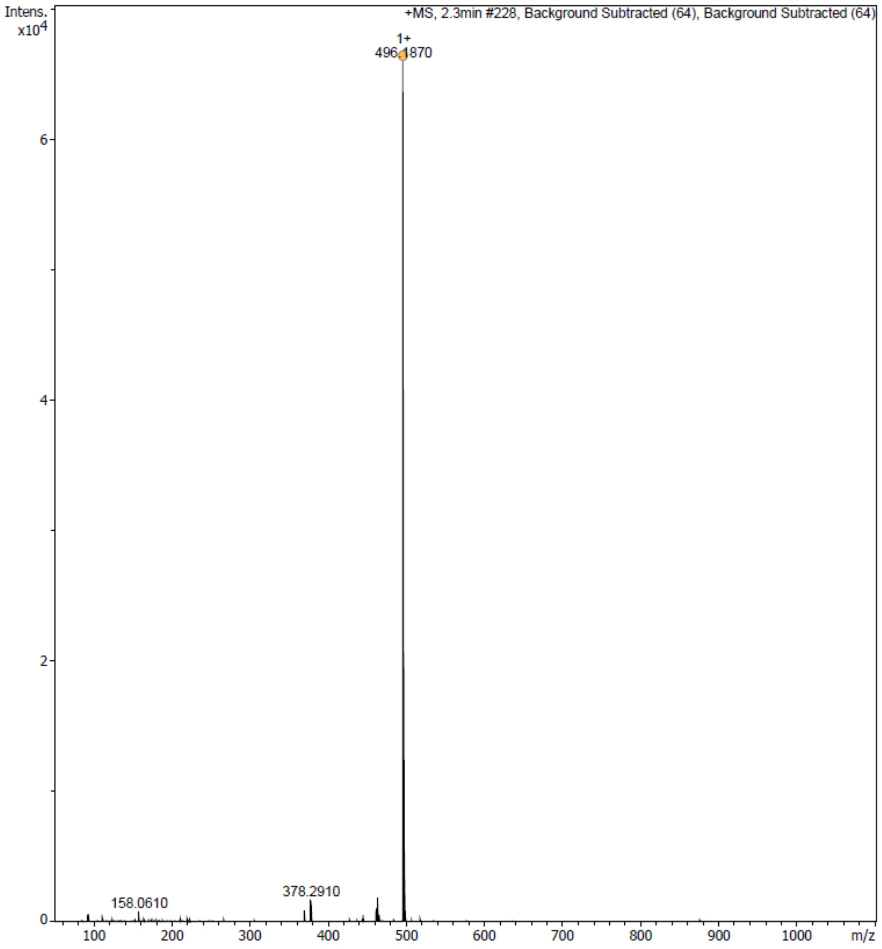
Intens.
x10^4
+MS, 2.3min #228, Background Subtracted (64), Background Subtracted (64)
1+
496.1870
378.2910
158.0610
m/z

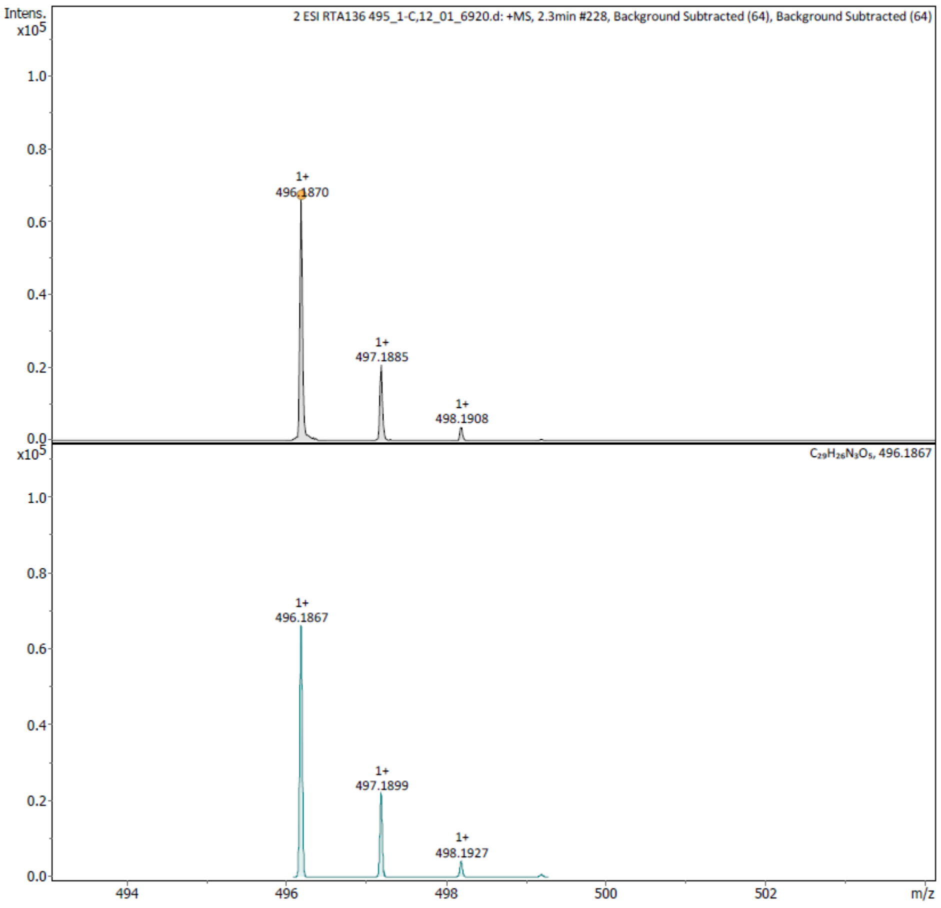
Intens.
x10^5
2 ESI RTA136 495_1-C,12_01_6920.d: +MS, 2.3min #228, Background Subtracted (64), Background Subtracted (64)
1+
496.1870
1+
497.1885
1+
498.1908
$C_{29}H_{26}N_3O_5$, 496.1867
1+
496.1867
1+
497.1899
1+
498.1927
m/z

1-benzyl-5(6)-(3-fluoro-4-(methoxycarbonyl)phen-1-yl)-2-(nitrophen-2-yl)-1H-benzimidazole **(5)**

^{1}H NMR (400 MHz, acetone-d_6)

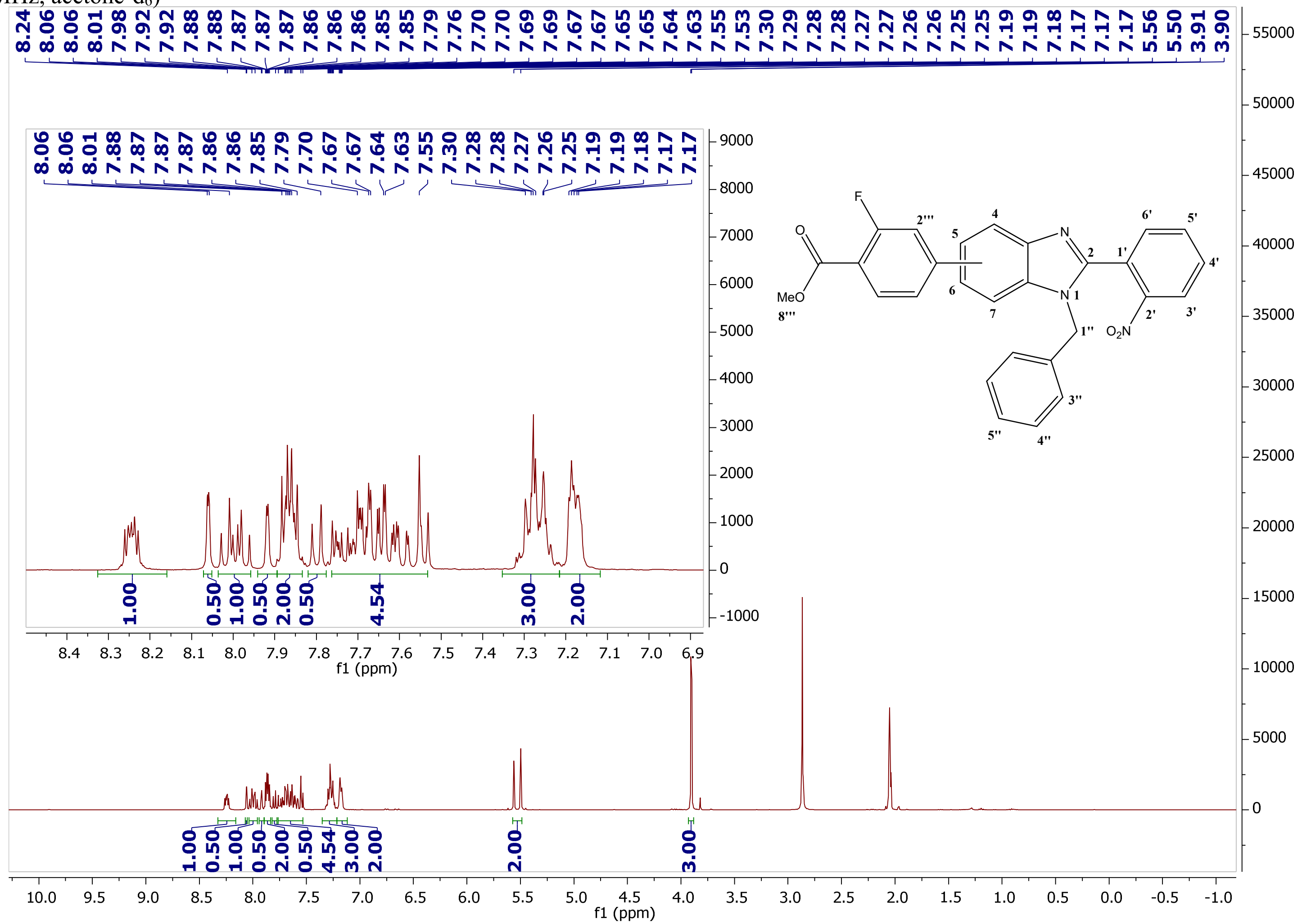

^{13}C NMR (101 MHz, acetone-d_6)

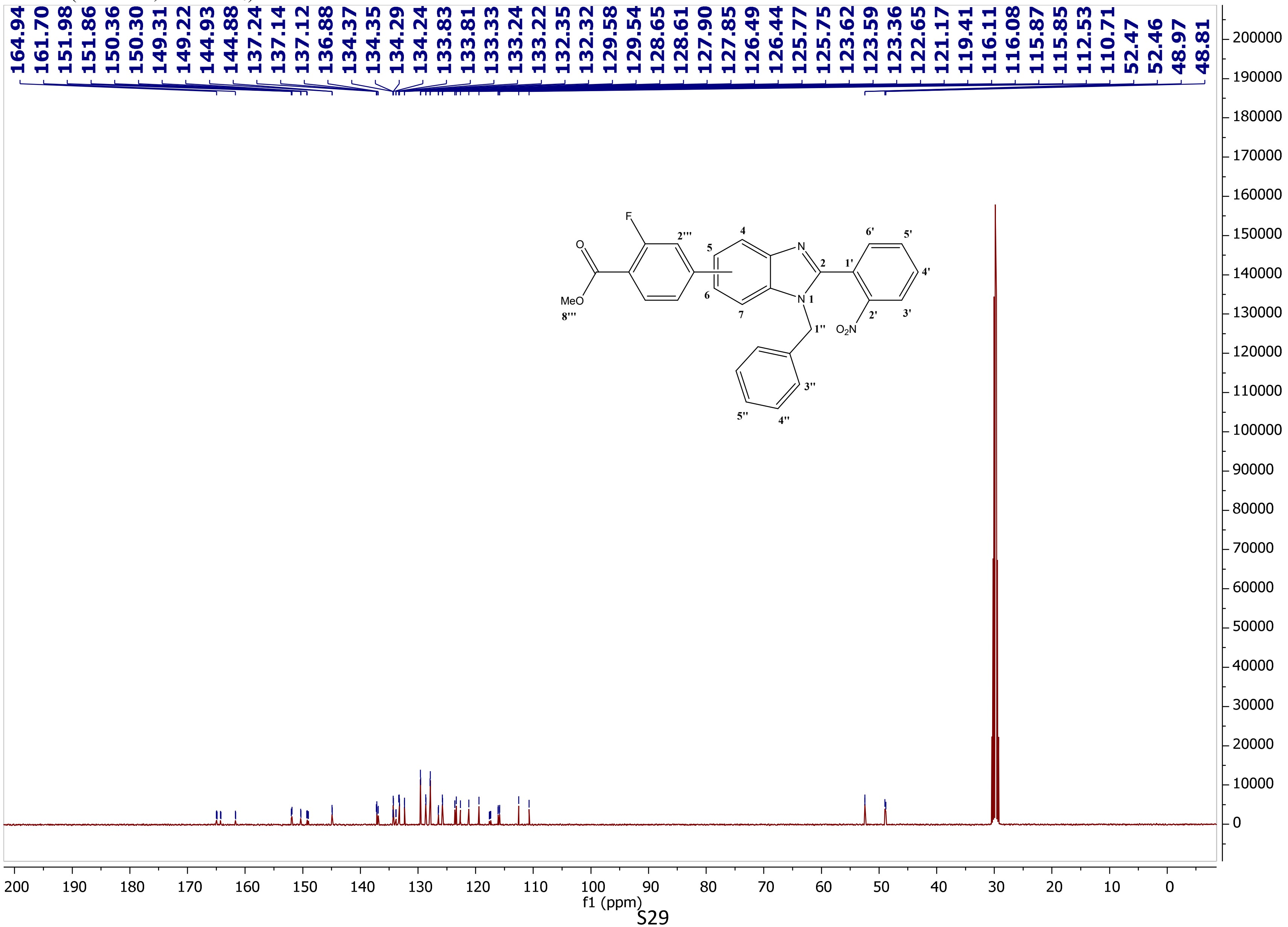

HRMS (ESI-TOF):

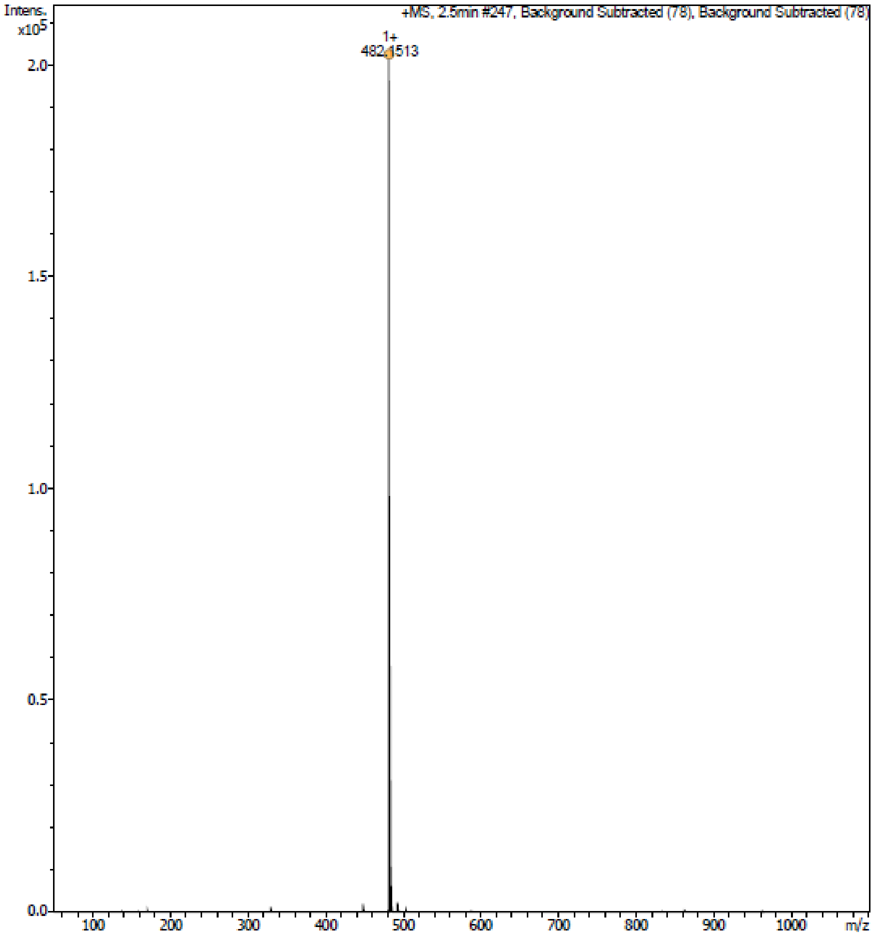

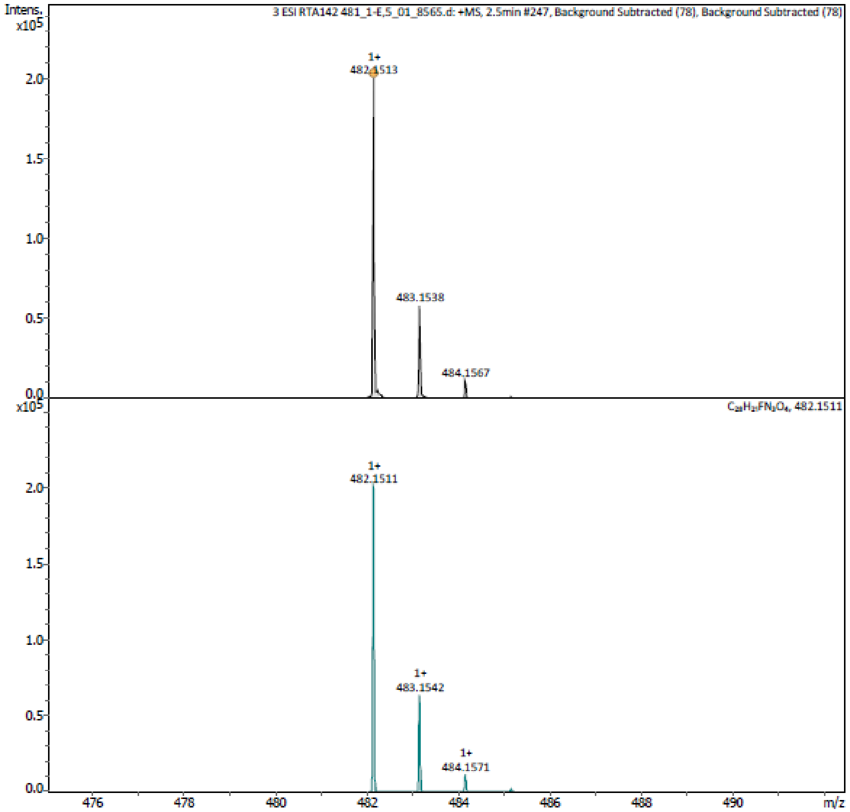

Meas. m/z	#	Ion Formula	Score	m/z	err [mDa]	err [ppm]	mSigma	rdb	e⁻ Conf	N-Rule
482.1513	1	C26H19FN6O3	38.68	482.1497	-1.6	-3.3	10.1	20.5	odd	ok
	2	C28H21FN3O4	100.00	482.1511	-0.2	-0.5	14.4	20.0	even	ok
	3	C19H14N16O	28.52	482.1531	1.8	3.8	17.0	21.5	odd	ok
	4	C30H23FO5	56.02	482.1524	1.1	2.3	20.4	19.5	odd	ok
	5	C20H20N9O6	25.90	482.1531	1.8	3.8	21.4	16.0	even	ok

1-benzyl-N-(4-(methylsulfonyl)phenyl)-2-(nitrophen-2-yl)-1-benzimidazol-5(6)-amine **(6)**

^{1}H NMR (400 MHz, acetone-d$_6$)

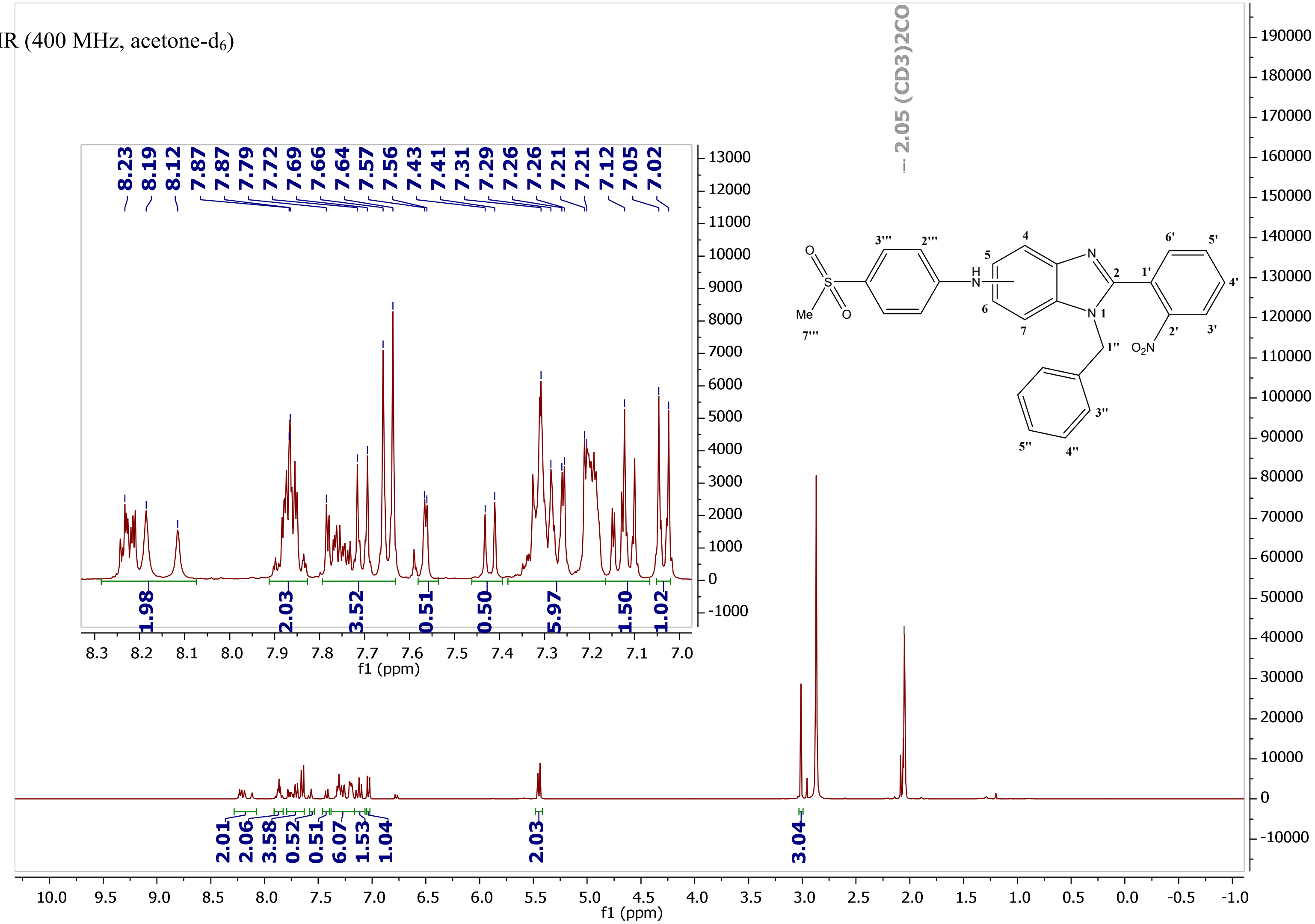

$^{13}C\{^{1}H\}$ NMR (101 MHz, acetone-d$_6$)

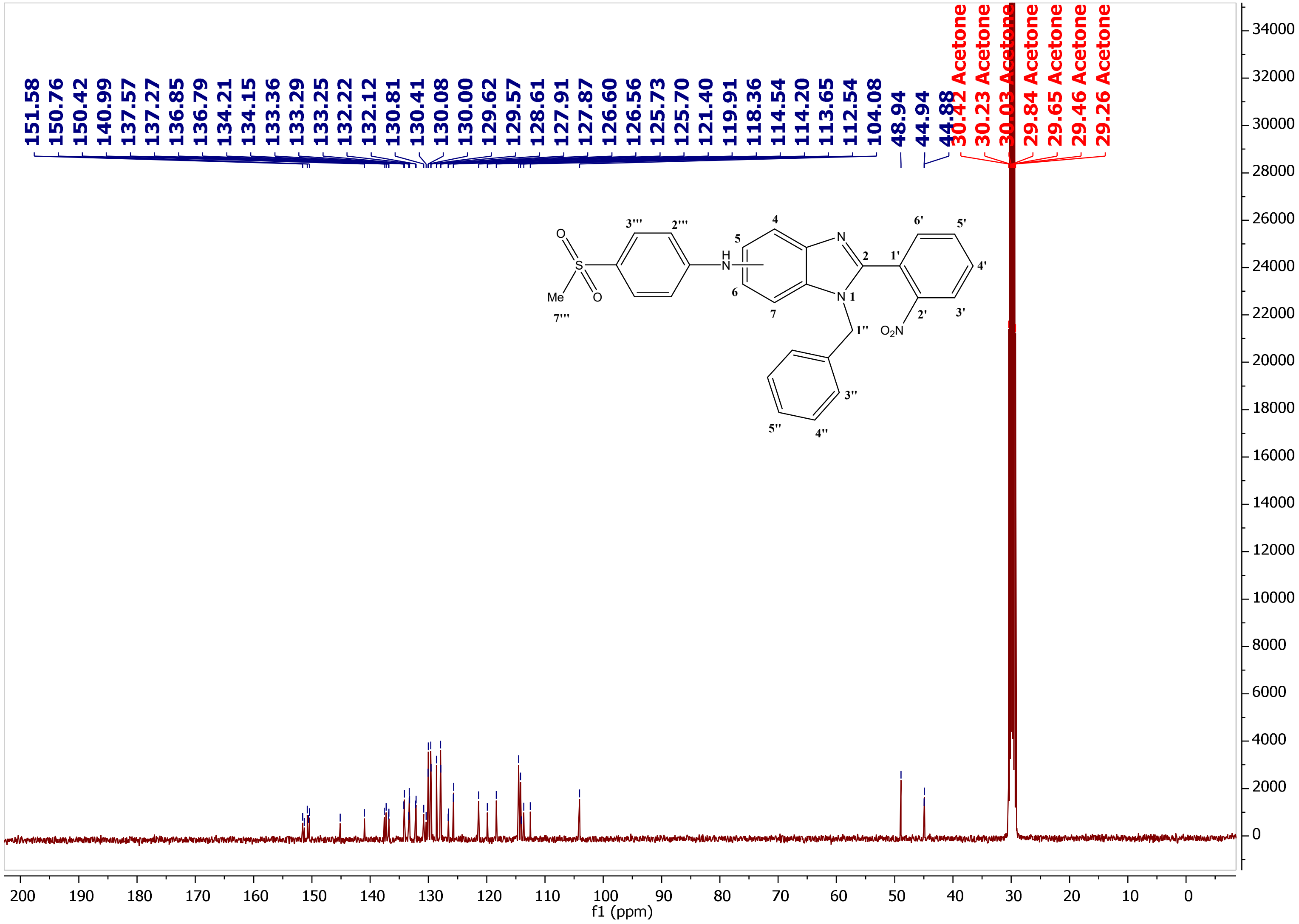

HRMS (ESI-TOF):

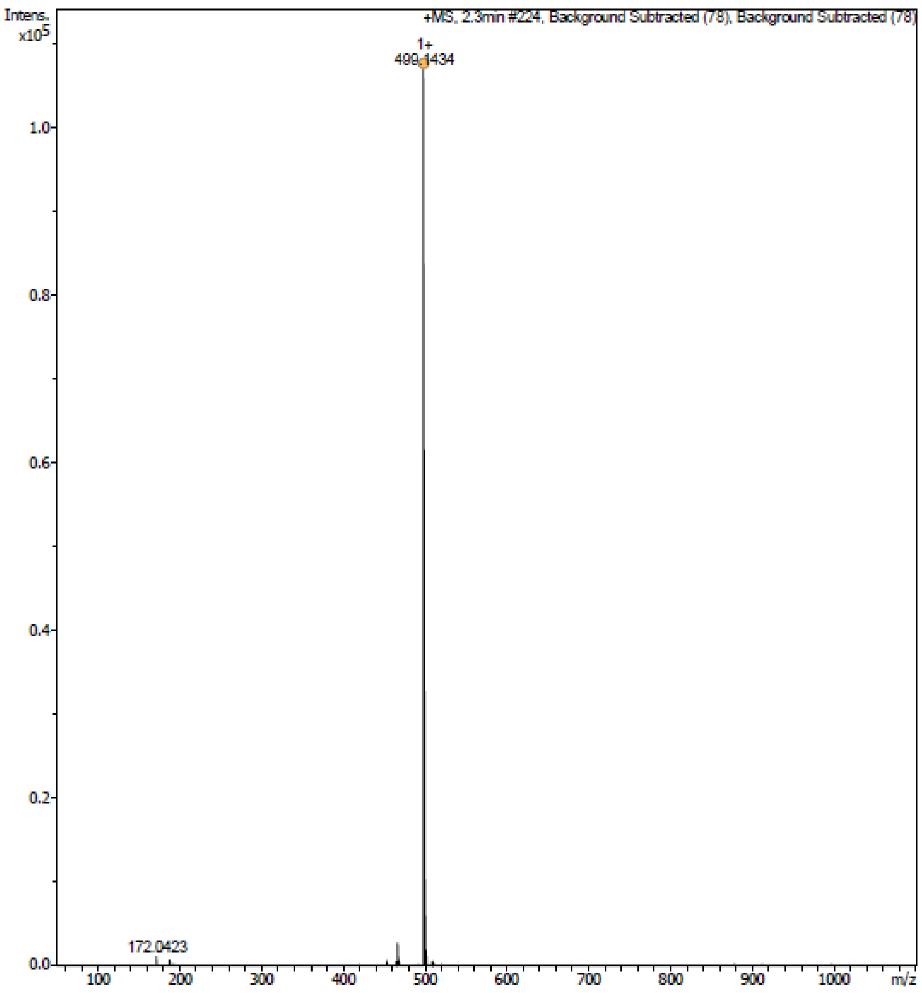

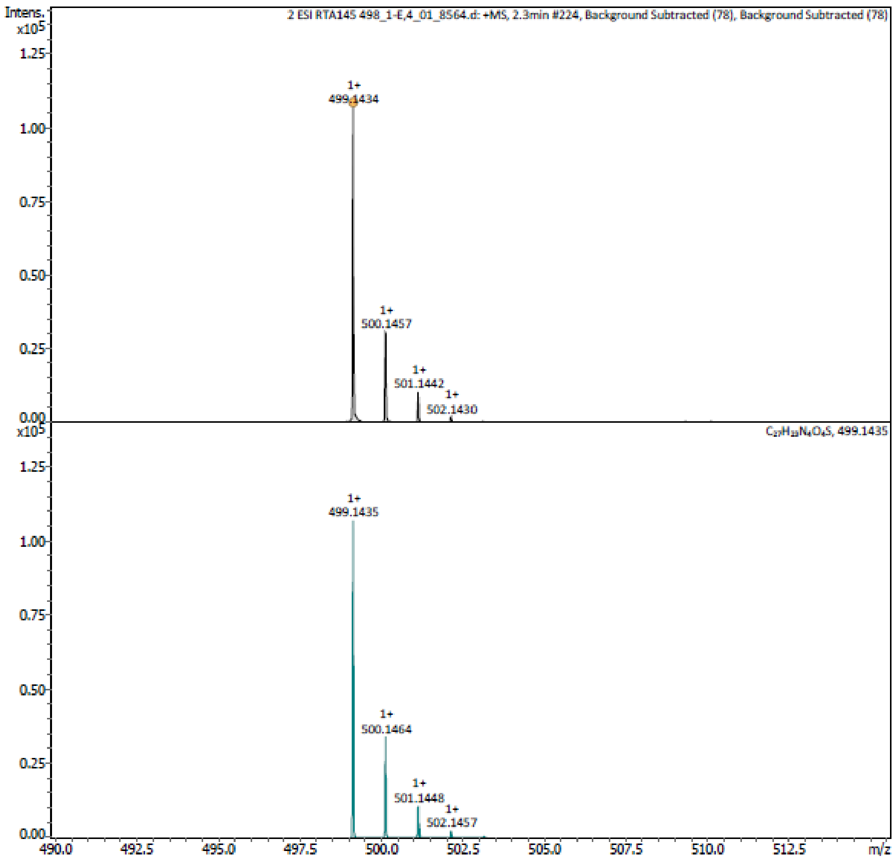

Meas. m/z	#	Ion Formula	Score	m/z	err [mDa]	err [ppm]	mSigma	rdb	e¯ Conf	N-Rule
499.1434	1	C26H27O8S	62.59	499.1421	-1.3	-2.7	5.7	16.0	even	ok
	2	C25H21N7O3S	57.05	499.1421	-1.3	-2.7	10.7	21.5	odd	ok
	3	C27H23N4O4S	100.00	499.1435	0.0	0.0	16.6	21.0	even	ok
	4	C29H25NO5S	43.92	499.1448	1.4	2.7	22.7	20.5	odd	ok
	5	C28H19N8S	38.90	499.1448	1.3	2.7	28.1	26.0	even	ok

1-benzyl-N-(3-(methylsulfonyl)phenyl)-2-(nitrophen-2-yl)-1H-benzimidazol-5(6)-amine **(7)**

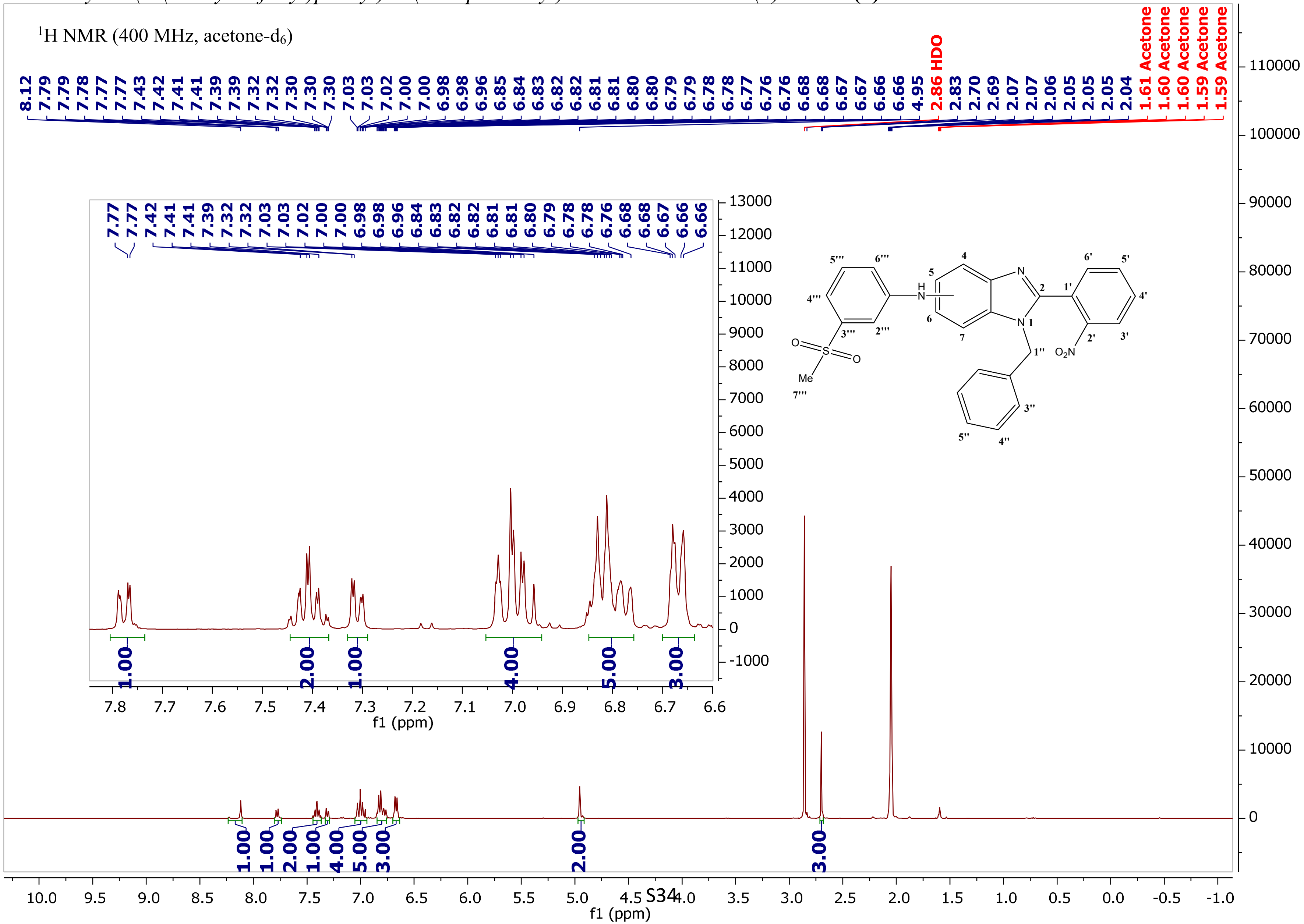

$^{13}C\{^1H\}$ NMR (101 MHz, acetone-d_6)

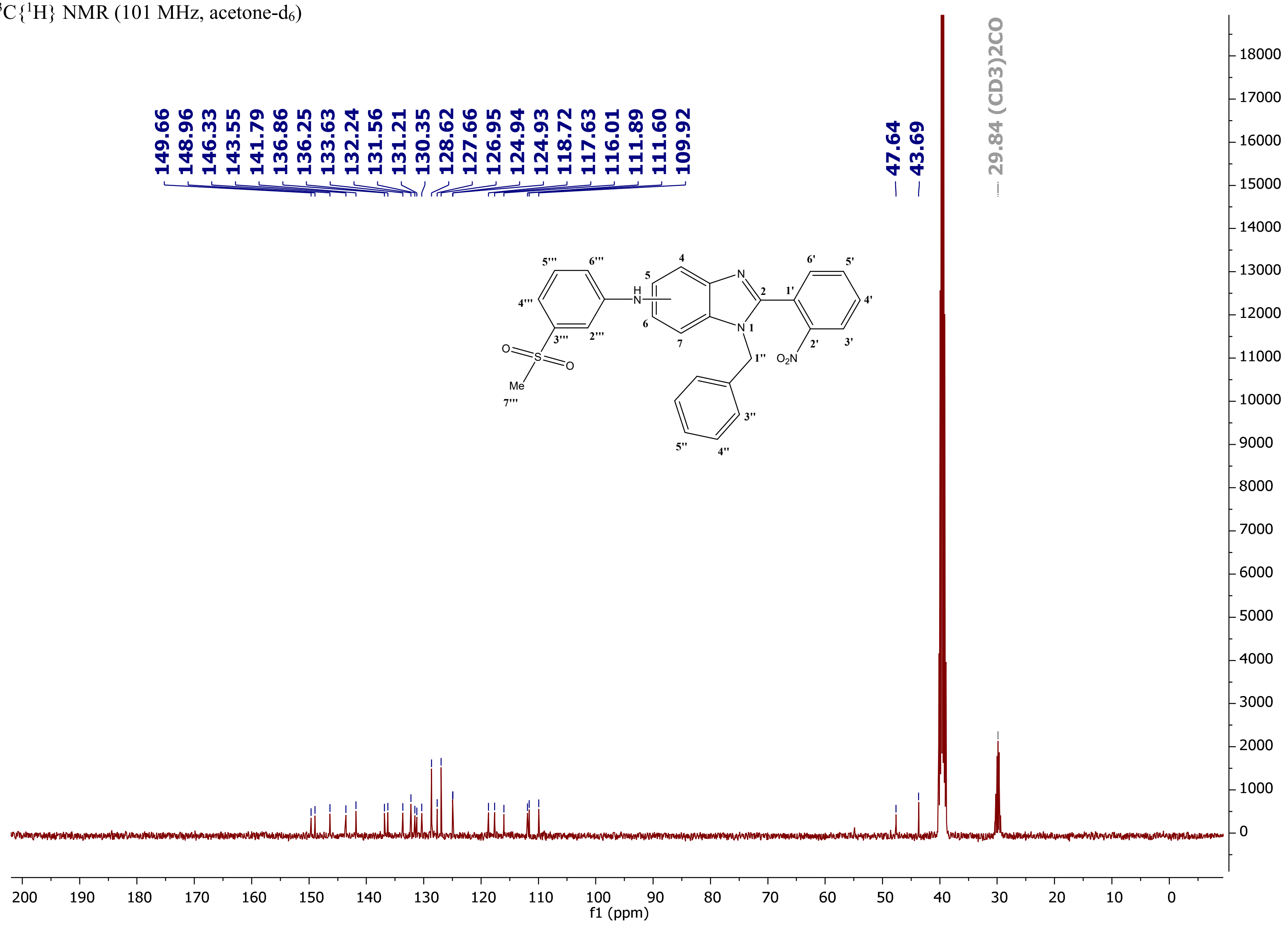

HRMS (ESI-TOF):

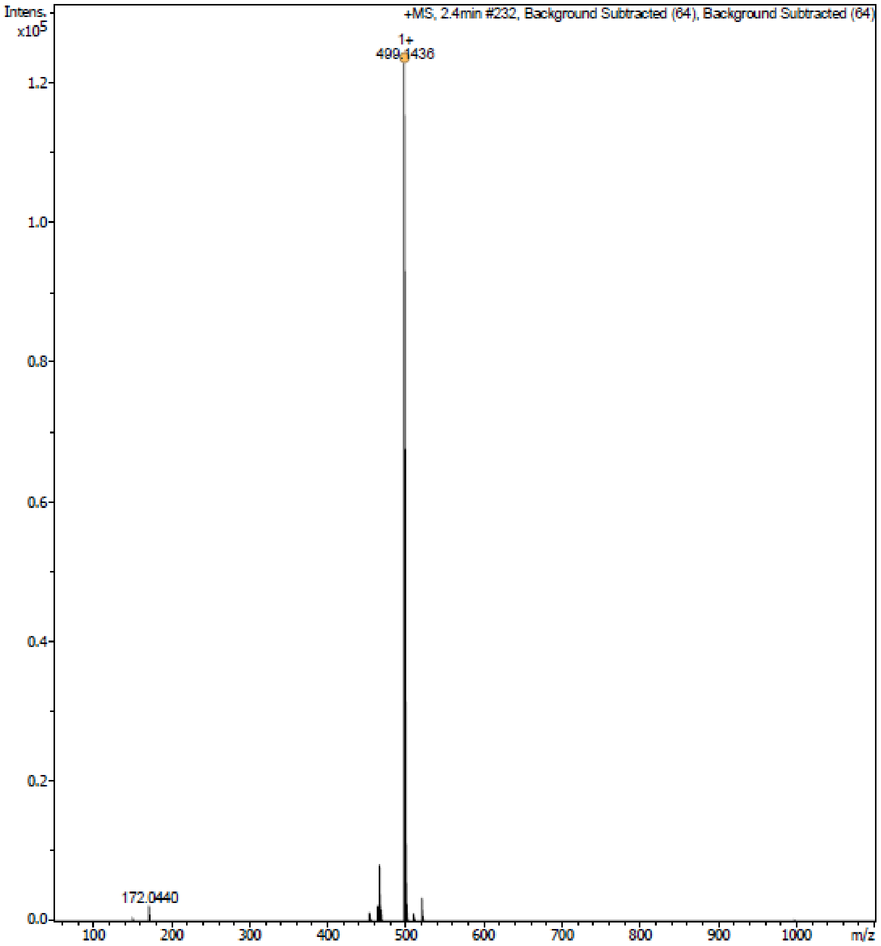

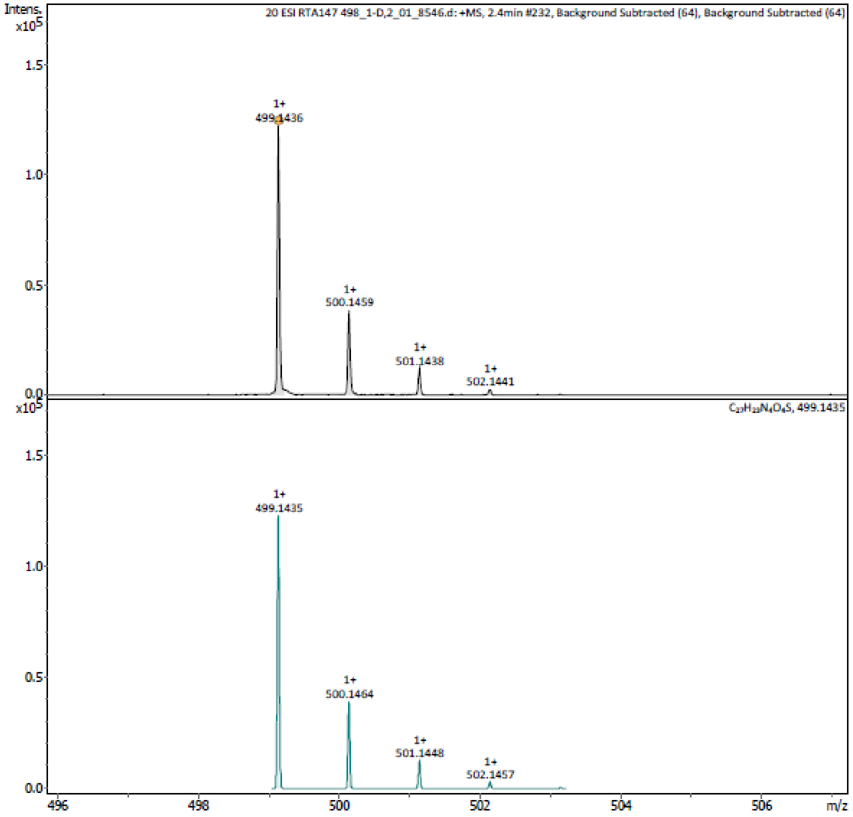

Meas. m/z	#	Ion Formula	Score	m/z	err [mDa]	err [ppm]	mSigma	rdb	e⁻ Conf	N-Rule
499.1436	1	$C_{25}H_{21}N_7O_3S$	49.21	499.1421	-1.5	-3.1	2.8	21.5	odd	ok
	2	$C_{27}H_{23}N_4O_4S$	100.00	499.1435	-0.2	-0.4	4.2	21.0	even	ok
	3	$C_{26}H_{27}O_8S$	43.82	499.1421	-1.5	-3.1	9.6	16.0	even	ok
	4	$C_{29}H_{25}NO_5S$	54.40	499.1448	1.2	2.3	10.5	20.5	odd	ok
	5	$C_{28}H_{19}N_8S$	50.13	499.1448	1.2	2.3	14.9	26.0	even	ok

1-boc-5-(3,4,5-trimetoxiphen-1-yl)-2-(nitrophen-2-yl)-1H-benzimidazole **(8a)**

^{1}H NMR (400 MHz, acetone-d$_6$)

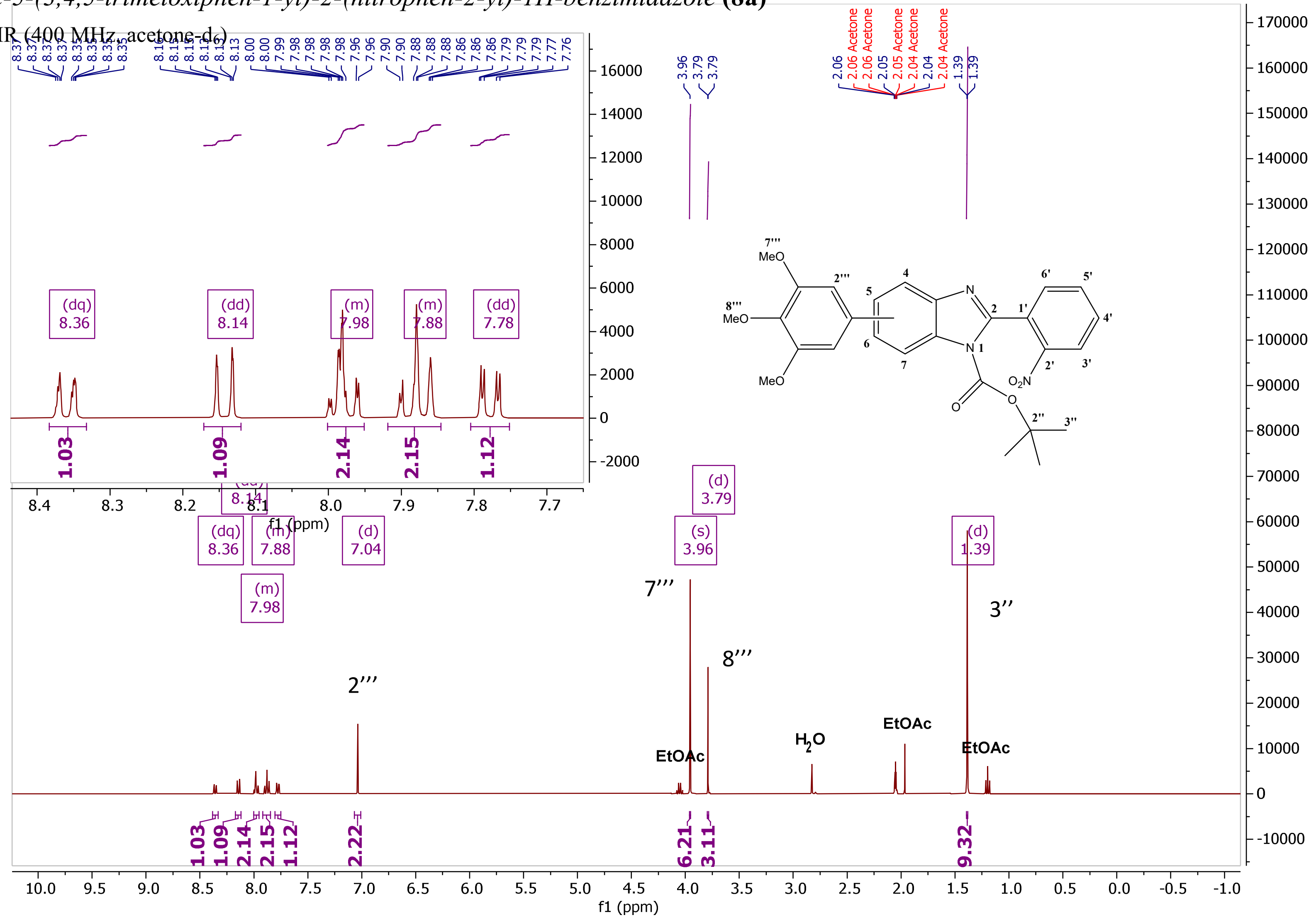

$^{13}C\{^{1}H\}$ NMR (101 MHz, acetone-d_6)

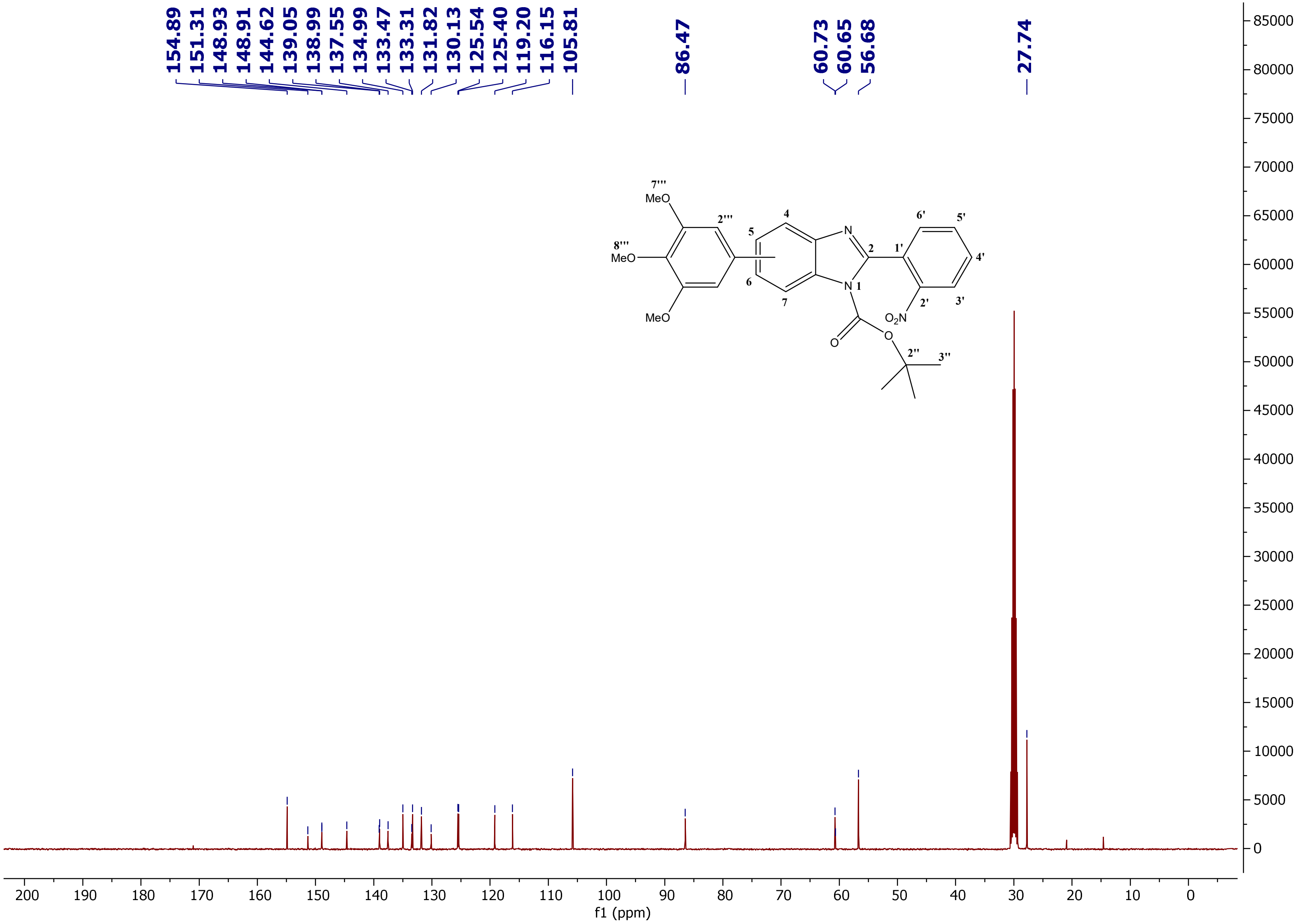

HRMS (ESI-TOF):

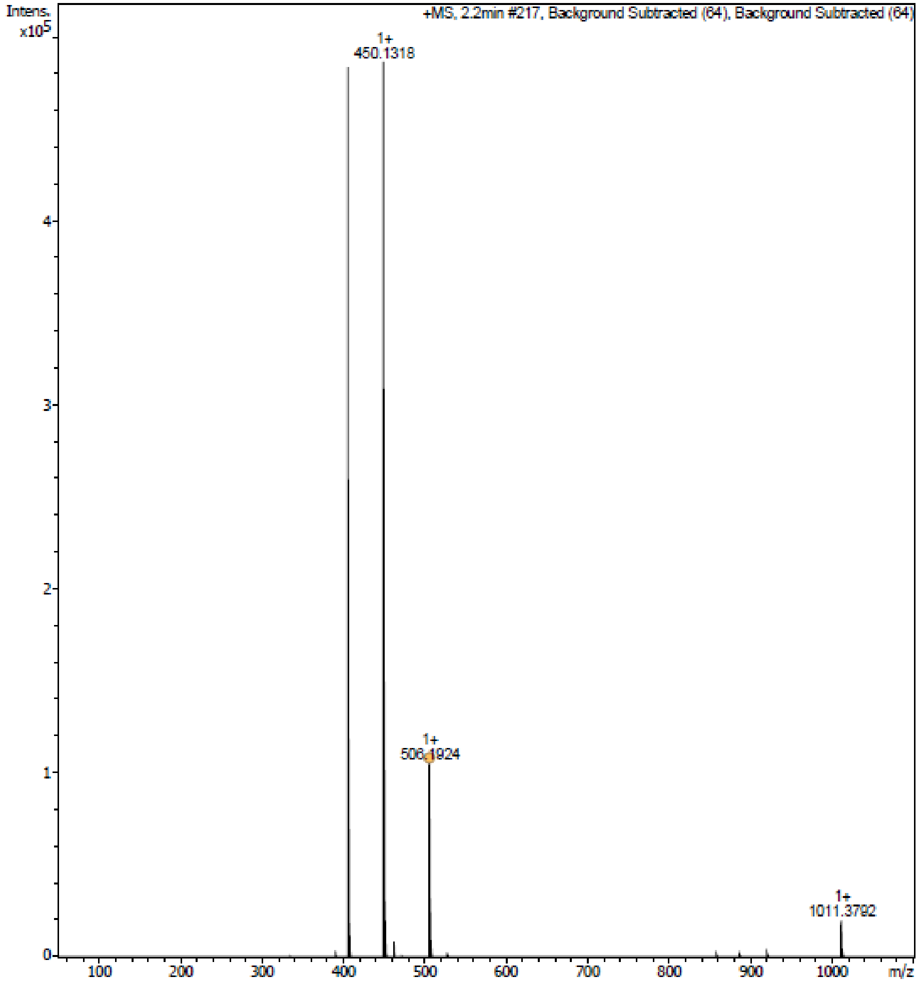

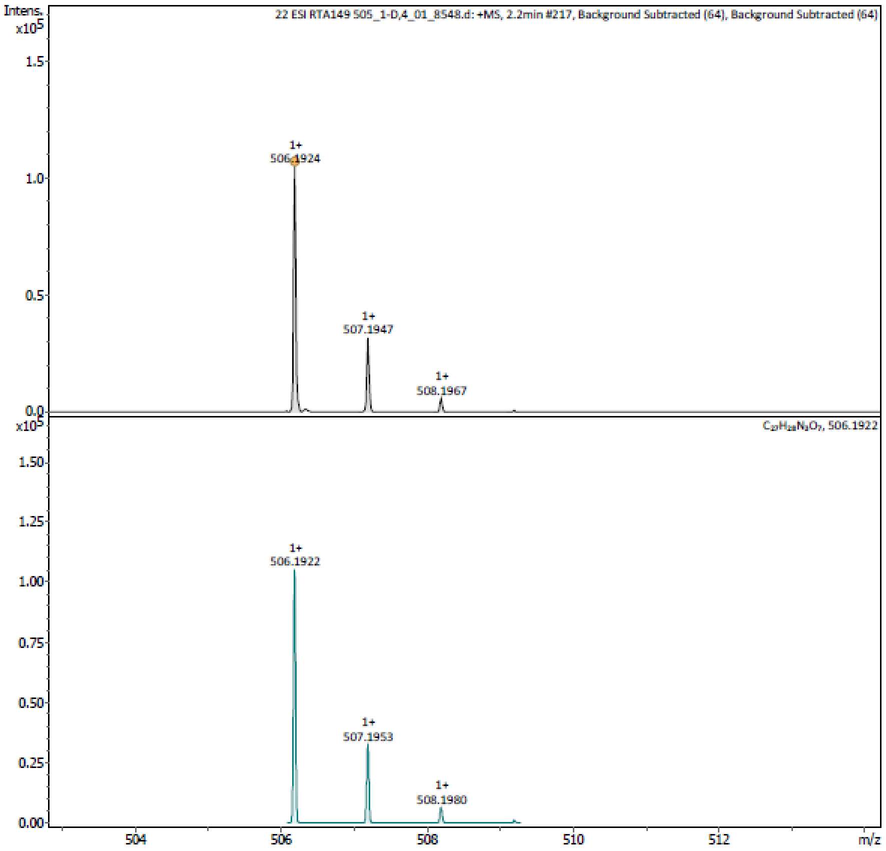

Meas. m/z	#	Ion Formula	Score	m/z	err [mDa]	err [ppm]	mSigma	rdb	e⁻ Conf	N-Rule
506.1924	1	C25H26N6O6	48.47	506.1908	-1.6	-3.1	2.5	16.5	odd	ok
	2	C27H28N3O7	100.00	506.1922	-0.3	-0.5	4.0	16.0	even	ok
	3	C24H20N13O	34.01	506.1908	-1.6	-3.2	6.2	22.0	even	ok
	4	C29H30O8	58.10	506.1935	1.1	2.2	10.3	15.5	odd	ok
	5	C26H22N10O2	66.19	506.1922	-0.3	-0.5	10.9	21.5	odd	ok

1-boc-5-(3-fluoro-4-(methoxycarbonyl)phen-1-yl)-2-(nitrophen-2-yl)-benzimidazole **(9a)**

^{1}H NMR (400 MHz, acetone-d6)

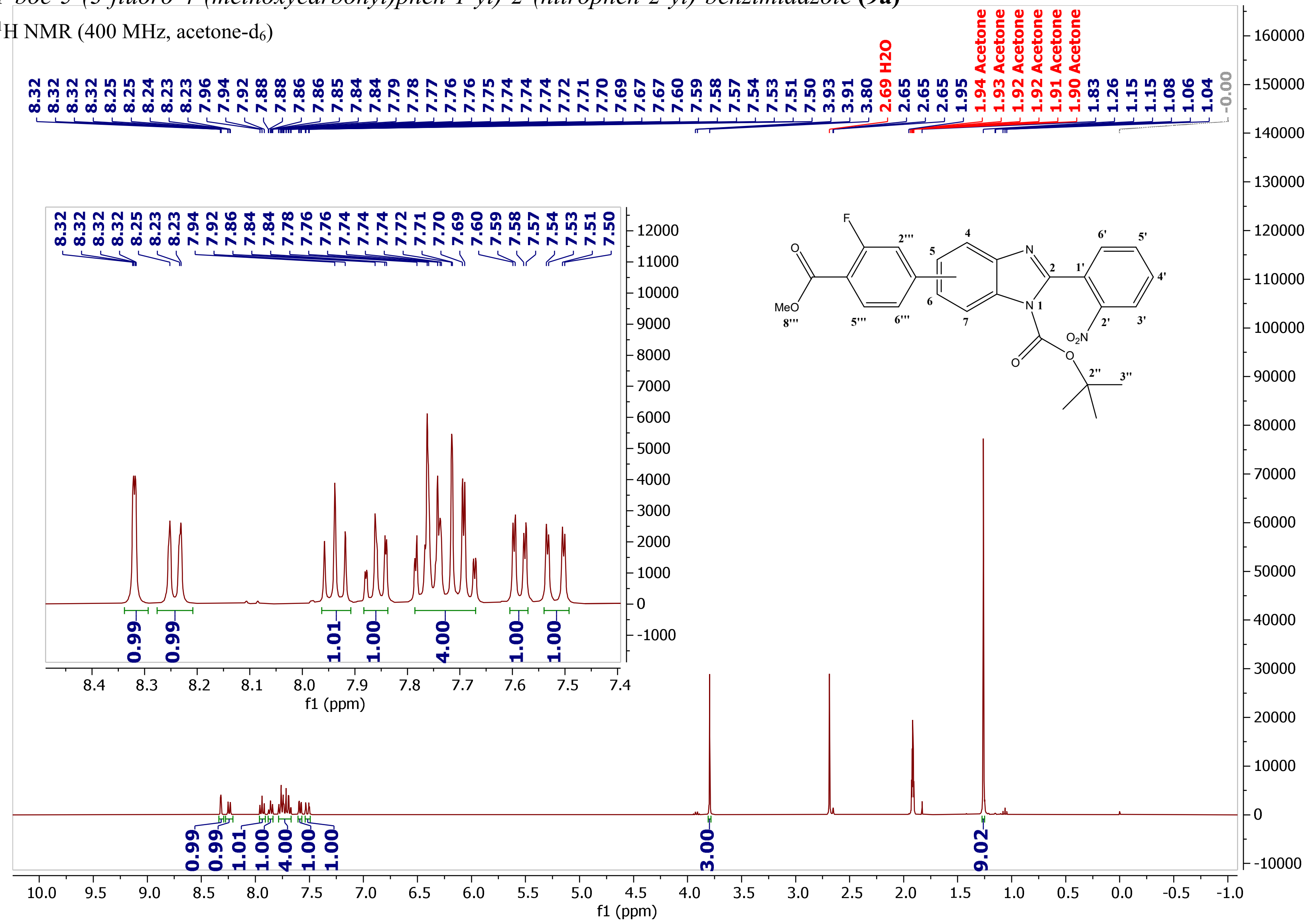

$^{13}C\{^1H\}$ NMR (101 MHz, acetone-d_6)

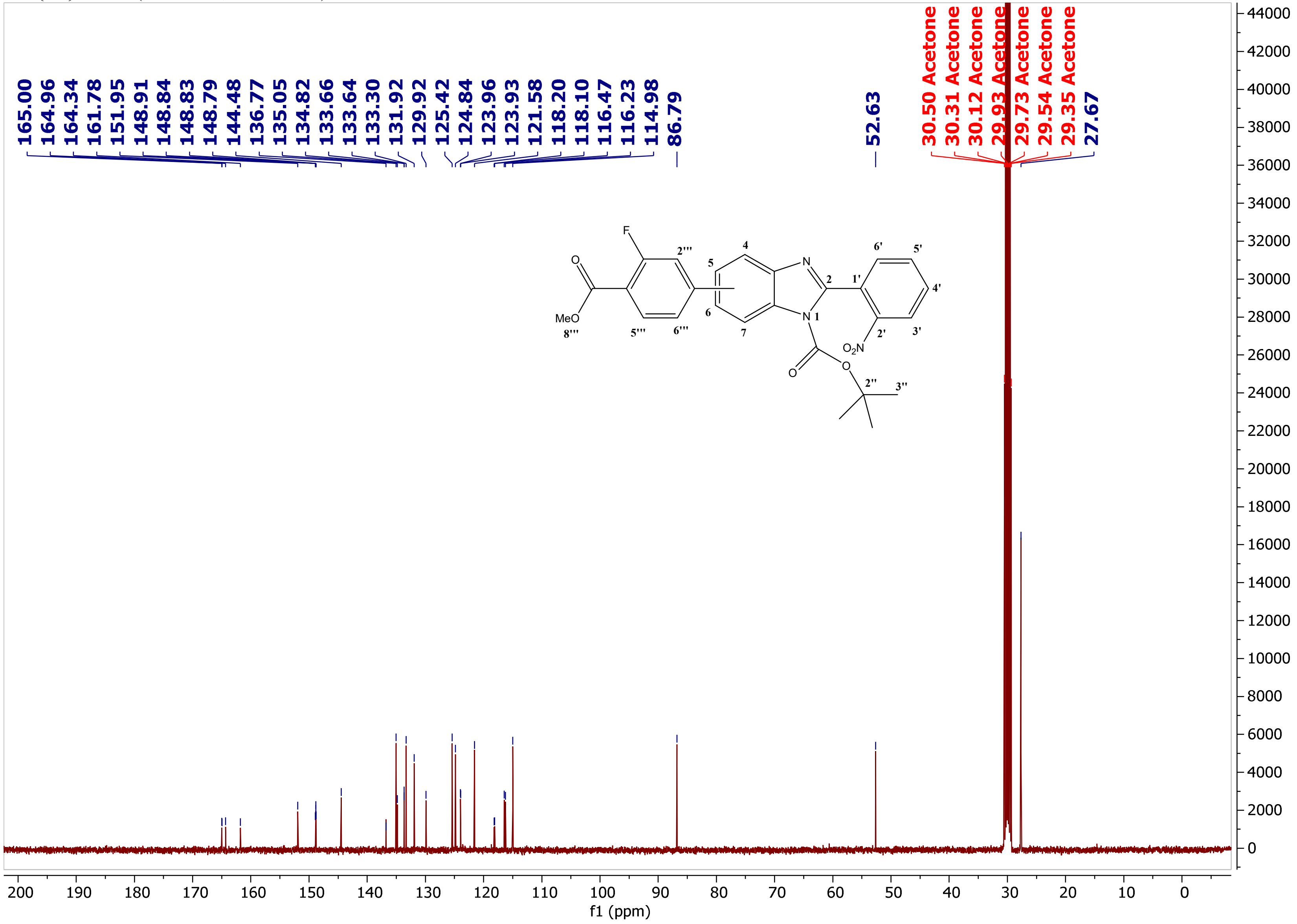

HRMS (ESI-TOF):

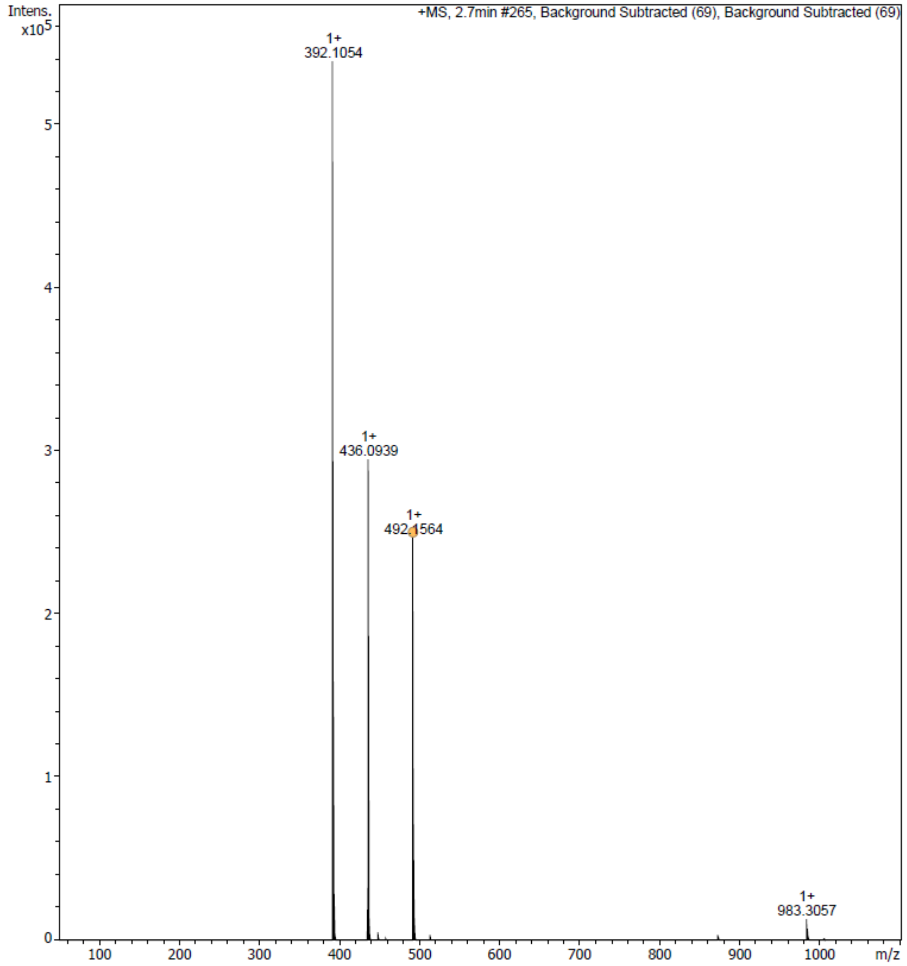

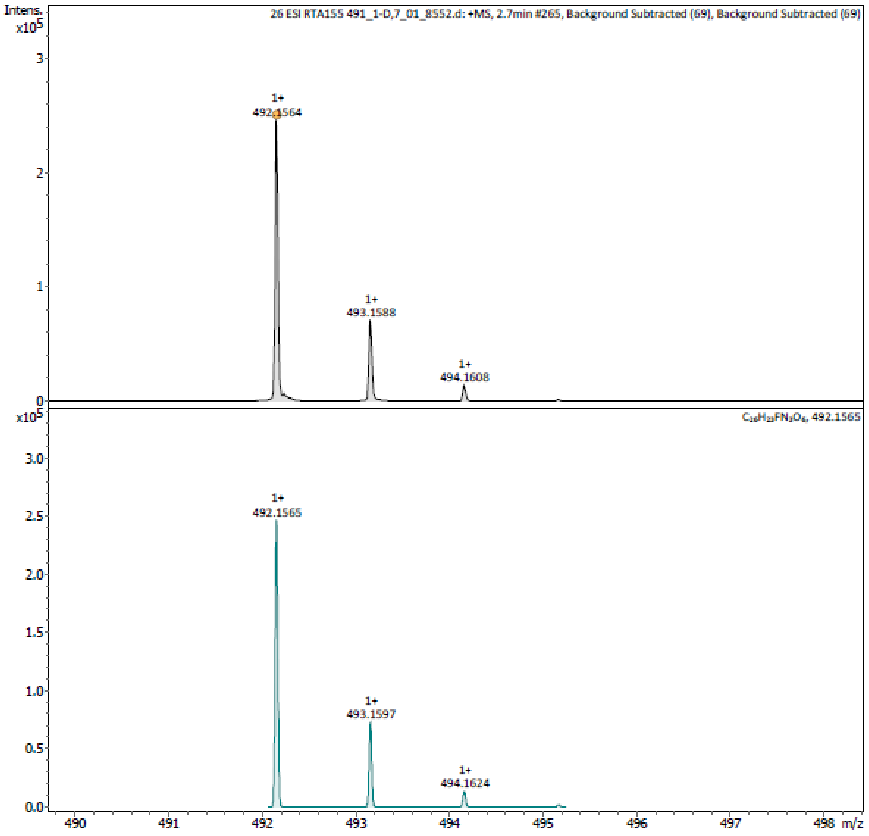

Meas. m/z	#	Ion Formula	Score	m/z	err [mDa]	err [ppm]	mSigma	rdb	e¯ Conf	N-Rule
492.1564	1	C24H21FN6O5	59.22	492.1552	-1.2	-2.4	3.2	17.5	odd	ok
	2	C26H23FN3O6	100.00	492.1565	0.1	0.3	4.6	17.0	even	ok
	3	C23H15FN13	39.77	492.1552	-1.2	-2.5	9.3	23.0	even	ok
	4	C28H25FO7	43.69	492.1579	1.5	3.0	10.5	16.5	odd	ok
	5	C25H17FN10O	64.48	492.1565	0.1	0.3	13.0	22.5	odd	ok

5(6)-(3,4,5-trimetoxiphen-1-yl)-2-(nitrophen-2-yl)-1H-benzimidazole **(10)**

^{1}H NMR (400 MHz, DMSO-d$_6$)

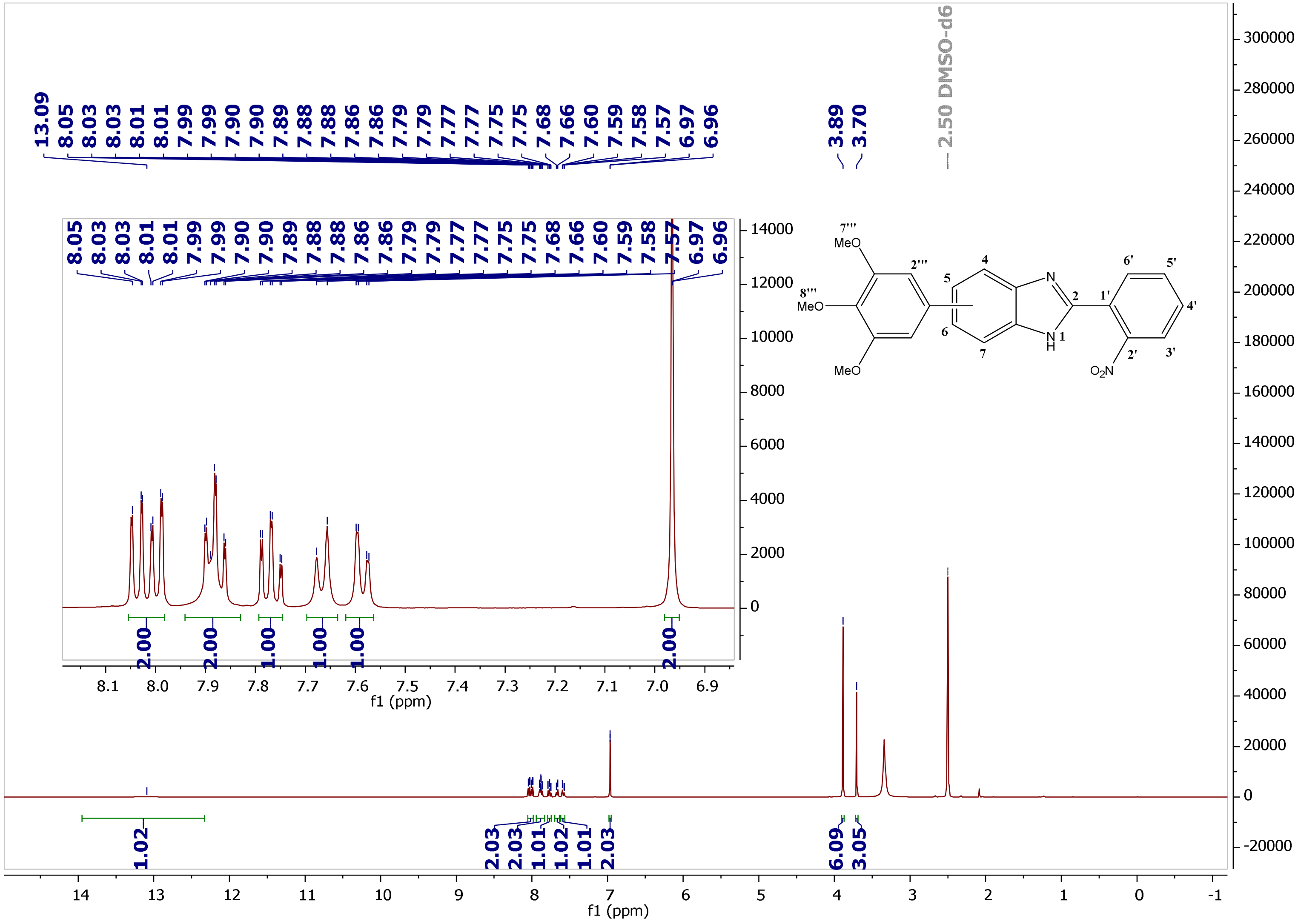

$^{13}C\{^{1}H\}$ NMR (101 MHz, DMSO-d_6)

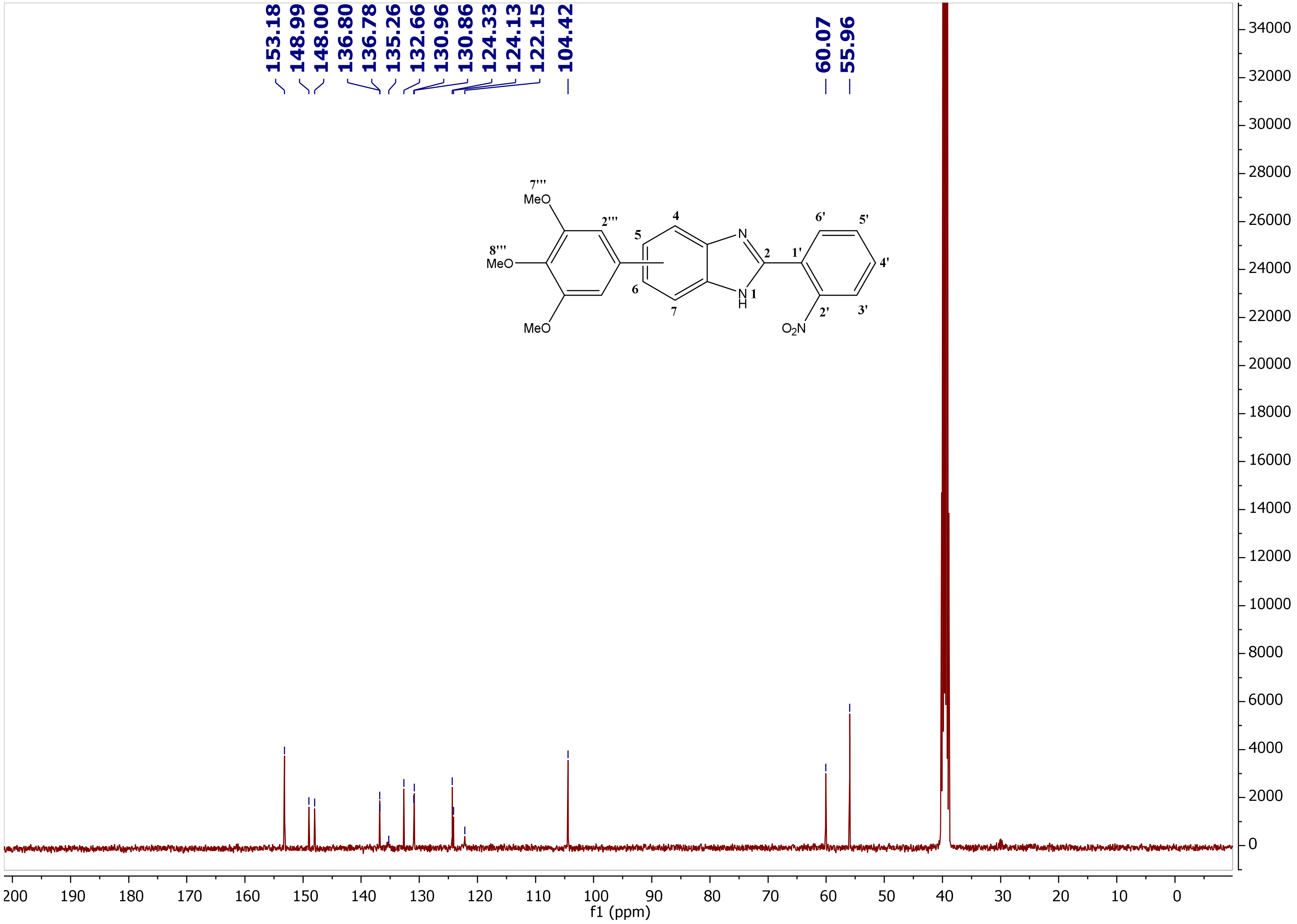

HRMS (ESI-TOF):

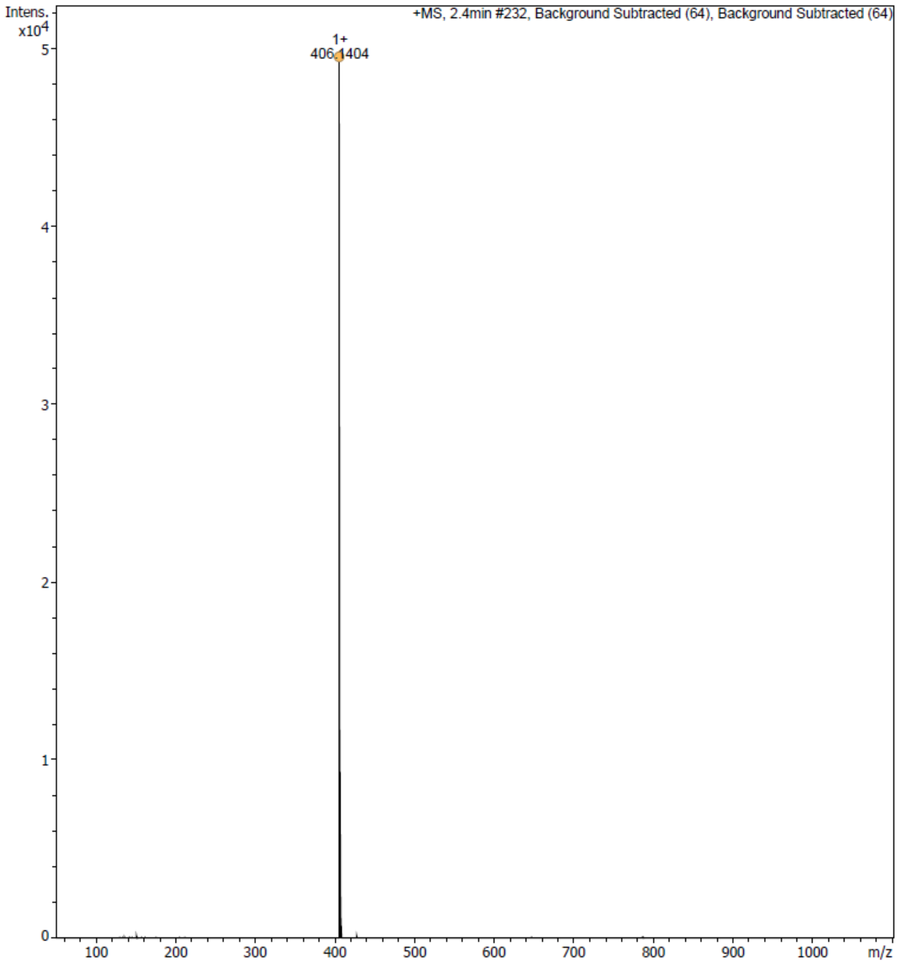

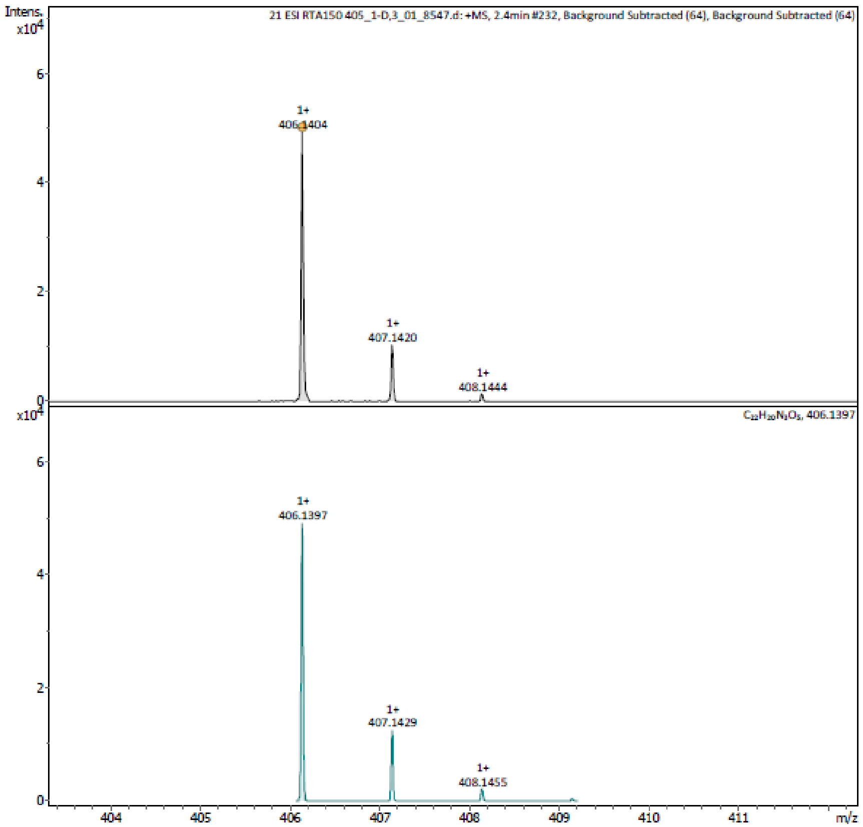

Meas. m/z	#	Ion Formula	Score	m/z	err [mDa]	err [ppm]	mSigma	rdb	e⁻ Conf	N-Rule
406.1404	1	C22H20N3O5	84.93	406.1397	-0.6	-1.5	26.6	15.0	even	ok
	2	C24H22O6	100.00	406.1411	0.7	1.8	29.3	14.5	odd	ok
	3	C8H12N19O2	55.39	406.1416	1.2	3.0	30.2	13.0	even	ok
	4	C21H14N10	73.37	406.1397	-0.6	-1.5	32.6	20.5	odd	ok
	5	C9H18N12O7	46.90	406.1416	1.2	3.0	36.7	7.5	odd	ok

5(6)-(3-fluoro-4-(methoxycarbonyl)phen-1-yl)-2-(nitrophen-2-yl)-1H-benzimidazole **(11)**

^{1}H NMR (400 MHz, acetone-d_6)

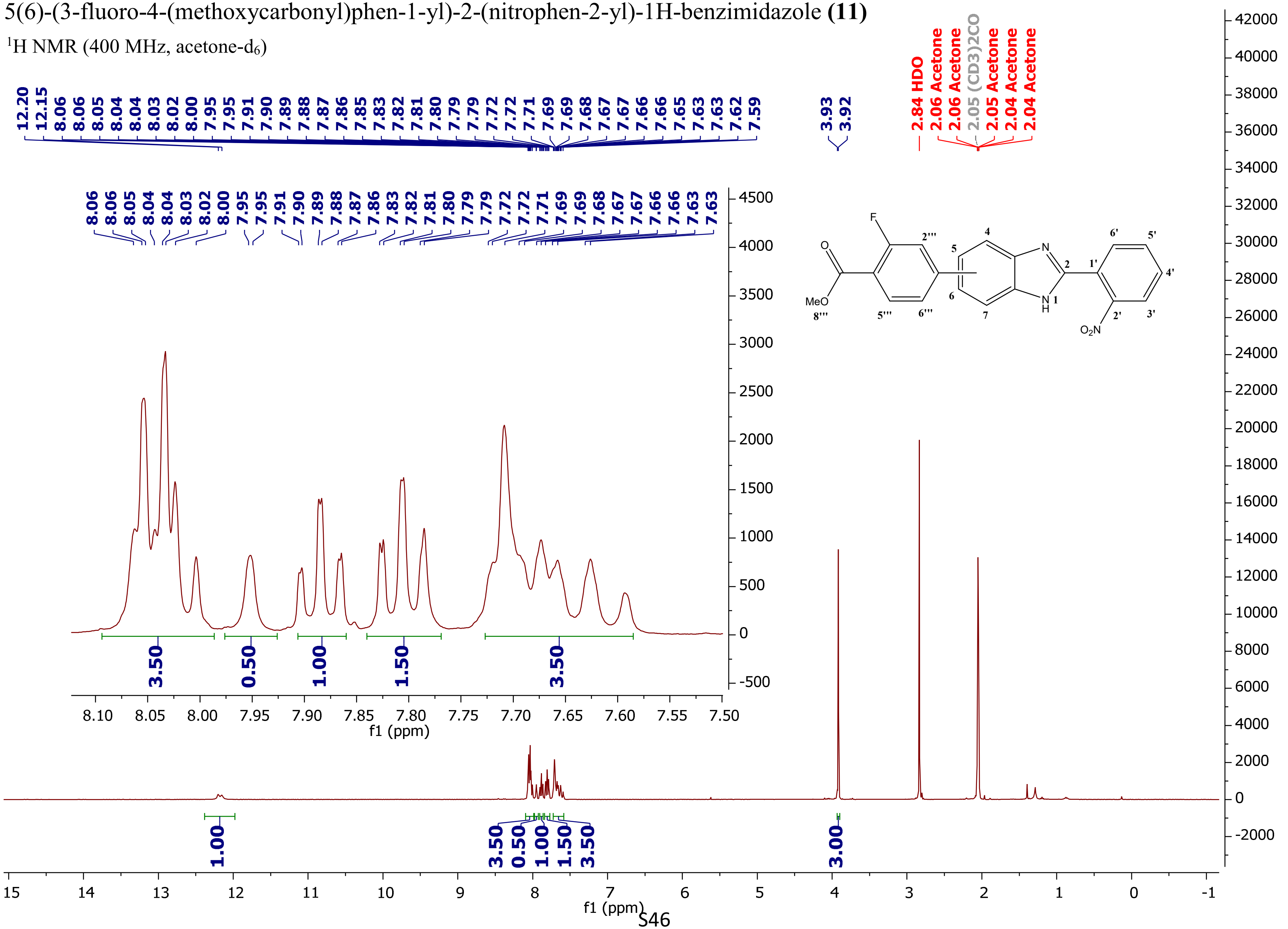

$^{13}C\{^{1}H\}$ NMR (101 MHz, acetone-d$_6$)

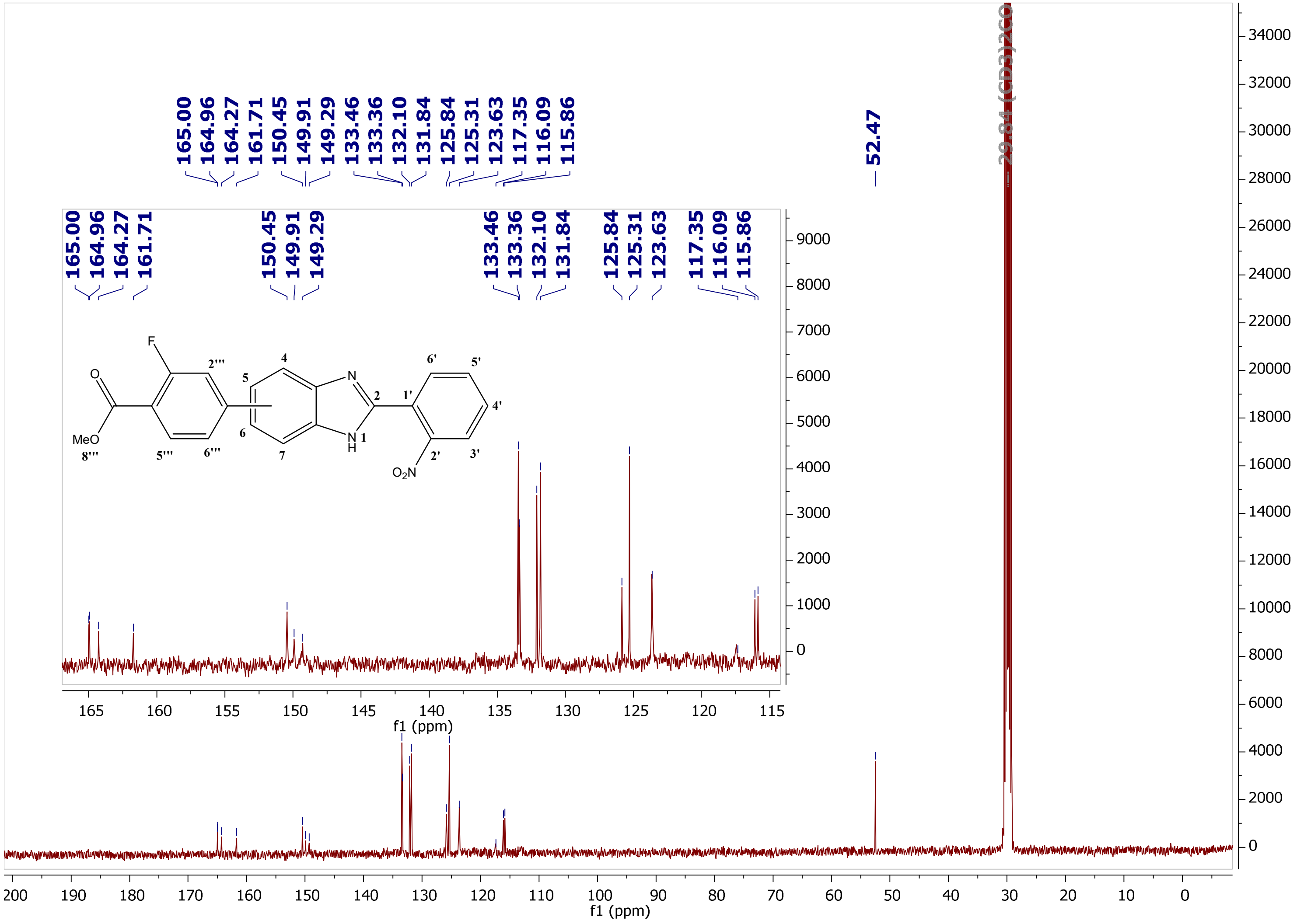

HRMS (ESI-TOF):

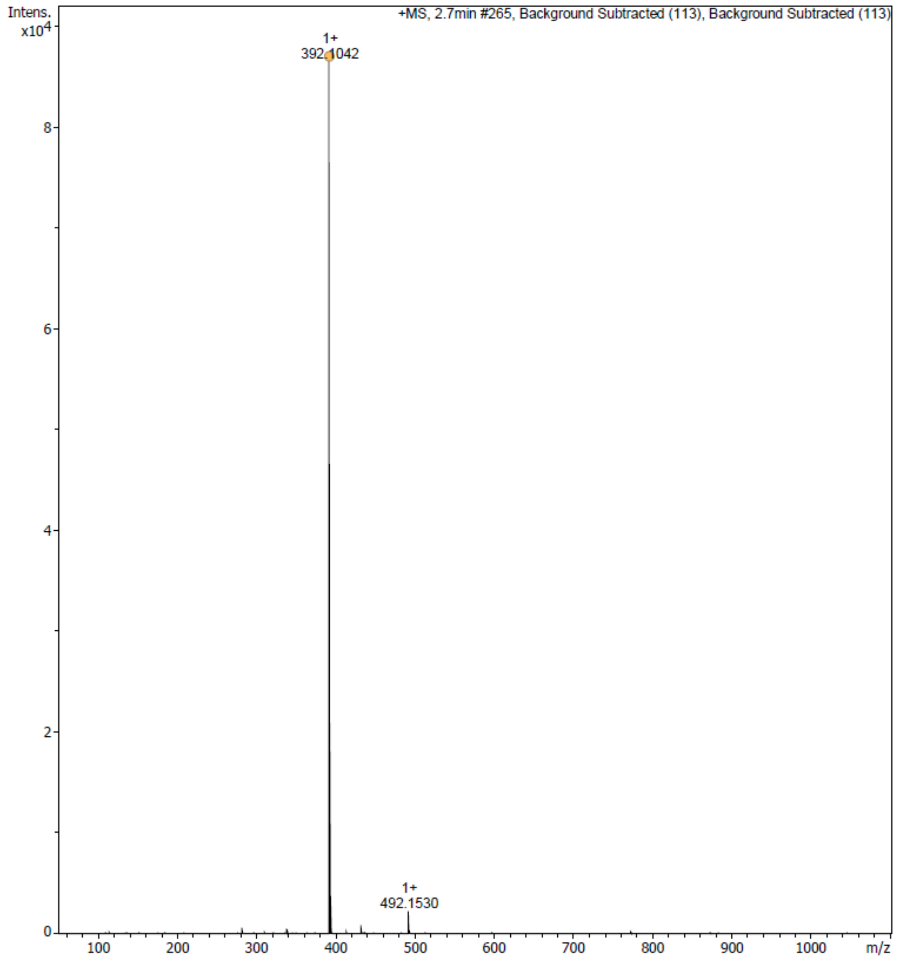

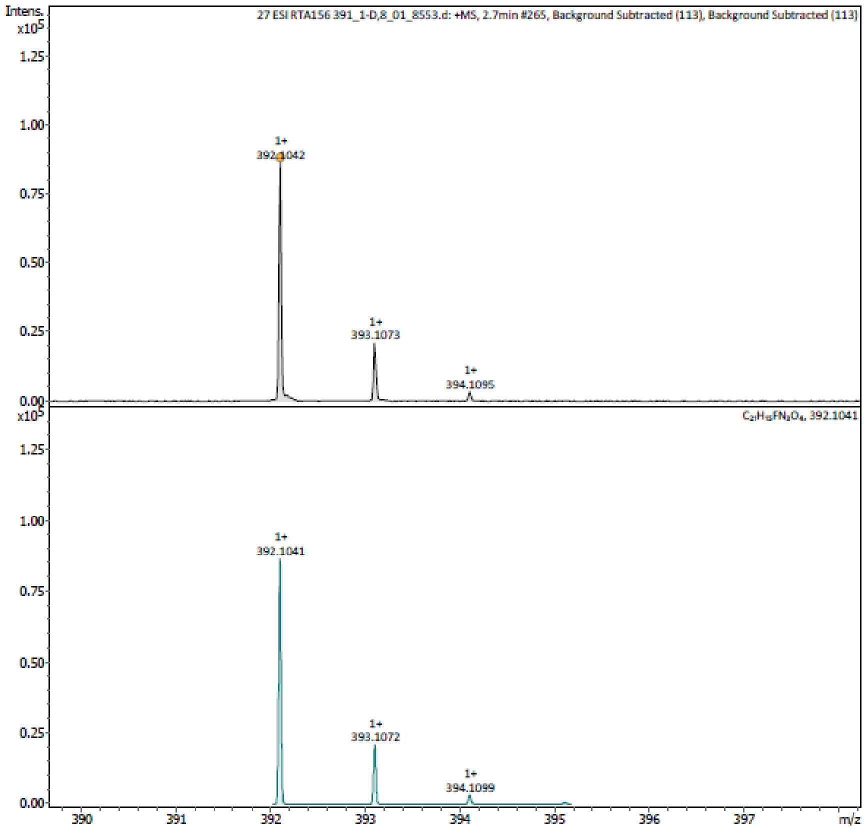

Meas. m/z	#	Ion Formula	Score	m/z	err [mDa]	err [ppm]	mSigma	rdb	e¯ Conf	N-Rule
392.1042	1	C21H15FN3O4	100.00	392.1041	-0.1	-0.2	1.4	16.0	even	ok
	2	C23H17FO5	50.06	392.1055	1.3	3.2	6.1	15.5	odd	ok
	3	C19H13FN6O3	43.97	392.1028	-1.4	-3.6	8.1	16.5	odd	ok
	4	C22H11FN7	44.01	392.1054	1.3	3.2	13.2	21.0	even	ok
	5	C24H14N3O3	41.15	392.1030	-1.2	-3.1	18.0	20.0	even	ok

N-(3-(methylsulfonyl)phenyl)-2-(nitrophen-2-yl)-1H-benzimidazol-5(6)-amine **(12)**

^{1}H NMR (400 MHz, acetone-d_6)

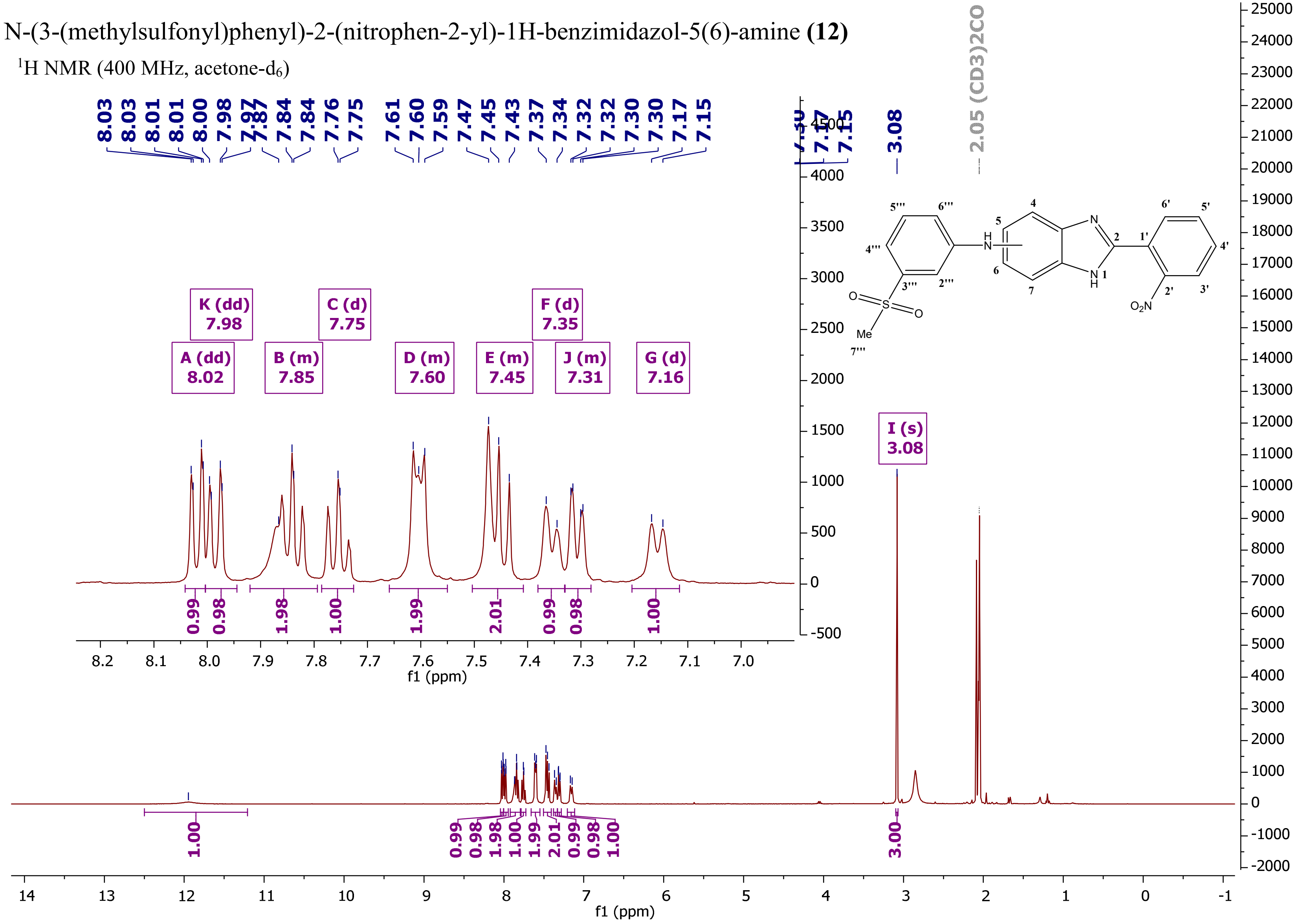

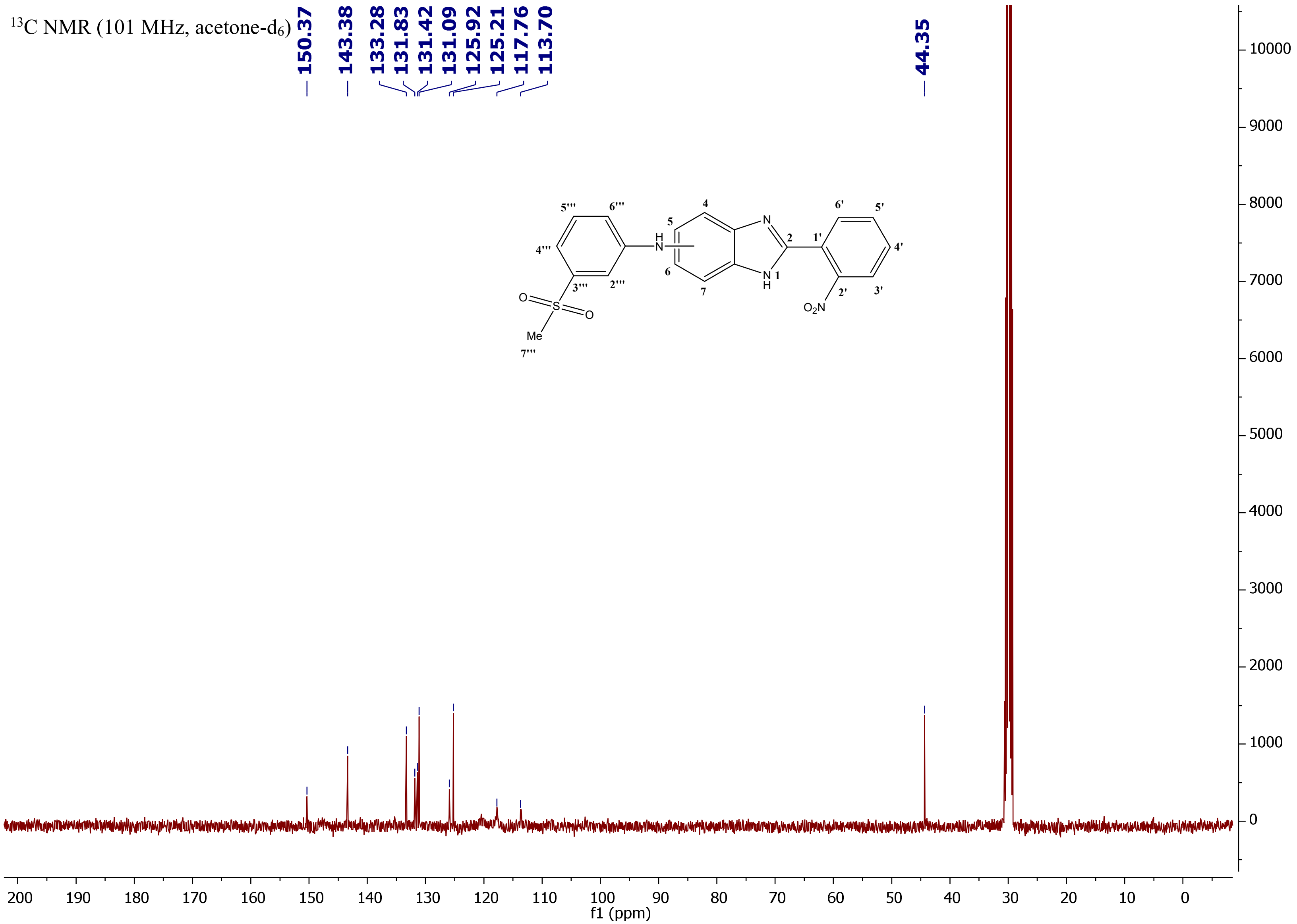

^{13}C NMR (101 MHz, acetone-d$_6$)
150.37
143.38
133.28
131.83
131.42
131.09
125.92
125.21
117.76
113.70
44.35
f1 (ppm)

HRMS (ESI-TOF):

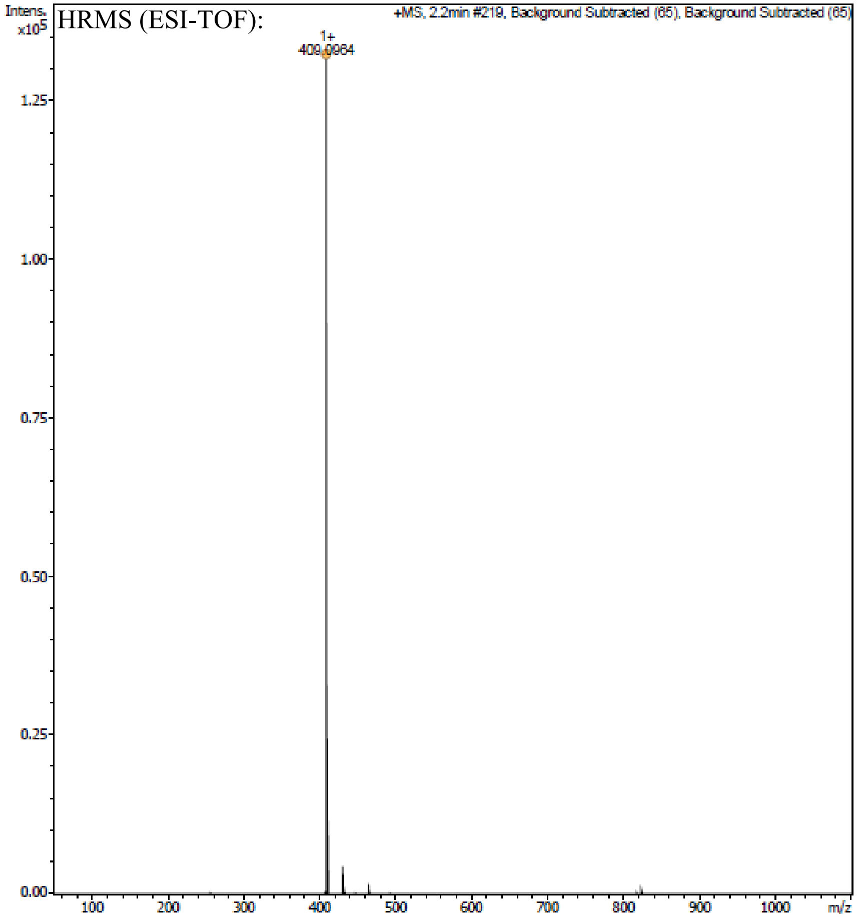

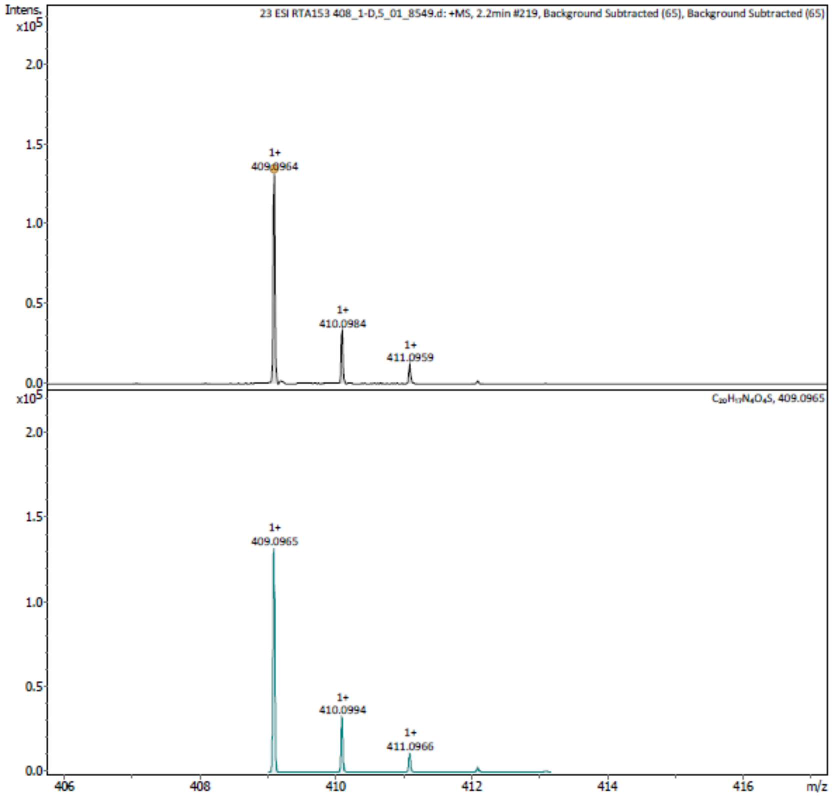

Meas. m/z	#	Ion Formula	Score	m/z	err [mDa]	err [ppm]	mSigma	rdb	e⁻ Conf	N-Rule
409.0964	1	C22H19NO5S	52.15	409.0978	1.4	3.4	2.4	16.5	odd	ok
	2	C20H17N4O4S	100.00	409.0965	0.1	0.2	5.6	17.0	even	ok
	3	C21H13N8S	46.45	409.0978	1.4	3.4	9.2	22.0	even	ok
	4	C18H15N7O3S	48.28	409.0952	-1.3	-3.1	11.5	17.5	odd	ok
	5	C19H21O8S	44.39	409.0952	-1.3	-3.1	15.9	12.0	even	ok

2-(aminophen-2-yl)-5(6)-(3,4,5-trimetoxiphen-1-yl)-1H-benzimidazole **(13)**

^{1}H NMR (400 MHz, CD_3OD)

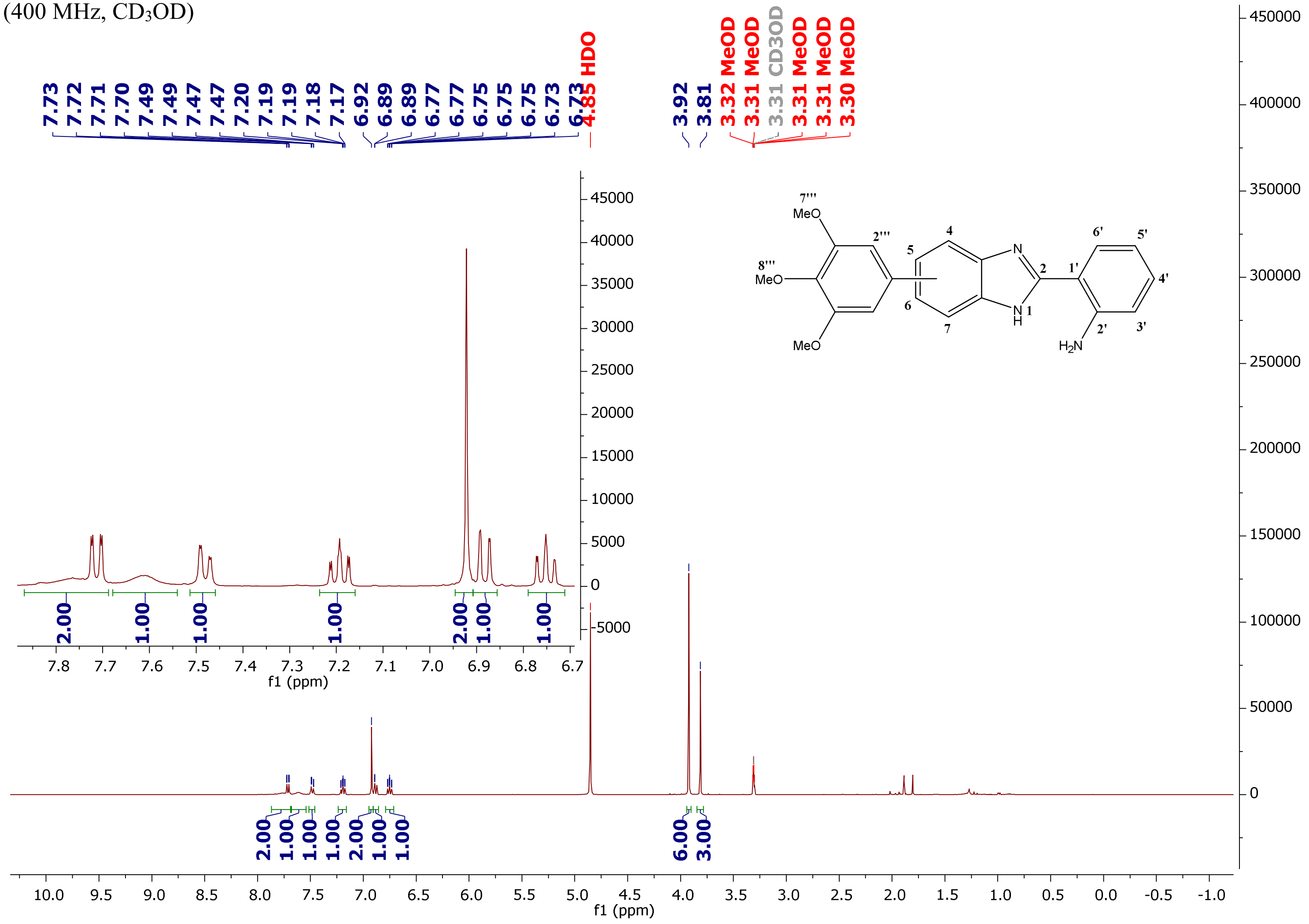

$^{13}C\{^1H\}$ NMR (101 MHz, CD_3OD)

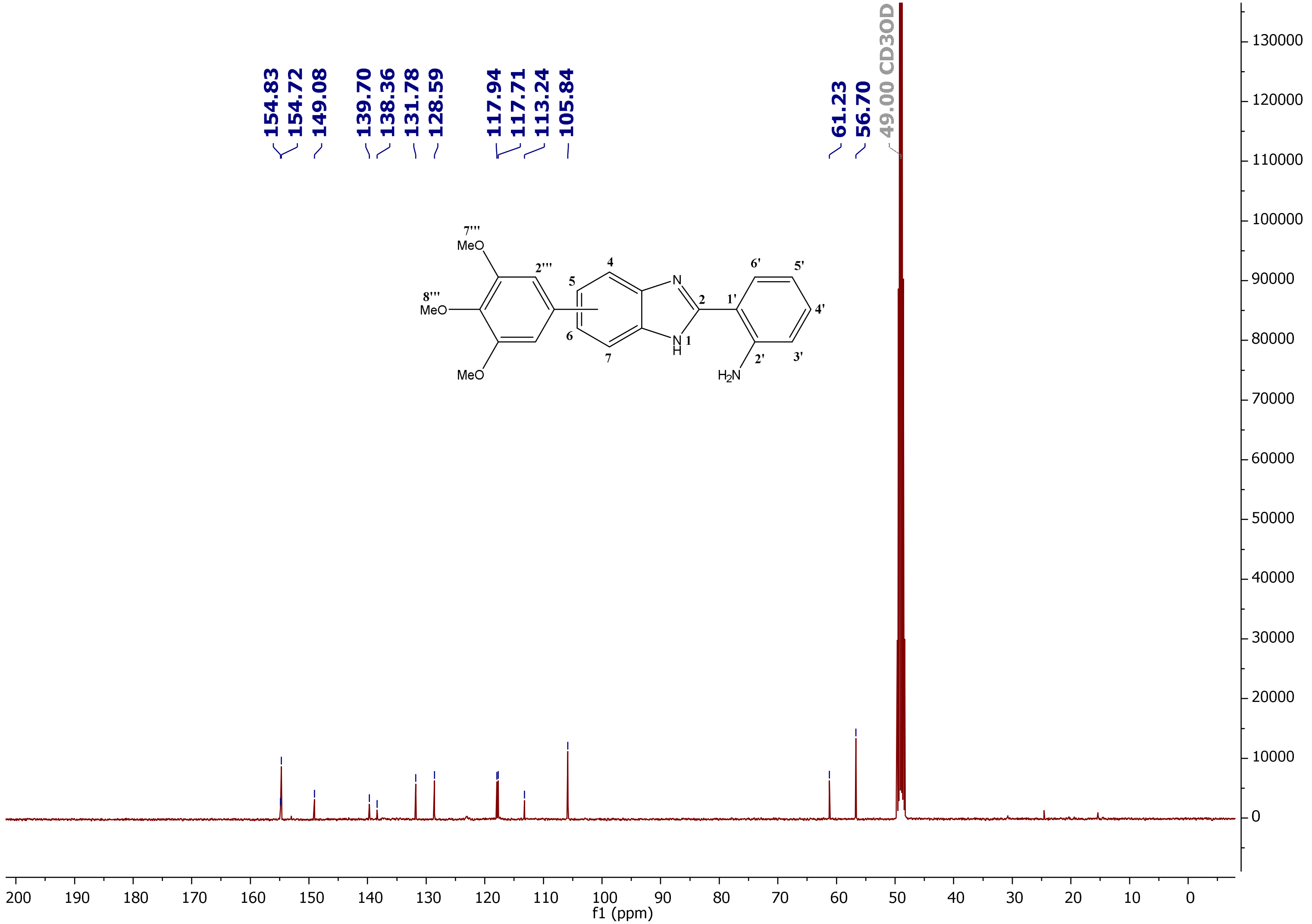

HRMS (ESI-TOF):

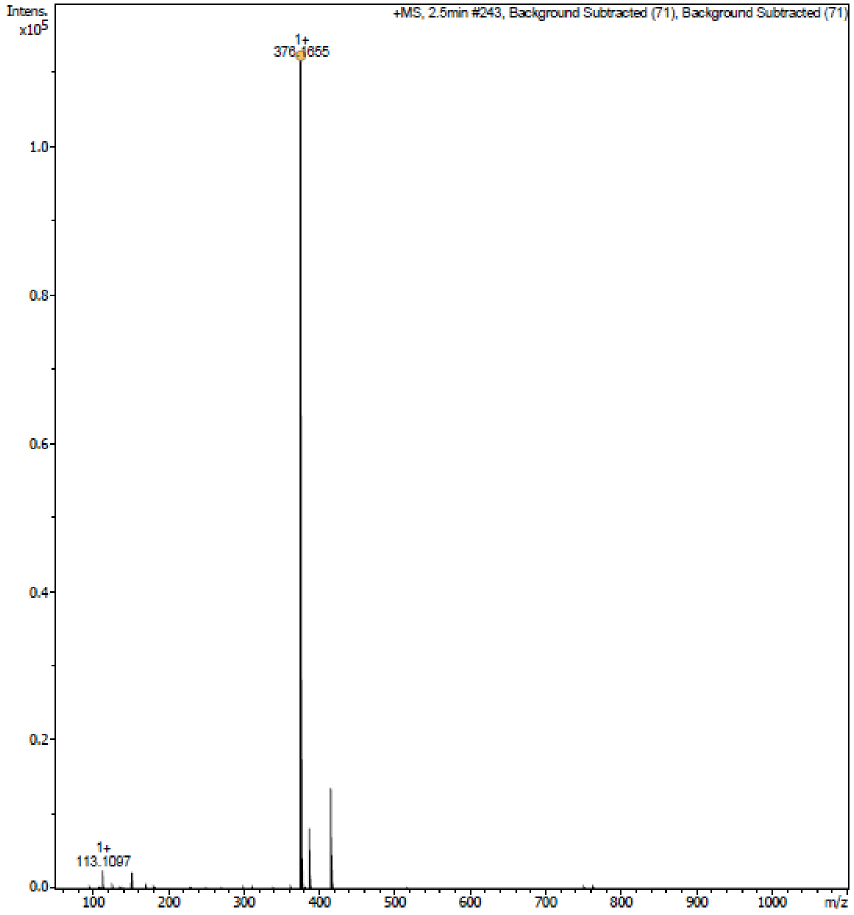

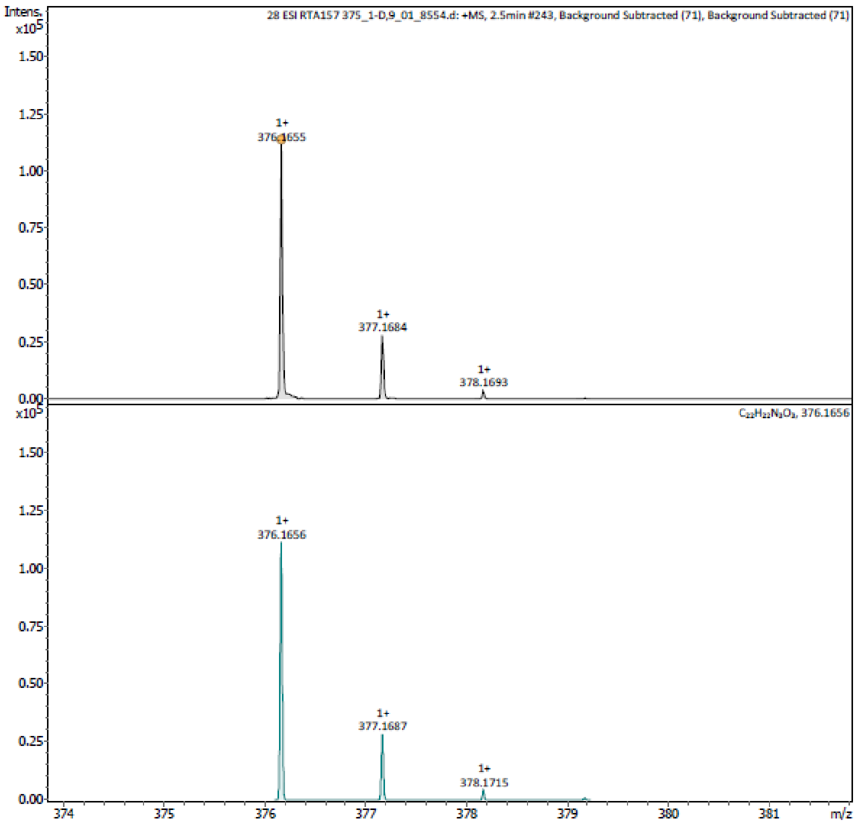

Meas. m/z	#	Ion Formula	Score	m/z	err [mDa]	err [ppm]	mSigma	rdb	e⁻ Conf	N-Rule
376.1655	1	C22H22N3O3	100.00	376.1656	0.0	0.1	2.0	14.0	even	ok
	2	C20H20N6O2	49.00	376.1642	-1.3	-3.4	6.2	14.5	odd	ok
	3	C24H24O4	44.48	376.1669	1.4	3.7	8.3	13.5	odd	ok

2-(aminophen-2-yl)-5(6)-(3-fluoro-4-(methoxycarbonyl)phen-1-yl)-1H-benzimidazole **(14)**

^{1}H NMR (400 MHz, DMSO-d_6)

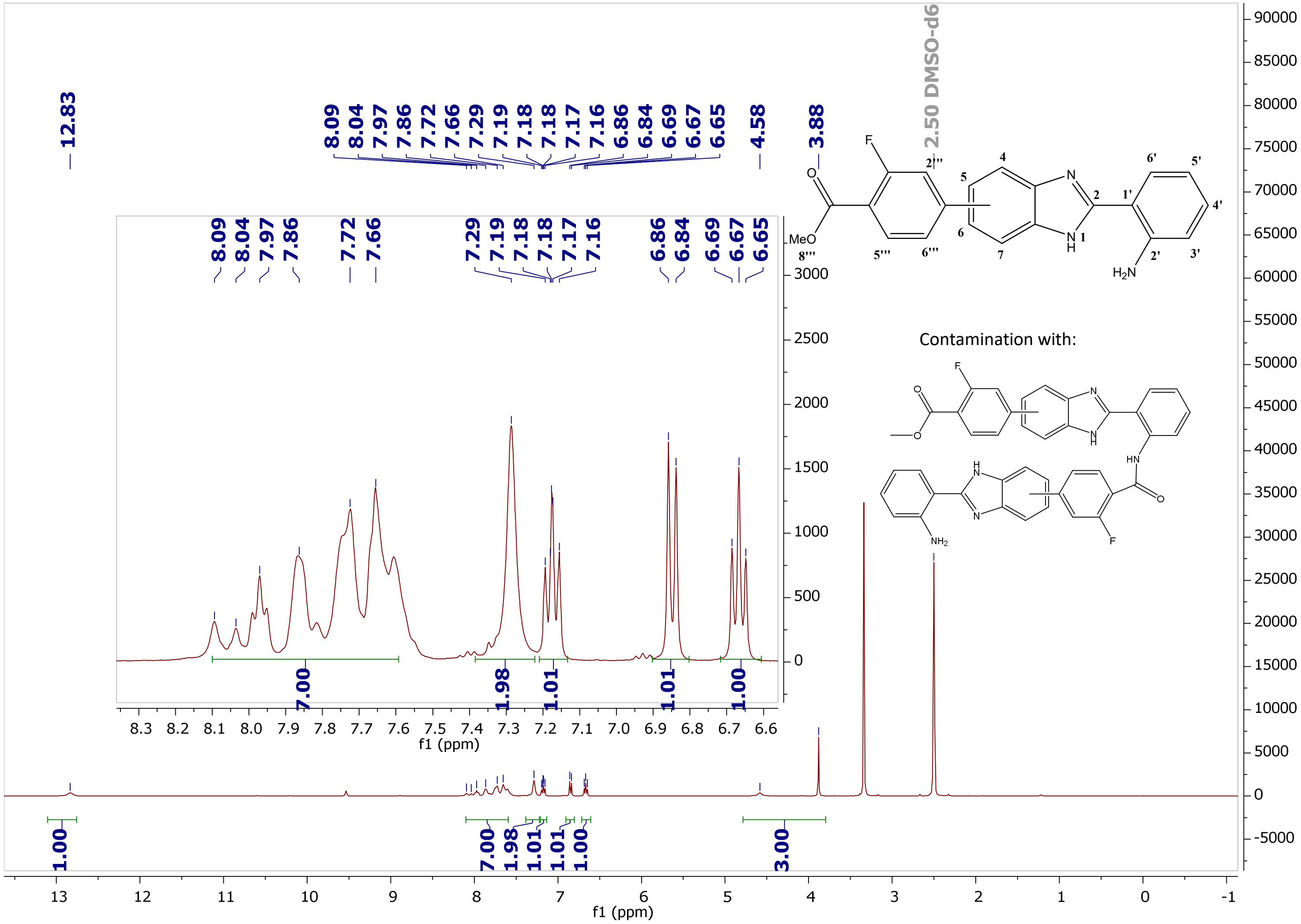

$^{13}C\{^1H\}$ NMR (101 MHz, DMSO-d_6)

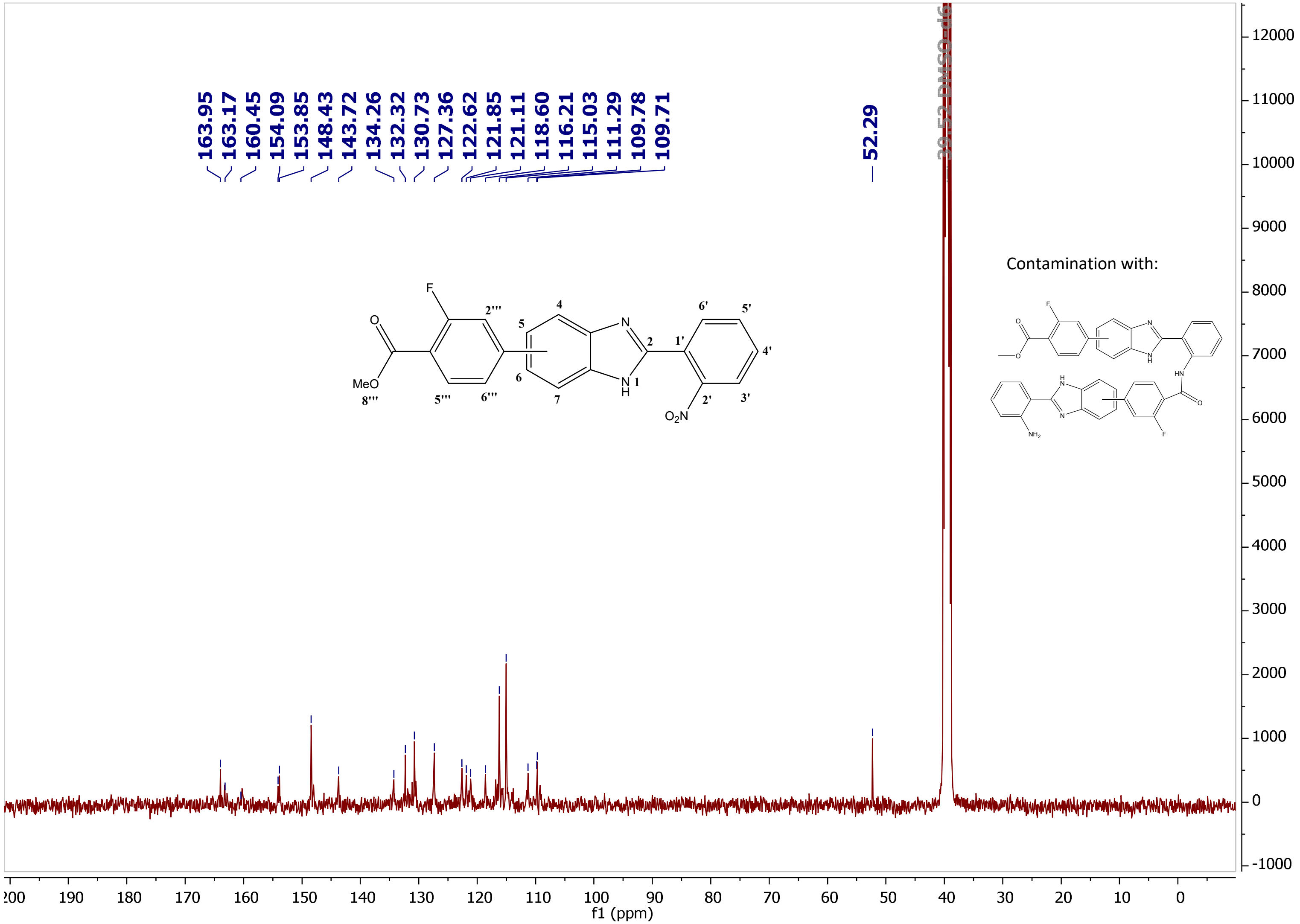

HRMS (ESI-TOF):

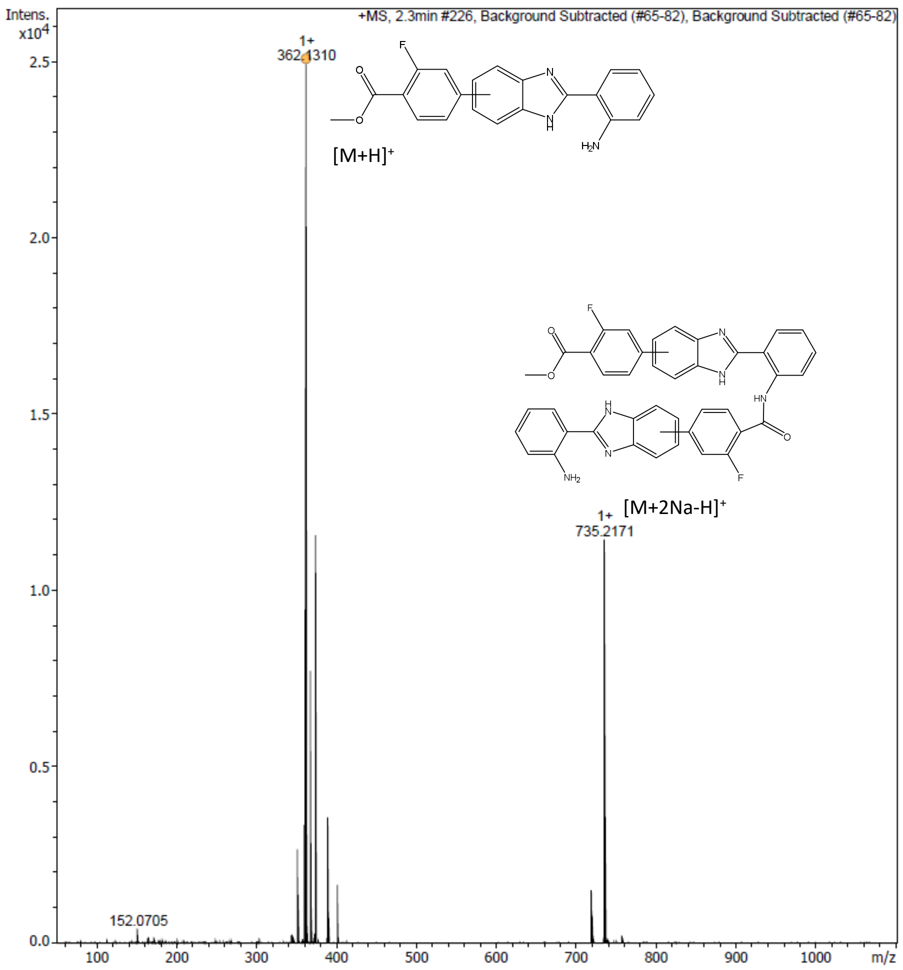

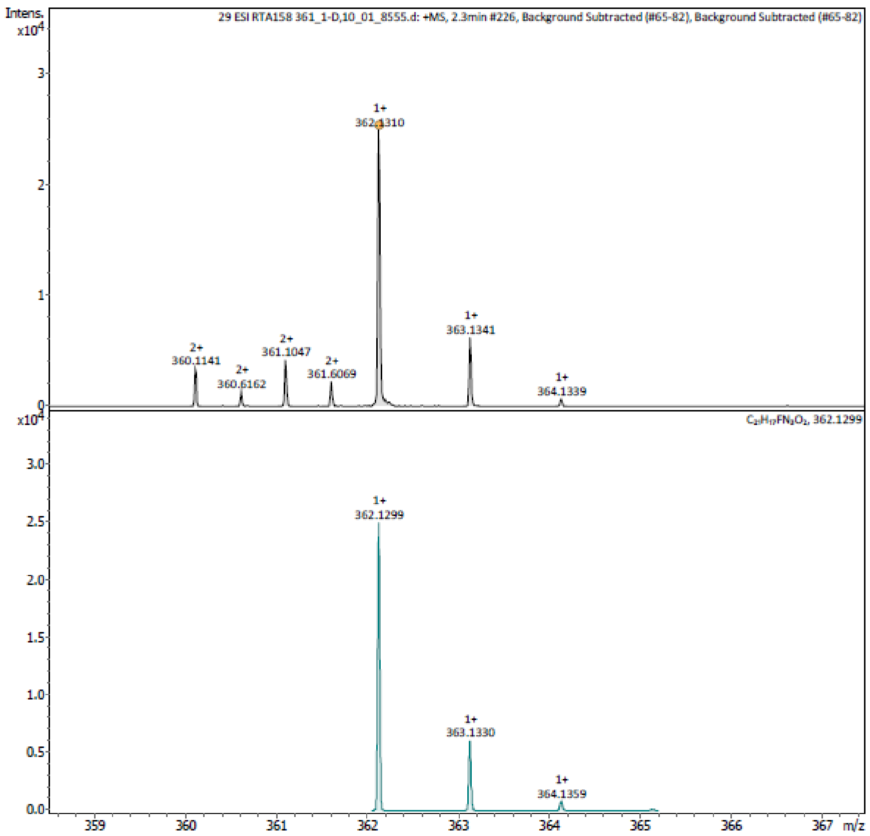

Meas. m/z	#	Ion Formula	Score	m/z	err [mDa]	err [ppm]	mSigma	rdb	e¯ Conf	N-Rule
362.1310	1	C21H17FN3O2	64.44	362.1299	-1.1	-3.1	6.3	15.0	even	ok
	2	C23H19FO3	100.00	362.1313	0.2	0.7	6.6	14.5	odd	ok
	3	C26H18O2	52.65	362.1301	-0.9	-2.5	22.3	18.5	odd	ok
	4	C13H16N9O4	31.53	362.1320	0.9	2.6	42.0	11.0	even	ok
	5	C11H14N12O3	34.24	362.1306	-0.4	-1.1	48.7	11.5	odd	ok

2-(aminophen-2-yl)-N-(3-(methylsulfonyl)phenyl)-1H-benzimidazol-5(6)-amine **(15)**

^{1}H NMR (400 MHz, CD_3OD)

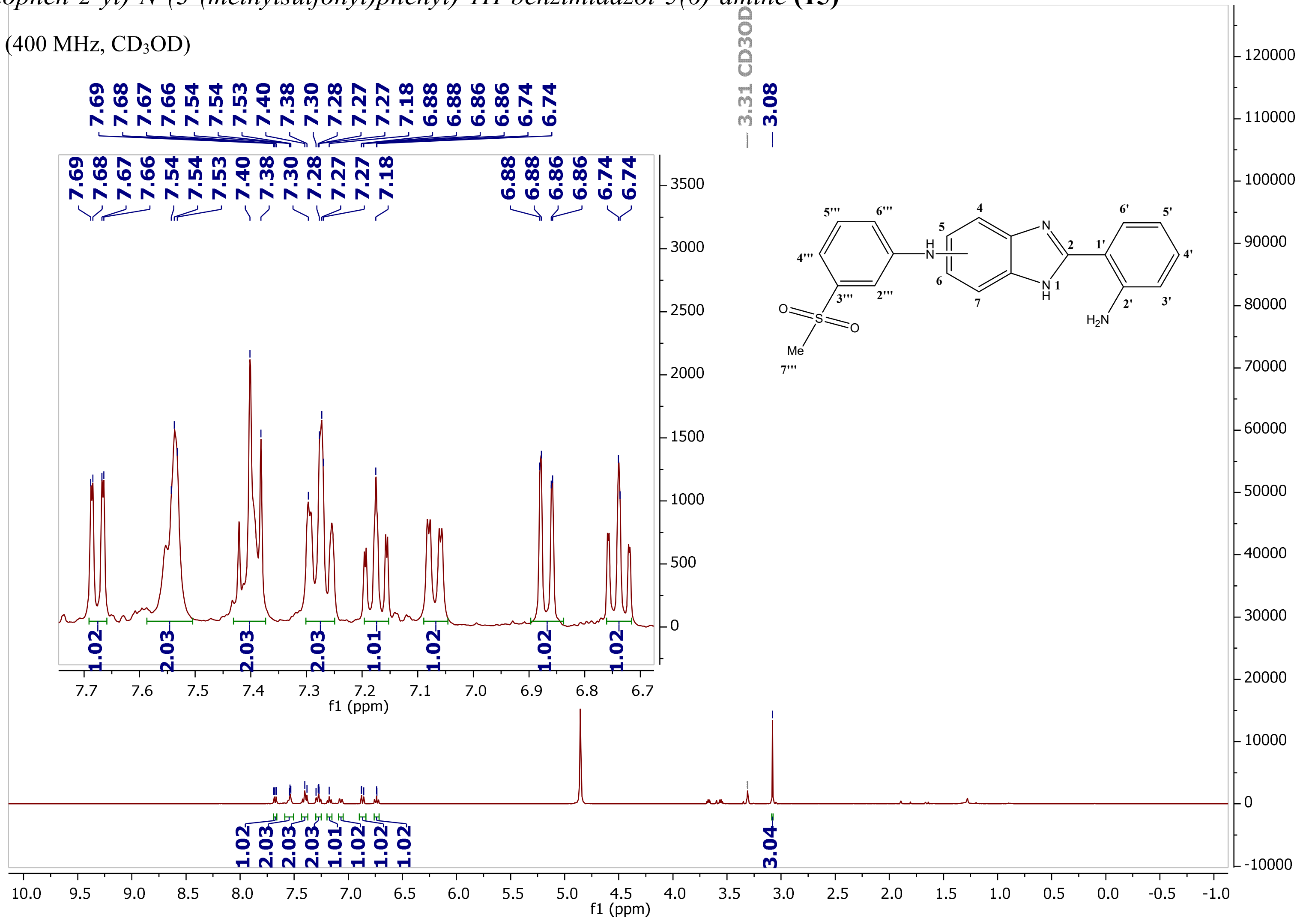

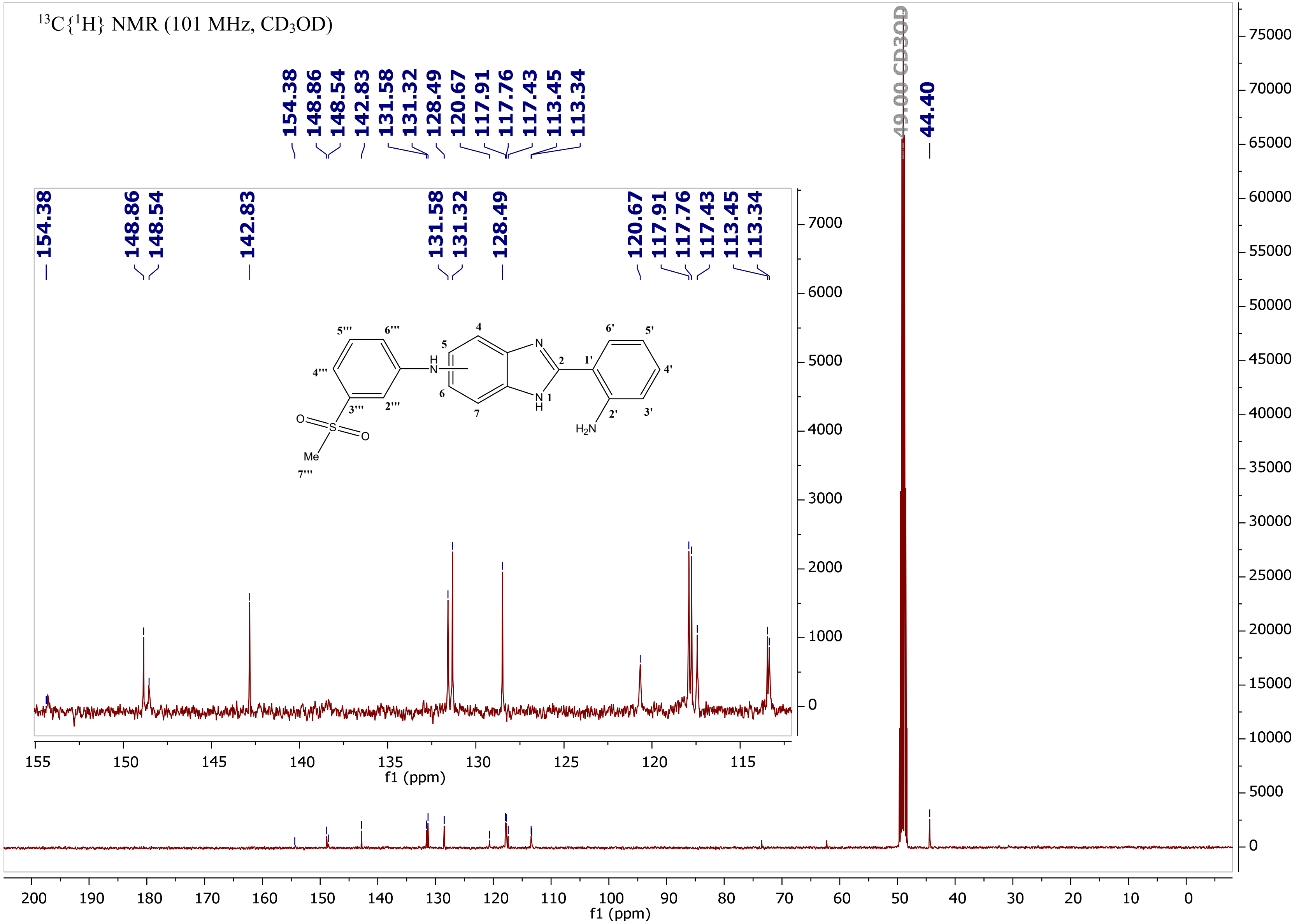

$^{13}C\{^1H\}$ NMR (101 MHz, CD_3OD)
154.38
148.86
148.54
142.83
131.58
131.32
128.49
120.67
117.91
117.76
117.43
113.45
113.34
49.00 CD3OD
44.40
f1 (ppm)

HRMS (ESI-TOF):

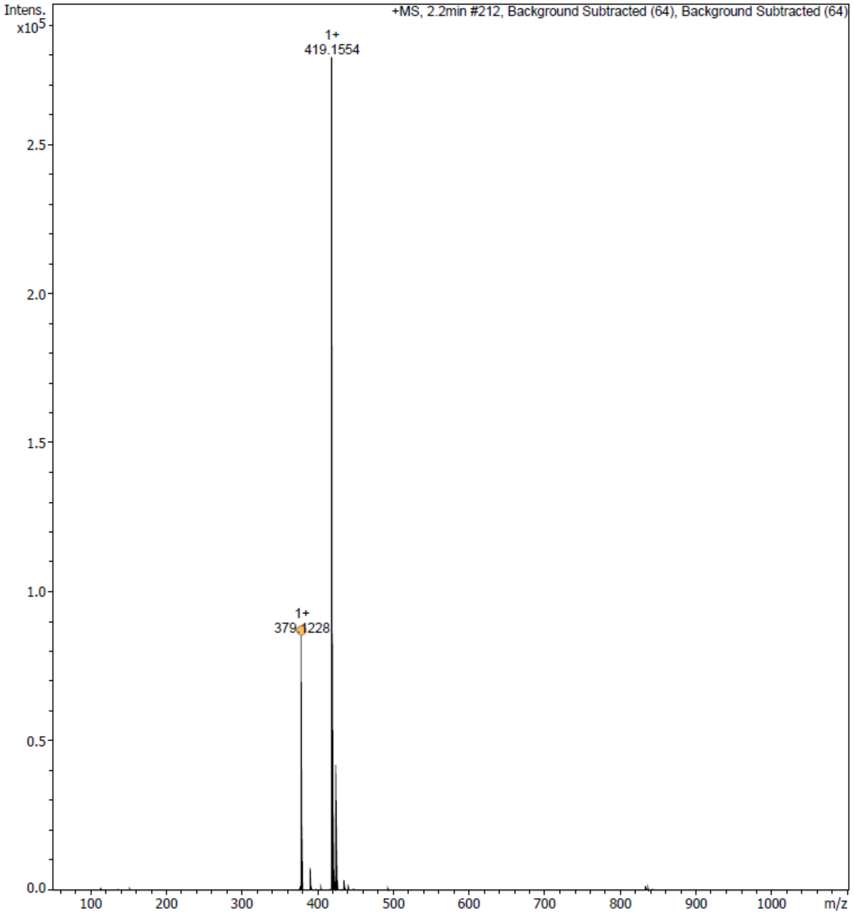

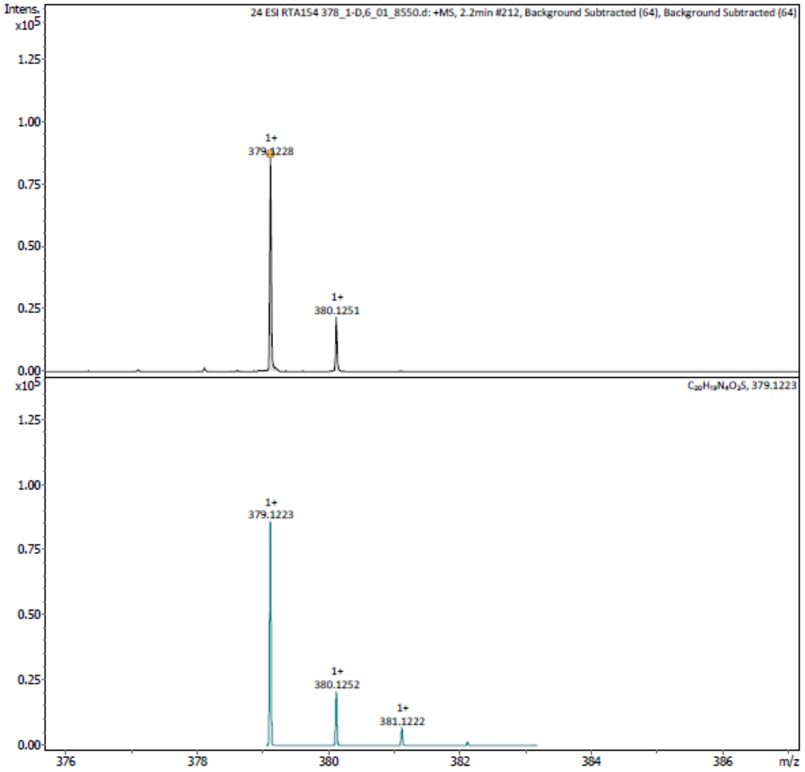

Meas. m/z	#	Ion Formula	Score	m/z	err [mDa]	err [ppm]	mSigma	rdb	e¯ Conf	N-Rule
379.1228	1	C13H11N14O	70.94	379.1235	0.7	1.8	37.3	16.0	even	ok
	2	C20H19N4O2S	100.00	379.1223	-0.5	-1.2	39.6	16.0	even	ok
	3	C22H21NO3S	76.44	379.1237	0.9	2.3	41.5	15.5	odd	ok
	4	C28H15N2	77.85	379.1230	0.2	0.5	42.6	23.0	even	ok
	5	C11H9N17	62.11	379.1221	-0.6	-1.7	43.0	16.5	odd	ok
